# Potential of Large Language Models in Health Care: Delphi Study

**DOI:** 10.2196/52399

**Published:** 2024-05-13

**Authors:** Kerstin Denecke, Richard May, LLMHealthGroup, Octavio Rivera Romero

**Affiliations:** 1 Bern University of Applied Sciences Biel Switzerland; 2 Harz University of Applied Sciences Wernigerode Germany; 3 see Acknowledgments; 4 Instituto de Ingeniería Informática (I3US) Universidad de Sevilla Sevilla Spain; 5 Department of Electronic Technology Universidad de Sevilla Sevilla Spain

**Keywords:** large language models, LLMs, health care, Delphi study, natural language processing, NLP, artificial intelligence, language model, Delphi, future, innovation, interview, interviews, informatics, experience, experiences, attitude, attitudes, opinion, perception, perceptions, perspective, perspectives, implementation

## Abstract

**Background:**

A large language model (LLM) is a machine learning model inferred from text data that captures subtle patterns of language use in context. Modern LLMs are based on neural network architectures that incorporate transformer methods. They allow the model to relate words together through attention to multiple words in a text sequence. LLMs have been shown to be highly effective for a range of tasks in natural language processing (NLP), including classification and information extraction tasks and generative applications.

**Objective:**

The aim of this adapted Delphi study was to collect researchers’ opinions on how LLMs might influence health care and on the strengths, weaknesses, opportunities, and threats of LLM use in health care.

**Methods:**

We invited researchers in the fields of health informatics, nursing informatics, and medical NLP to share their opinions on LLM use in health care. We started the first round with open questions based on our strengths, weaknesses, opportunities, and threats framework. In the second and third round, the participants scored these items.

**Results:**

The first, second, and third rounds had 28, 23, and 21 participants, respectively. Almost all participants (26/28, 93% in round 1 and 20/21, 95% in round 3) were affiliated with academic institutions. Agreement was reached on 103 items related to use cases, benefits, risks, reliability, adoption aspects, and the future of LLMs in health care. Participants offered several use cases, including supporting clinical tasks, documentation tasks, and medical research and education, and agreed that LLM-based systems will act as health assistants for patient education. The agreed-upon benefits included increased efficiency in data handling and extraction, improved automation of processes, improved quality of health care services and overall health outcomes, provision of personalized care, accelerated diagnosis and treatment processes, and improved interaction between patients and health care professionals. In total, 5 risks to health care in general were identified: cybersecurity breaches, the potential for patient misinformation, ethical concerns, the likelihood of biased decision-making, and the risk associated with inaccurate communication. Overconfidence in LLM-based systems was recognized as a risk to the medical profession. The 6 agreed-upon privacy risks included the use of unregulated cloud services that compromise data security, exposure of sensitive patient data, breaches of confidentiality, fraudulent use of information, vulnerabilities in data storage and communication, and inappropriate access or use of patient data.

**Conclusions:**

Future research related to LLMs should not only focus on testing their possibilities for NLP-related tasks but also consider the workflows the models could contribute to and the requirements regarding quality, integration, and regulations needed for successful implementation in practice.

## Introduction

### Background

A large language model (LLM) is a machine learning model that encodes complex patterns of language use derived from vast quantities of input texts [1,2]. Modern LLMs use neural network architectures, typically enhanced with a transformer attention mechanism that captures associative relationships between words based on shared context [3]. They use attention or self-attention to identify how distant data elements influence and depend on one another. Specifically, transformers learn context by tracking relationships in sequential data such as words in a sentence. Typically, transformer-based models are trained in 2 phases: the *pretraining phase* focuses on generic representation learning, and the *transfer learning phase* focuses on adjusting the model to an application-specific prediction task [4]. The pretrained models, which are often trained on large data sets (eg, Wikipedia, Reddit, biomedical literature, or public medical data sets), are tuned to be used for a wider set of tasks and can be fine-tuned for specific tasks [5]. In this study, we consider LLMs that use transformers for tasks related to medical natural language processing (NLP) tasks.

First described in 2017 by researchers from Google [3], LLMs are very well suited to NLP [6,7] for tasks such as machine translation [8], document summarization [9], natural language generation [10], and emotion recognition [11]. For example, Yang et al [12] explored LLMs for clinical concept extraction. Specifically, they tested 4 architectures—Bidirectional Encoder Representations From Transformers (BERT) [13], Robustly optimized BERT approach, A lite BERT, and Efficiently Learning an Encoder that Classifies Token Replacements Accurately—and achieved *F*_1_-scores between 93% and 95%. The public has gained widespread awareness of LLMs starting in 2022 with the release of ChatGPT, which uses a generative pretrained transformer model. The studies on ChatGPT demonstrate a huge potential, but some have identified limitations [14]. For instance, Cocci et al [15] assessed ChatGPT as a tool for providing medical information to patients in the context of urology. They compared output generated by ChatGPT to that provided by a board-certified urologist. Although they concluded that this approach has “the potential to enhance health outcomes and patient satisfaction” [15], they also identified an inadequate appropriateness and quality of responses. Many use case–specific appraisals of LLM technology beyond the ones outlined here are becoming available. For this reason, it is useful to identify and summarize the overall potential benefits and risks of these technologies.

Several individual opinion papers on the strengths, weaknesses, opportunities, and threats (SWOT) of LLMs in general, and ChatGPT in particular, have been published recently. Farrokhnia et al [16] conducted a SWOT analysis and outlined ChatGPT’s strengths and weaknesses with a specific focus on education. In a systematic review, Garg et al [17] described articles on the use of ChatGPT in clinical practice and medical research to summarize the potential of ChatGPT in diagnosing and treating patients as well as its possible contributions to medical research. Lee et al [18] have also studied the benefits, limits, and risks of ChatGPT in the medical domain. Although multiple studies have assessed the quality of LLMs for various tasks in the health care domain [19-21], a comprehensive description of the potential benefits and risks of LLMs, along with their relative importance according to multiple participants from a range of settings, has yet to be provided.

We believe that aggregated researchers’ opinions are needed because individual studies can never reflect the entire potential and risks might have been overlooked. It is essential to ensure a responsible and safe use to protect patients’ interests and foster trust in artificial intelligence (AI)–driven health care technologies. Such work can guide future research and development efforts to effectively address specific health care challenges.

### Objectives

Overall, the objectives of our study were to seek researchers’ opinions on (1) the likelihood that LLMs will be adopted in health care and for what purposes, (2) the likely benefits of LLMs in health care, (3) the shortcomings and risks of LLM adoption in health care, (4) the requirements for the adoption of LLMs in health care, and (5) the reliability of LLMs in future health care.

Where there was substantial agreement among respondents, the responses were analyzed to identify the SWOT of LLMs and derive practical and research implications.

## Methods

### Overview

We followed a 2-step process. First, a modified Delphi method [22] was used to aggregate opinions on the potential and limitations of LLMs in health care. Second, to increase the practical relevance of the analysis, the results were aggregated into the SWOT of LLMs. The Delphi method is widely used to evaluate consensus, or lack thereof, among participants [23]. Similarly, the Delphi method has been considered suitable for exploratory idea generation on complex and multidisciplinary topics, especially if the objective of the research is the analysis of new trends [24-28]. In addition, this method has been widely applied to the health care domain [19-21,29-32].

**Figure 1:**
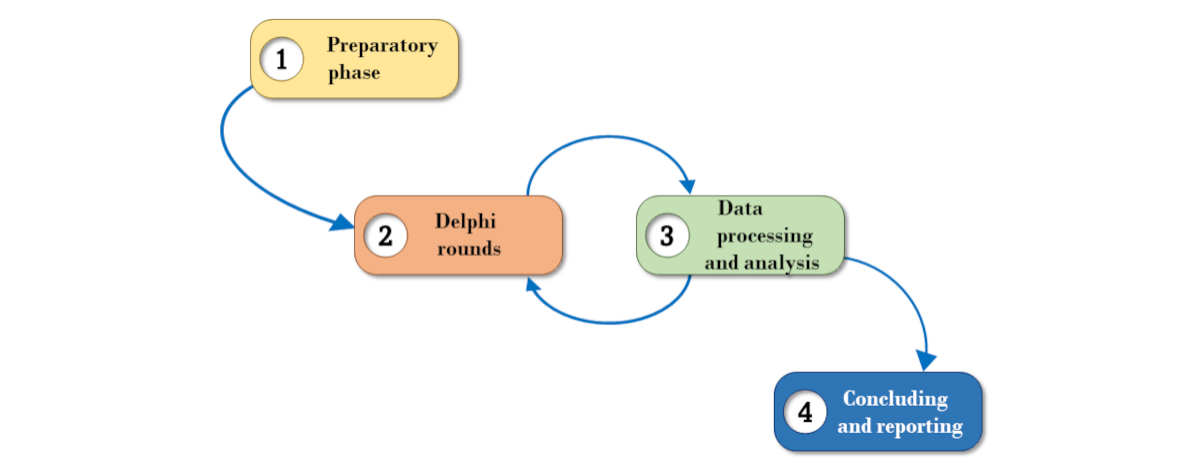
Stages of the study process.

Our iterative process consisted of four stages (Figure 1): (1) preparatory phase, (2) Delphi rounds, (3) data processing and analysis, and (4) concluding and reporting.

### Study Development

#### Preparatory Phase

The first stage in the defined Delphi process sought to develop the initial questionnaire for the first round. An iterative development process was followed. First, potential items for inclusion were identified by KD and RM by formulating questions and statements referring to demographic data as well as data related to the SWOT analysis (Textbox 1). Then, a third researcher (ORR) reviewed all the questions and provided feedback. Discrepancies were resolved through consensus among the 3 researchers.

**Questions driving the strengths, weaknesses, opportunities, and threats analysis.**

**Internal features**

- Strengths
  What are the advantages of large language models (LLMs) in health care?
  What are the achievements of LLMs in health care?
  Do LLMs already significantly improve digitized processes in health care?
- Weaknesses
  Which disadvantages of applying LLMs in health care exist?
  Are LLMs sufficiently developed to be actually reliable?
  Do LLMs support saving costs as well as ecological sustainability?

**External features**

- Opportunities
  What are current trends that support LLMs in health care?
  What are shortcomings in health care that can be exploited by LLMs?
  Do LLMs benefit from specific developments in both artificial intelligence and health care?
- Threats
  Which risks could emerge for health professionals, patient care, data protection, or general health IT due to the adoption of LLMs?
  Do LLMs contribute to discrimination in health care?
  Are health professionals sufficiently prepared for successfully adopting and using LLMs without losing competencies?
  Are LLMs useful and accepted by health professionals and patients?

We used a SWOT analysis [33] as it is a strategic planning method that takes into account both the internal and external features of a technology. Internal features refer to strengths (eg, scalability and innovative features) and weaknesses (eg, domain suitability and technical limitations) of the technology. In contrast, external features include opportunities (eg, regulatory support and market demands) as well as technology threats (eg, legal risks and competitor technologies). Overall, a SWOT analysis provides a basis for strategic decision-making by determining how to leverage strengths, address weaknesses, exploit opportunities, and mitigate threats. First, we collected questions driving our SWOT analysis (Textbox 1). These questions formed the basis to formulate questions for the Delphi questionnaire. After analyzing the answers to the Delphi questions, the answers in which agreement was achieved were used in turn to answer the SWOT analysis questions.

The final version of the initial (round 1) questionnaire consisted of 23 items organized into 5 sections (Multimedia Appendix 1). The first section (7 questions) collected participants’ demographic data. The second section included 8 open-ended questions on perceived benefits and shortcomings of using LLMs in health contexts. The third section consisted of 1 statement assessing the potential extent to which future health care could rely on LLMs. Responses on agreement were collected via a 5-point Likert scale with the following options: “very high extent, high extent, neither high nor small extent, small extent, and very small extent.” The fourth section consisted of 15 statements to be judged on a 5-point Likert scale with the following options—“strongly agree, agree, neither agree nor disagree, disagree, and strongly disagree”—assessing the future of LLMs. The last section included 2 statements with answers given on a 5-point Likert scale (“very high impact, impact beyond average, average impact, impact below average, and very low to no impact”) assessing the potential impact of using LLMs on health care.

A web version of the final round-1 questionnaire was created using the Microsoft Forms software (Microsoft Corp). A researcher (ORR) tested its usability and accessibility using several browsers in a laptop (Google Chrome version 116.0.5845.140, Microsoft Edge version 116.0.1938.62, and Mozilla Firefox version 46.0) and 2 updated browsers (Google Chrome and Samsung Internet Browser apps) in a Samsung Galaxy A52s and resolved these issues before deployment.

#### Delphi Rounds

After review of round-1 responses, some questions were reworded, and some items were added to the 5-point Likert questions for the round-2 questionnaire. Our approach was to carry all questions and items from a given round to the subsequent round even when agreement was reached in the previous round. We defined agreement as follows: when >75% of the researchers assigned a score equal to 4 or 5, agreement was achieved on this opinion. Participants were given the opportunity to change their minds during the subsequent round, and we were able to calculate the stability of their responses. Participants received the link to access the corresponding e-Delphi questionnaire via email. In addition, each participant received a feedback report including their responses to the questionnaire of the immediately previous round and figures showing the distribution of participants’ responses in percentages for each question and item.

#### Data Processing and Analysis

Quantitative data were analyzed using descriptive statistics in the Microsoft Excel software (Microsoft Corp). We calculated the median and IQR for the responses to each 5-point Likert question in each round. In addition, scores assigned to each item in 2 consecutive rounds were passed as parameters of the Wilcoxon matched-pairs signed rank test to estimate the stability of each item. This test was conducted using the R software (version 3.6.3; R Foundation for Statistical Computing).

The qualitative data were analyzed following a thematic analysis [34]. After reading several times for familiarization, KD and ORR independently coded all data. The codification was aimed at identifying some suggestions to modify the current questionnaire and any additional factors to be included as new items in the subsequent round. Coding was compared, and discrepancies were resolved through consensus among the coders. All factors were then grouped into themes or categories through consensus and added to the next round’s questionnaire.

We followed the recommendations of von der Gracht [35] for finding consensus. In this regard, we considered that agreement with an item was reached when the IQR of the participants’ responses to this item in the round was ≤1. The IQR is usually found to be a suitable consensus criterion for 4- or 5-unit scales. Following this criterion, we defined “agreement” with an item in a given round as the IQR of the participants’ responses being ≤1 and defined “disagreement” otherwise.

As it is recommended by von der Gracht [35], we also defined the stability between rounds as follows. Participants’ responses to an item in 2 consecutive rounds were considered stable when the median of these responses failed to show a statistically significant difference between the rounds. We used the Wilcoxon matched-pairs signed rank test to assess the stability in these responses. This test is commonly used to assess the stability of responses in 2 consecutive rounds in Delphi studies. Following these criteria, we considered that participants’ responses to an item in 2 consecutive rounds were stable when the results of the Wilcoxon matched-pairs signed rank test did not show a statistically significant difference and considered them unstable otherwise.

Finally, we defined the following stopping criteria for our Delphi process: (1) agreement was reached on all items and no new items were identified, (2) items for which agreement was not reached showed stability in 2 consecutive rounds, and (3) the panel size was reduced by >30% from the initial sample size.

#### Concluding and Reporting

We reported our study according to the Conducting and Reporting Delphi Studies guidelines (Multimedia Appendix 2) [36]. Although these were developed for consensus reporting in palliative care, they are relevant for other health care Delphi studies. The results tables include all statements and information on judgments, consensus, and stability.

### Delphi Participants

The Delphi method uses a purposely selected panel of participants to provide their feedback on a subject. The sample need not be statistically representative, and therefore, there is no standard method to calculate the panel size. Some guidelines have suggested the involvement of 15 participants [37]. However, Delphi studies in the health care domain have often involved 20 to 30 participants [36], and panels for this research typically have <50 participants. To achieve a gender-balanced sample size, we aimed to enroll 20 to 30 participants with representation from each of 6 continents (North America, South America, Europe, Africa, Asia, and Australia and Oceania). Specifically, we actively recruited health informaticians and researchers in the field of NLP in health care. We gathered members of the health informatics community from the International Medical Informatics Association Participatory Health and Social Media Working Group and the authors’ peer networks. In addition, researchers from the health NLP community were identified from research papers on health NLP, specifically on generative pretrained transformers and LLMs in health care, and invited via email. To acknowledge participation in this study, we offered coauthorship as a group to participants who completed all 3 Delphi rounds.

This study was conducted between April 2023 and June 2023. The recruitment of participants for round 1 was conducted from April 10, 2023, to May 1, 2023. For rounds 2 and 3, participants had 2 weeks to respond, with a reminder sent after 1 week. Round 2 was available for completion from May 15, 2023, to May 28, 2023; round 3 was to be completed from June 12, 2023, to June 25, 2023. In round 3, an additional reminder was sent 1 day before the deadline.

### Ethical Considerations

The study design was submitted to the ethics committee of the canton of Bern, which confirmed that no ethics approval was necessary (Req-2023-00427). Participants were invited to take part in the various rounds of the web-based Delphi study via email. Participation was voluntary, and they were informed that by submitting their responses to the form, they consented to their participation. Contact information for the corresponding author for any questions related to the study was provided both in the email and in the form header for each round.

## Results

### Characteristics of the Panel

An invitation was posted to the mailing list of the International Medical Informatics Association Participatory Health and Social Media Working Group, and 45 invitations were sent to individual researchers. The round-1 questionnaire had 28 respondents (Table 1). The round-2 questionnaire had 23 participants (return rate of 23/28, 82%), and the round-3 questionnaire had 21 participants (return rate of 21/23, 91%). Most of the participants in round 3 (18/21, 86%) had >10 years of experience in their field. Academia was the most represented sector (20/21, 95%). The panel engaged a diverse set of disciplines, with computer science or engineering being the most frequent work discipline followed by health informatics and medicine. The panel had participants from 3 continents: North America, Europe, and Australia and Oceania.

The panel reported a range of expertise in LLMs: 10% (2/21) were experts in LLMs, 29% (6/21) used their basic functions regularly, 29% (8/28) knew how they work, and 33% (7/21) had tested ChatGPT but had only basic knowledge of the underlying technology.

**Table 1:** Summary of participant characteristics.

Characteristics			Round 1 (n=28), n (%)		Round 2 (n=22), n (%)		Round 3 (n=21), n (%)
**Gender**							
	Female	7 (25)		5 (23)		5 (24)	
	Male	21 (75)		17 (77)		16 (76)	
	Nonbinary	0 (0)		0 (0)		0 (0)	
**Education or background**							
	Computer science or engineering	13 (46)		11 (50)		10 (48)	
	Health informatics	11 (39)		9 (41)		9 (43)	
	Medicine	8 (29)		7 (32)		7 (33)	
	Nursing	1 (4)		1 (5)		1 (5)	
	Other health sciences	3 (11)		2 (9)		2 (10)	
	Other	2 (7)		1 (5)		1 (5)	
**Years of work experience**							
	<5	3 (11)		2 (9)		2 (10)	
	5-10	1 (4)		1 (5)		1 (5)	
	>10	24 (86)		19 (86)		18 (86)	
**Sector^a^**							
	Academia	26 (93)		21 (95)		20 (95)	
	Public health sector	5 (18)		4 (18)		4 (19)	
	Private health sector	2 (7)		0 (0)		0 (0)	
**Continent**							
	Europe	21 (75)		16 (73)		15 (71)	
	Australia and Oceania	3 (11)		3 (14)		3 (14)	
	North America	4 (14)		4 (18)		4 (19)	
**Level of experience with LLMs^b^**							
	I am an expert. I know and apply nearly all of their functions.	3 (11)		2 (9)		2 (10)	
	I use their basic functions regularly.	7 (25)		6 (27)		6 (29)	
	I know how they work.	8 (29)		7 (32)		6 (29)	
	I tested ChatGPT but have only basic knowledge of the underlying technology.	9 (32)		7 (32)		7 (33)	
	I have no knowledge.	1 (4)		0 (0)		0 (0)	

^a^Some participants have several affiliations to different sectors.

^b^LLM: large language model.

### Incorporation of New Items Based on Responses to the Open-Ended Questions

**Figure 2:**
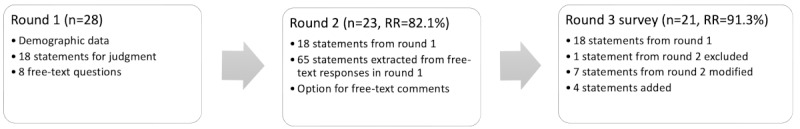
Data collection in the Delphi study. RR: response rate.

The responses to the open-ended questions in round 1 led to 65 new items for inclusion in rounds 2 and 3. A total of 4 new items were added in the round-3 questionnaire based on the responses to the open-ended questions. One statement was excluded in round 3 as it was already covered by another statement. In total, 7 items of round 2 were adapted according to the participants’ input. Figure 2 presents the evolution of the questionnaire in each round. The complete list of times in rounds 2 and 3 are available in Multimedia Appendix 3. The evolution of agreement in rounds 2 and 3 are available in Multimedia Appendix 4.

### Perceived Likelihood That LLMs Will Support Health Care Tasks

The items referring to the perceived likelihood that LLMs will support health care tasks were grouped into 3 dimensions: support in clinical tasks, documentation tasks, and medical research and education. Table 2 presents the results obtained in the final round. Consensus was not reached for only 1 item, “Design of chemical compositions of new drugs,” which belongs to the medical research and education dimension. However, only 57% (12/21) of the participants agreed that the likelihood of these systems supporting this task would be high (score of 4 or 5), with this being the item with the lowest percentage. A total of 95% (19/20) of the evaluated items were scored with a high likelihood of being supported by LLMs. Among all these tasks, all the participants agreed that LLM-based systems will act as virtual health assistants for patient education.

**Table 2:** Perceived likelihood that large language models will support health care tasks.

Item		Agreement and disagreement	Scores of 4 or 5 (n=21), n (%)	Scores of 5 (n=21), n (%)	Stability
**Clinical tasks**					
	Virtual health assistant for patients (education)	Agreement	21 (100)	9 (43)	Yes
	Automatic follow-up in chronic diseases	Agreement	20 (95)	11 (52)	Yes
	Virtual health assistant for patients (medical assistance and information)	Agreement	20 (95)	8 (38)	Yes
	Prediction of risk of disease development	Agreement	20 (95)	8 (38)	Yes
	Virtual health assistant for patients (answering queries)	Agreement	19 (91)	14 (67)	Yes
	Diagnostic process	Agreement	19 (91)	9 (43)	Yes
	Patient triage	Agreement	18 (86)	7 (33)	Yes
	Automatic treatment plan generation	Agreement	17 (81)	5 (24)	Yes
	Verbalizing interactions	Agreement	16 (76)	5 (24)	Yes
**Documentation tasks**					
	Automatic clinical encoding	Agreement	20 (95)	12 (57)	Yes
	Virtual health assistants for administrative tasks	Agreement	20 (95)	9 (43)	Yes
	Summarization	Agreement	19 (91)	7 (33)	Yes
	Automatic structuring of clinical narratives	Agreement	19 (91)	7 (33)	Yes
	Medical charting assistance	Agreement	18 (86)	8 (38)	Yes
	Generation of layperson summaries	Agreement	17 (81)	5 (24)	Yes
**Medical research and education**					
	Literature review and research	Agreement	19 (91)	10 (48)	Yes
	Clinical trial matching	Agreement	19 (91)	8 (38)	Yes
	Development of educational resources	Agreement	18 (86)	6 (29)	Yes
	Automatic generation of guidelines	Agreement	16 (76)	5 (24)	Yes
	Design of the chemical compositions of new drugs	Disagreement	12 (57)	3 (14)	N/A^a^

^a^N/A: not applicable.

### Benefits of Using LLMs in Health Care

Consensus was not reached in only 1 of the items evaluated regarding the benefits of LLMs, and it showed stability between rounds 2 and 3. The participants agreed on 7 potential benefits of using LLM-based systems in health care (Table 3): “More efficient data handling and extraction,” “Improved process automation,” “Improved quality of health services,” “Personalized care,” “Improved health outcomes,” “Faster diagnosis and treatment,” and “Facilitated patient-professional interaction.” Most (13/21, 62%) of the participants felt that these systems will not reduce health care costs.

**Table 3:** Participants’ perceptions on the general benefits of using large language models in health care.

Item	Agreement or disagreement	Scores of 4 or 5 (n=21), n (%)	Scores of 5 (n=21), n (%)	Stability
More efficient data handling and extraction	Agreement	20 (95)	9 (43)	Yes
Improved process automation	Agreement	20 (95)	7 (33)	Yes
Improved quality of health services	Agreement	19 (91)	8 (38)	No
Personalized care	Agreement	19 (91)	4 (19)	Yes
Improved health outcomes	Agreement	17 (81)	4 (19)	Yes
Faster diagnosis and treatment	Agreement	17 (81)	3 (14)	Yes
Facilitated patient-professional interaction	Agreement	18 (76)	6 (29)	Yes
Improved clinical communication	Agreement	15 (71)	2 (10)	Yes
Increased caregiver empowerment	Agreement	15 (71)	1 (5)	Yes
Reduced workload for health care professionals	Disagreement	15 (71)	6 (29)	Yes
Resource optimization	Agreement	15 (71)	4 (19)	No
Reduction of human errors	Agreement	15 (71)	1 (5)	Yes
Improved interoperability	Agreement	14 (67)	2 (10)	N/A^a^
Reduced health care costs	Agreement	8 (38)	1 (5)	Yes

^a^N/A: not applicable.

### Shortcomings and Risks of LLM-Based Systems in Health Care

The shortcomings of using LLM-based systems in health care were grouped into 5 dimensions (Table 4): “Risks to health care,” “Risks to the medical profession,” “Risks to patients,” “Risks related to data protection,” and “Risks to the health IT field.” Agreement was reached in almost two-thirds (29/50, 58%) of the evaluated items. All items except the “accessibility issues” item showed stability in round 3.

**Table 4:** Shortcomings and risks of large language model (LLM)–based systems in health care.

Item			Agreement or disagreement		Scores of 4 or 5 (n=21), n (%)		Scores of 5 (n=21), n (%)		Stability
**Risks to health care**									
	Cybersecurity risks	Agreement		18 (86)		10 (48)		Yes	
	Risk of misinformation of patients	Agreement		18 (86)		9 (43)		Yes	
	Ethical risks	Agreement		17 (81)		14 (67)		Yes	
	Risk of biased decisions	Agreement		17 (81)		6 (29)		Yes	
	Risk of inaccurate communication	Agreement		16 (76)		7 (33)		Yes	
	Lack of explainability of system decision-making processes	Disagreement		15 (71)		9 (43)		Yes	
	Risk of increasing health inequities	Disagreement		14 (67)		6 (29)		Yes	
	Limited interoperability of generated outputs	Agreement		13 (62)		4 (19)		Yes	
	Risk of dehumanization of care	Disagreement		13 (62)		2 (10)		Yes	
	Risk of errors (HCPs^a^)	Agreement		12 (57)		3 (14)		Yes	
	Negative clinical outcomes	Disagreement		12 (57)		4 (19)		Yes	
	Risk of information overload of patients	Disagreement		10 (48)		3 (14)		Yes	
	Risk of information overload of HCPs	Disagreement		9 (43)		3 (14)		Yes	
**Risks to the medical profession**									
	Overconfidence in LLM-based models	Agreement		16 (76)		5 (24)		Yes	
	Impact on jobs in the health care sector	Disagreement		15 (71)		4 (19)		Yes	
	Misdiagnosis due to wrong generated results	Agreement		14 (67)		3 (14)		Yes	
	Liability for errors made by LLM-based systems	Agreement		13 (62)		4 (19)		Yes	
	Lack of understanding of the underlying technology	Disagreement		13 (62)		2 (10)		Yes	
	Risk of losing knowledge and competencies	Disagreement		9 (43)		2 (10)		Yes	
	Risk of attempts to replace health care practitioners with tools	Disagreement		8 (38)		2 (10)		Yes	
	Loss of communication skills	Disagreement		6 (29)		2 (10)		Yes	
	Loss of trust of patients in HCPs	Disagreement		6 (29)		1 (5)		Yes	
	Reduced need for medical professionals	Disagreement		2 (10)		0 (0)		Yes	
**Risks to patients**									
	Risk of inaccurate communication	Agreement		15 (71)		2 (10)		Yes	
	Lack of transparency of system use	Disagreement		13 (62)		3 (14)		Yes	
	Wrong personal health decisions due to the use of unverified information	Agreement		13 (62)		1 (5)		Yes	
	Accessibility issues	Disagreement		11 (52)		4 (19)		No	
	Loss of patient-professional contact	Disagreement		11 (52)		3 (14)		Yes	
	Incorrect treatment plans	Disagreement		11 (52)		1 (5)		Yes	
	Incorrect diagnoses	Disagreement		10 (48)		2 (10)		Yes	
	Loss of trust in HCPs	Disagreement		6 (29)		1 (5)		Yes	
**Risks related to data protection**									
	Use of unregulated cloud services may risk data security and privacy	Agreement		18 (86)		7 (33)		Yes	
	Disclosure of sensitive patient data during training and inference	Agreement		18 (86)		5 (24)		Yes	
	Breach of patient confidentiality	Agreement		17 (81)		4 (19)		Yes	
	Fraudulent use of information	Agreement		16 (76)		6 (29)		Yes	
	Vulnerabilities in data storage systems or communication channels	Agreement		16 (76)		5 (24)		Yes	
	Risk that individual patient data may be accessed or used inappropriately	Agreement		16 (76)		5 (24)		Yes	
	Breach of GDPR^b^	Agreement		15 (71)		4 (19)		Yes	
	Risk of patient reidentification	Agreement		15 (71)		4 (19)		Yes	
	Uncontrolled access by third parties	Disagreement		14 (67)		8 (38)		Yes	
**Risks to the health IT field**									
	Unresolved responsibilities for system errors or wrong outputs hamper adoption of LLM-based systems	Agreement		18 (86)		6 (29)		Yes	
	Developing and delivering solutions compliant with regulations is complex for health IT companies	Agreement		17 (81)		5 (24)		Yes	
	Competitive pressure leads to market release of LLM-based systems of low quality	Agreement		17 (81)		5 (24)		Yes	
	Lack of understanding of clinical risks leads to systems that can harm patients	Agreement		16 (76)		4 (19)		Yes	
	Financial constraints at health care institutions for maintenance of LLM-based systems will hamper the adoption of high-quality systems	Agreement		15 (71)		5 (24)		Yes	
	Lack of skilled workers for developing LLM-based systems will hamper the development of high-quality systems	Agreement		15 (71)		3 (14)		Yes	
	A missing standard quality assessment framework for LLM-based systems will lead to low-quality systems released to market	Agreement		14 (67)		5 (24)		Yes	
	LLM-based systems will lack integration into clinical systems	Agreement		14 (66.7)		1 (5)		Yes	
	Missing reimbursement models for LLM-based systems hamper the adoption of technology	Disagreement		10 (48)		6 (29)		Yes	
	Companies’ lack of competence to ensure the development of systems compliant with regulations	Agreement		10 (48)		2 (10)		Yes	

^a^HCP: health care professional.

^b^GDPR: General Data Protection Regulation.

Regarding the risks to health care, agreement was reached on 54% (7/13) of the evaluated items. The experts agreed that the use of LLM-based systems can lead to 5 of the evaluated risks: “Cybersecurity risks,” “Risk of misinformation of patients,” “Ethical risks,” “Risks of biased decisions,” and “Risks of inaccurate communication.” On the other hand, the experts did not believe that the use of these systems could provoke information overload in health care professionals (HCPs) or patients.

Agreement was reached on 30% (3/10) of the evaluated risks to the medical profession. Only 1 of the evaluated risks to the medical profession, “Overconfidence in LLM-based systems,” was recognized by most of the experts. The experts did not believe that the use of these systems will lead to a reduced need for medical professionals.

Agreement was reached on 25% (2/8) of the risks to patients (“Risk of inaccurate communication” and “Wrong personal health decisions due to the use of unverified information”). None of the risks were scored with a 4 or 5 by >75% of the experts.

Regarding the risks related to data protection, only in 1 item, “Uncontrolled access by third parties,” agreement was not reached. The experts believed that LLM-based systems could lead to 6 risks related to data protection: “Use of unregulated cloud services may risk data security and privacy,” “Disclosure of sensitive patient data during training and inference,” “Breach of patient confidentiality,” “Fraudulent use of information,” “Vulnerabilities in data storage systems or communication channels,” and “Risk of individual patient data may be accessed or used inappropriately.”

Agreement was not reached in only 10% (1/10) of the risks to the health IT field. The experts agreed that LLM-based systems can lead to 4 risks in this dimension: “Unresolved responsibilities for system error or wrong outputs hamper adoption of LLM-based systems,” “Developing and delivering solutions compliant with regulations is complex for health IT companies,” “Competitive pressure leads to market release of LLM-based systems of low quality,” and “Lack of understanding of clinical risks leads to systems that can harm patients.”

### Needs for Future Adoption and Implementation of High-Quality LLM-Based Systems

In all items related to future adoption and implementation of high-quality LLM-based systems included in this section, agreement and stability were reached in round 3 (Table 5). All items were considered relevant by most of the experts. All experts agreed that successful adoption of LLM-based systems in practice requires training of HCPs and quality assessment standards.

**Table 5:** Experts’ agreement on requirements for the successful adoption of large language model (LLM)–based systems in the health care domain.

Item	Agreement or disagreement	Scores of 4 or 5 (n=21), n (%)	Scores of 5 (n=21), n (%)	Stability
Successful adoption in practice requires training of HCPs^a^	Agreement	21 (100)	15 (71)	Yes
Successful adoption in practice requires quality assessment standards	Agreement	21 (100)	15 (71)	Yes
Successful adoption in practice requires regulations on data privacy for such systems	Agreement	20 (95)	15 (71)	Yes
Successful adoption in practice requires proper standards for data security and data privacy	Agreement	20 (95)	16 (76)	Yes
Successful adoption in practice requires algorithm or vigilance	Agreement	20 (95)	14 (67)	Yes
Successful adoption in practice requires training of health IT personnel	Agreement	20 (95.2)	12 (57)	Yes
Successful adoption of LLM-based systems supporting the decision-making process in practice requires co-design of new workflows with HCPs	Agreement	20 (95)	11 (52)	Yes
Successful adoption in practice requires regulations on data ownership	Agreement	18 (86)	14 (67)	Yes
Successful adoption in practice requires guidelines for interpretation of the results of LLM-based systems and their use in clinical practice	Agreement	18 (86)	14 (67)	Yes
Successful adoption in practice requires a cultural change in health care	Agreement	17 (81)	12 (57)	No
Successful adoption in practice requires integration with existing EHRs^b^ if the LLM-based system is to support the decision-making process	Agreement	17 (81)	11 (52)	Yes
Successful adoption in practice requires the adaptation of jobs in the health care domain	Agreement	17 (81)	6 (29)	Yes
Successful adoption in practice requires reimbursement models for LLM-based systems and their use in health care	Agreement	16 (76)	5 (24)	Yes

^a^HCP: health care professional.

^b^EHR: electronic health record.

### Reliability of Systems Based on LLMs

Agreement was reached on all the characteristics evaluated regarding reliability, and they were considered relevant by the experts (Table 6). All participants agreed on half of the characteristics: “The system is tested in real settings,” “The system outputs are reproducible,” “The system outputs are reliable,” “The system is robust against a wide range of inputs,” “Quality of the data underlying the system is ensured,” “The system is tested in simulated settings with real users,” and “The system is validated for accuracy.”

**Table 6:** Requirements for reliable systems based on large language models (LLMs).

Item	Agreement or disagreement	Scores of 4 or 5 (n=21), n (%)	Scores of 5 (n=21), n (%)	Stability
The system is tested in real settings	Agreement	21 (100)	17 (81)	Yes
The system outputs are reproducible	Agreement	21 (100)	15 (71)	Yes
The system outputs are reliable	Agreement	21 (100)	14 (67)	Yes
The system is robust against a wide range of inputs	Agreement	21 (100)	13 (62)	Yes
Quality of the data underlying the system is ensured	Agreement	21 (100)	12 (57)	Yes
The system is tested in simulated settings with real users	Agreement	21 (100)	11 (52)	Yes
The system is validated for accuracy	Agreement	21 (100)	10 (48)	Yes
The system meets federal regulations	Agreement	20 (95)	15 (71)	Yes
The system is interoperable with existing health care systems	Agreement	20 (95)	11 (52)	Yes
Control mechanisms or human-in-the-loop processes are established to ensure reliability of LLM-based systems	Agreement	19 (91)	11 (52)	Yes
A standardized quality assessment is available for the system	Agreement	19 (91)	11 (52)	Yes
The system has been proven to be noninferior in a variety of clinical settings	Agreement	19 (91)	7 (33)	Yes
Explanations of the reasoning behind model predictions and recommendations are available	Agreement	19 (91)	6 (29)	Yes
The system can solve easy routine tasks with nearly 100% accuracy	Agreement	17 (81)	7 (33)	Yes

### Future of LLMs

All statements on the future of LLMs reached stability in round 3 (Table 7). Agreement was not reached on 3 of these statements. Most of the experts agreed on 5 of the statements: “LLMs will be combined with other technologies in future health applications,” “Applications based on LLMs will be used by HCPs,” “LLMs will have an impact on future technologies in healthcare,” “Applications based on LLMs will be used by patients,” and “LLMs will replace other technologies.” Results from round 3 are shown in Multimedia Appendix 5 in more detail.

**Table 7:** Experts’ opinions on statements related to the future of large language model (LLM)–based systems in health care.

Item	Agreement or disagreement	Scores of 4 or 5 (n=21), n (%)	Scores of 5 (n=21), n (%)	Stability
LLMs will be combined with other technologies in future health applications	Agreement	21 (100)	10 (48)	Yes
Applications based on LLMs will be used by health care professionals	Agreement	21 (100)	2 (10)	Yes
LLMs will have an impact on future technologies in health care	Agreement	20 (95)	5 (24)	Yes
Applications based on LLMs will be used by patients	Agreement	18 (86)	7 (33)	Yes
LLMs will replace other technologies	Agreement	18 (86)	2 (10)	Yes
The medical device regulation hampers the introduction of solutions based on LLMs	Disagreement	14 (67)	1 (5)	Yes
Solutions based on LLMs will help address the shortage of skilled health professionals	Agreement	14 (67)	0 (0)	Yes
To what extent will future health care rely on LLM-based solutions?	Agreement	12 (57)	0 (0)	Yes
LLM-based solutions will contribute to discrimination in health care because they rely on biased data	Agreement	11 (52)	1 (5)	Yes
I consider LLMs, specifically their resource consumption, ecologically sustainable	Disagreement	7 (33)	1 (5)	Yes
Students of medicine will lose competencies through the increased use of LLMs	Disagreement	7 (33)	1 (5)	Yes
The introduction of LLMs in digital health solutions will result in cost savings in the health sector	Agreement	6 (29)	1 (5)	Yes
LLMs will be replaced by other technologies in the coming 5 years	Agreement	6 (29)	0 (0)	Yes
Health care professionals (physicians and nurses) will lose competencies through the increased use of LLMs	Agreement	5 (24)	1 (5)	Yes
Solutions based on LLMs will offend the sensibilities of health care professionals	Agreement	5 (24)	0 (0)	Yes
Patients will lose competencies through the increased use of LLMs	Agreement	4 (19)	0 (0)	Yes
Solutions based on LLMs will offend the sensibilities of patients	Agreement	2 (10)	0 (0)	Yes
Solutions based on LLMs will offend the sensibilities of other people involved in the care process	Agreement	2 (10)	0 (0)	Yes

## Discussion

### Principal Findings

Our study describes the views of sector experts on the future potential benefits and risks of LLMs in health care as well as requirements for adoption. The results show that LLMs are expected to be used within virtual health assistants, for helping with various tasks, and for supporting patient education. Key benefits identified included improved data handling, process automation, service quality, personalized care, and faster diagnosis. However, experts also warned of potential risks, such as cybersecurity threats, misinformation, ethical concerns, and decision bias. Key privacy risks included potential breaches of confidentiality and data storage vulnerabilities. This study also highlights the complexity of regulatory compliance and the risk of low-quality system releases due to market pressures. To effectively integrate LLM-based systems into health care, there is a consensus on the need to train HCPs and set quality standards to ensure a balanced approach between reaping the benefits and managing the risks.

In the following sections, we consider the points on which there was agreement in answering the questions that drove our SWOT analysis of LLM-based systems and contextualize the findings with previous work. Implications for practitioners as well as researchers are presented.

### Comparison to Prior Work

Our study describes many use cases and tasks in which systems based on LLMs can be used to support HCPs and patients. These fall into 3 groups: support in clinical tasks, support in documentation tasks, and support in medical research and education.

#### Support in Clinical Tasks

Clinical tasks concern patient-professional interaction. In this context, virtual assistants based on LLMs could be useful in several cases, such as providing medical assistance and information to patients, answering their questions, or conducting patient education. This is in line with the research published on intelligent agents and systems with conversational user interfaces. Although many of the existing agents described are rule based [38], reports on LLM-based systems are emerging [39]. Other clinical tasks with which LLMs could help include triage and diagnostic tasks as well as risk assessment and treatment plan generation. As exemplified, LLMs can be used for predicting the occurrence of chronic diseases in a patient based on information from clinical notes [40].

#### Support in Documentation Tasks

Documentation tasks that experts expect to be supported in the future by LLM-based systems include automatic clinical coding, summarization of clinical documents, automatic structuring of clinical narratives, and medical charting assistance. Again, there is a nascent literature describing these uses. Yang et al [12] presented an LLM-based approach to clinical concept extraction. López-García et al [41] analyzed model performance of LLMs for automatic clinical coding in Spanish. Lentzen et al [5] studied LLM accuracy for automatic structuring of clinical notes in German. LLMs have demonstrated significant performance gains for medical problem summarization tasks [42]. We note preliminary reports of the use of LLMs to generate radiological reports from images [43]. These examples demonstrate the potential for use cases for medical charting assistance.

#### Support in Medical Research and Education

LLMs could support literature review and research, clinical trial matching, guideline generation, and educational resource development. Tian et al [44] successfully tested LLM-based system named entity recognition for parsing clinical trial eligibility criteria.

#### Other Strengths

Experts agreed that LLMs can lead to improved quality of care, better health outcomes, optimized clinical communication, reduced human error, personalized care, and increased caregiver empowerment. These use cases have preliminary evidence in the literature. However, the benefits are still hypothetical, as LLM-based systems have not yet been implemented in daily practice solutions. It remains to be proven whether LLM-based systems can significantly improve digitized processes in health care or reduce costs.

There are potential risks with the use of LLMs to health care in general, to the medical profession, to patients, and to privacy rights.

#### Risks to the Medical Profession

A potential risk to the medical profession is overreliance on automated systems. HCPs may place too much trust in the results generated by LLMs, leading to potential complacency and reliance on technology over clinical judgment [45]. Of concern is the potential for misdiagnosis due to incorrect results produced by LLMs if results are not carefully validated and cross-checked by HCPs.

Human factors may lead to errors such as incorrect use or interpretation of LLM outputs by HCPs. There is a risk that HCPs may make mistakes when relying on information generated by an LLM-based system, potentially affecting patient care and safety. This raises the issue of liability in the event of system failure, which can become a complex legal issue [46]. Possible implications and unintended consequences of LLM-based systems must be considered now before systems are used in practice.

In addition, increased reliance on LLM-based systems could lead to a loss of skills among HCPs. As they become more accustomed to using automated outputs, they may rely less on their own knowledge and skills, which could affect their clinical decision-making. Reliance on LLM-based systems during medical education could result in trainees never acquiring knowledge gained by previous trainee cohorts. Finally, the introduction of LLM-based systems in health care could potentially offend the sensibilities of some HCPs. Some professionals may feel uncomfortable or threatened by the move toward greater automation as it may seem to devalue the human aspect of health care.

#### Risks to Patients

One significant risk to patients is the potential for inaccurate information sharing with them. LLMs may produce outputs that are difficult for patients to understand, leading to misunderstandings or incomplete information exchange during critical health care interactions. Another important concern is the risk of people making poor personal health decisions based on unverified or inaccurate information from LLM outputs. People may misinterpret the information or act on it without proper validation, which may result in making decisions that do not align with their goals for care, preferences, or values. In addition, the introduction of LLMs in health care could offend the sensibilities of some patients. They may feel uncomfortable or uneasy with the idea of their diagnosis or treatment decisions relying on automated technology, potentially leading to a sense of detachment, a perceived lack of personalized care, or disengagement with the health care system. Patients in health care systems that require significant patient cost sharing may be at risk of higher copayments if the costs of implementing transforming technologies are passed on.

#### Risks Related to Data Protection

Cloud services are often used for the training of LLMs. The use of unregulated cloud services introduces potential vulnerabilities, with data security and privacy at risk. During the training and inference processes, sensitive patient data may be inadvertently exposed, possibly compromising patient confidentiality. One of the most significant threats is the potential for fraudulent or illegal use of patient information, which could lead to patient reidentification. If patient data are not adequately protected, identity theft, denial of care, or other malicious activities may result.

Vulnerabilities in data storage systems or communication channels increase the risk of data breaches [47]. Cyberattacks or data leaks through insecure channels could compromise patient information, particularly in light of increasing system complexity and increasing number of features. If not adequately protected following international standards such as the International Organization for Standardization and International Electrotechnical Commission 27000 series, patient data may be accessed or used for purposes other than the intended medical care and clinic operations. In addition, the implementation of LLMs in health care must comply with data protection regulations such as the General Data Protection Regulation. Exploiting vulnerabilities could also threaten patient safety. In this context, some LLM-based applications can be considered safety-critical systems because they store and process information that is needed for patient care (eg, medication data) [47].

#### General Risks

Ethical considerations are another area of concern as LLMs could inadvertently lead to biased decisions based on inadequately representative training data. This could lead to unfair or discriminatory outcomes for some patient populations. The reliance of LLMs on biased data could perpetuate and amplify existing inequalities in health care [48,49]. Furthermore, the implementation of LLMs in health care settings may raise ethical dilemmas and offend certain individuals involved in the care process. Greco et al [50] claimed that there is still only “little discussion...provided to avoid or mitigate the bias of transformed-based models, in terms of different aspects of the users/patients, such as culture, age and gender.”

#### Reliability of Systems

We identified a number of issues relevant to LLM-based systems’ reliability. First, careful, standardized quality testing is essential. LLM-based systems should be shown to be noninferior in a variety of clinical settings. First attempts regarding reporting guidelines for AI-based clinical decision support systems have been made [51]. Second, LLM-based systems should produce reproducible and reliable outputs and be robust to a wide range of inputs. Third, control and reasoning mechanisms need to be established for LLM-based systems’ implementation in practice to ensure reliability and explainability. This point was also confirmed by Greco et al [50] in their survey on LLMs for mental health issues. Kelly et al [52] found that for some scenarios, AI cannot replace or replicate human contact. Fourth, the quality of the underlying data must be ensured. Finally, LLM-based systems must comply with federal regulations.

In summary, there are several disadvantages or challenges of applying LLMs in health care. Reliability of the systems in the real world still has to be proven.

#### Opportunities Contributing LLM Use in Practice

Our panel of experts identified several opportunities that could contribute to the successful implementation of LLMs in health care. LLM-based solutions could free up capacity, mitigating the effects of the current shortage of skilled HCPs. Our experts agreed that LLMs have the potential to increase the efficiency of clinical processes and improve quality. Clinical processes can be optimized through more efficient data handling and extraction using LLMs, process automation, resource optimization, and improved interoperability. LLMs can help make faster diagnoses and reduce time needed for patient education.

Work on standardized data exchange (eg, using Health Level 7 and Fast Healthcare Interoperability Resources) is progressing in health care, which will enable the integration of LLM outputs into existing health IT systems. The experts agreed that LLMs will be combined with other technologies in future health care applications and may therefore influence future health care technologies. Furthermore, some other technologies might be replaced by LLM-based solutions.

#### Threats to Adoption of LLM in Practice

One set of threats is related to IT companies that develop products integrating LLMs. A lack of understanding of clinical risks potentially caused by LLMs may result in systems that have the potential to harm patients. A shortage of system development skills could hinder the development of high-quality systems. Furthermore, companies may lack the expertise and competence required to ensure the development of LLM-based systems that are fully compliant with health care regulations and that function as intended. These regulatory challenges could be barriers to the successful implementation of LLMs in health care settings.

Kokol et al [53] have previously raised concerns about the quality of digital health solutions because “neither the volume, distribution nor scope of the quality research content related to digital health solutions is satisfactory.” They assert that there is a risk of reducing the quality of care due to subpar software and software-based tools.

Another set of threats is related to health care institutions, workflow integration, and maintenance of LLM-based systems. Financial constraints may hinder the adoption of high-quality LLM-based systems as maintenance costs may become prohibitive. A similar observation was made by Sezgin et al [54] in their work on using GPT-3 in the US health care system. Accordingly, there is a risk that LLM-based systems may not be properly integrated with existing clinical systems, leading to inefficiencies, suboptimal performance, and perhaps harm to patients.

We identified several factors that should be considered for a successful adoption of LLM-based systems in health care. At a minimum, HCPs and health IT personnel must be trained appropriately to use LLM-based systems. Without appropriate, adequate, and sufficient training, LLM implementation teams and the HCPs they serve cannot hope to use LLM-based systems.

A second aspect is quality and data security. Quality assessment standards are needed for LLM solutions, and data privacy and ownership regulations need to be considered or developed when none exist. Also required are appropriate standards for data security and privacy [55]. Monitoring the output of algorithms through algorithm or vigilance [56] is essential to ensure patient safety. Unresolved accountability for system failures or incorrect outcomes may hinder the widespread adoption of these technologies. Langlais et al [45] considered AI applications in cardiology and called for a framework for accountability in cases of system error.

A third factor is integration into the clinical workflow. Integration with existing health IT systems, including electronic health records, is a complex and unavoidable challenge for a successful LLM implementation. Otherwise, seamless communication and coordination may be hindered. A culture change in health care and reimbursement models are needed to ensure success. The Digital Health Validitron offered by researchers of the University of Melbourne is one of the first attempts to ensure that digital health solutions are successfully designed, developed, validated, and evaluated [57]. It enables secure co-design, testing, and refinement of ideas for digital health solutions in a laboratory environment in preparation for implementation. Such a platform could also be useful in the current stage of evolving LLM-based solutions in health care.

### Strengths and Limitations of This Study

Although this Delphi study involved experienced participants, the panel was not widely representative. We acknowledged participation in the Delphi panel by offering coauthorship as a group. This could have attracted participation of persons who are not very experienced with LLMs. Furthermore, participants were mainly from computer science, health informatics, and medicine, and almost all (20/21, 95%) worked in academia. Most participants (15/21, 71%) were from Europe, with only one-third from other regions (ie, Australia or Oceania 3/21, 14% in round 3 and North America 4/21, 19% in round 3). European countries have national health care systems and strong privacy regulations for the management of citizens’ personal data. Responses from participants from regions under other circumstances could be rendered insignificant due to the demographic mixture of the respondents. Regulatory, economic, and infrastructural concerns affecting LLM adoption and use may not be reflected. The participants may have had expertise in health informatics or related fields, but only one-third of participants (7/21, 33%) had experience on LLMs limited to testing ChatGPT without having a comprehensive knowledge of LLMs. This could of course affect the reliability of the judgments. The selection of the participants was biased in that we contacted persons on an individual basis based on their publication record and from an industry working group.

The number of participants decreased over 3 rounds but remained within the 75% requirement for participation—stability was not achieved for all items even though their number was small. Future research should investigate whether the items that did not show stability could do so in research involving more rounds or a different group of experts. The design of our study (answer collection only through web-based surveys) meant that participants had no interaction or discussions. Such interactions might have been useful only to clarify the formulations and harmonize the viewpoints. Although the final panel comprised 21 participants, it is not possible to affirm the completeness of the SWOT analysis. We might have missed aspects or aggregated them with other items when they should have been considered separately. In addition, a significant presence of patients or citizens who regularly interact with the health care system might yield different perspectives.

In round 1, we collected a large number of use cases for LLMs. We removed some of them from subsequent rounds as they were not specific to health care. For example, one expert suggested “Building of socio-sanitary services: processing healthcare data with social data (from e.g., the city hall, the public water/gas companies or the treasury department, among others) could be helpful to address specific needs of population at-risk.” Integrating health care data and citizen science is clearly interesting. However, we focused rather on clinical applications. In this way, we might have excluded interesting and relevant cases.

Some participants also commented in their free textual comments in rounds 2 and 3 that their judgments depended on whether LLMs are used to replace HCPs or assist them. Our statements were not formulated clearly enough to capture this distinction and might have led to misunderstanding or bias.

The items on which the participants disagreed over 3 rounds are related to five topics: (1) impact on jobs (“Reduced workload for healthcare professionals,” “Impact on jobs in the healthcare sector,” “Risk of attempts to replace healthcare practitioners,” and “Reduced need for medical professionals”), (2) patient-physician relationship (“Risk of dehumanization of care,” “Risk of information overload of patients,” “Risk of information overload of HCPs,” “Loss of trust of patients in HCPs,” “Loss of patient-professional contact,” and “Loss of trust in HCPs”), (3) quality and transparency of results (“Lack of explainability of system,” “decision making processes,” “Negative clinical outcomes,” “Lack of transparency of use,” “Incorrect treatment plans,” and “Incorrect diagnoses”), (4) accessibility and equity (“Risk of increasing health inequities” and “Accessibility issues”), and (5) skills (“Lack of understanding of the underlying technology,” “Risk of losing knowledge and competencies,” and “Loss of communication skills”).

The disagreements could be related to the different viewpoints and experiences of the participants or a different understanding of how transformers will be integrated into health care processes. For example, the impact on jobs in the health care sector might be judged differently when we envision scenarios in which transformers are used with or without a human in the loop. In addition, personal fears of potentially losing their job could be relevant when judging the items related to future jobs. Variations in how transformer-based technologies are regulated in participants’ countries may have affected their experiences with transformers at the time of the study, thereby influencing their opinions and expectations of the technology.

### Practical Implications

On the basis of our findings, we describe key implications for practitioners. These are intended to create awareness regarding the successful and efficient adoption, development, and deployment of LLMs in the health care ecosystem and to optimally benefit from their existing advantages for practice.

#### Clinical Deployment and Training

Overall, the effectiveness of LLMs in clinical settings relies on the skills and familiarity of the HCPs using LLMs. In this context, it is necessary to improve HCPs’ skills to ensure proper and successful integration into (existing) clinical workflows (eg, for supporting clinical documentation). Moreover, we recommend designing and implementing LLM-based applications in human-in-the-loop processes (ie, involving HCPs in the development process in a collaborative feedback loop), especially for validation tasks. In this way, HCPs will become familiar with the technology more quickly and build the necessary trust in it and its decision-making process.

#### Quality Assessment

The development of standardized quality assessment frameworks for LLM-based systems is essential to ensure the release and adoption only of systems that have achieved a minimum standard of quality. Thus, regulators in collaboration with HCPs should establish guidelines, standards, and benchmarks ensuring patient safety, secure data processing and storing, and privacy while supporting the technology’s innovation potential, and developers should build products that meet these standards. Developers should not only validate the accuracy of the results with HCP oversight but also perform simulations under real conditions, including configurations of the underlying systems. Testing of such applications in diverse scenarios is required for successful implementation and deployment, especially to address regulatory concerns and build HCP and patient trust. LLM-based systems are expected to be able to complete routine tasks in some cases with nearly 100% accuracy.

#### Data Security and Privacy

It is crucial to achieve compliance with well-established data protection regulations such as the General Data Protection Regulation, the Health Insurance Portability and Accountability Act, Genetic Information Nondiscrimination Act, and data security standards such as the International Organization for Standardization and International Electrotechnical Commission 27000 series to ensure secure LLM-based applications. This is critical in health care to avoid increased risks to patients, including but not limited to physical, psychological, legal, economic, and reputational harm as well as discrimination and loss of access to health care services.

In addition, there are multiple potential threats and risks associated with the use of third-party services (eg, cloud services) in LLM training. Consequently, reliable and robust data security measures as well as comprehensive security policies are needed to address the security goals of confidentiality, integrity, and secure availability. Depending on the application, the 3 goals of information security—authorization, accountability, and nonrepudiation—should also be met.

#### Ethical Considerations

Biases and potentially discriminatory outcomes, which are typically not only based on biased training data but also influenced by human perceptions and activities, pose a threat to health care if not anticipated and avoided. Ethical guidelines need to be developed to mitigate these risks, particularly in the context of reliable and ethical outcomes. To address these and other risks related to LLMs, Porsdam Mann et al [58] promoted transparency and engagement in open discussions that will allow LLM developers to demonstrate their commitment to and practice of responsible and ethical practices.

### Research Implications

Our Delphi process articulated a number of future research considerations.

#### Clinical Deployment and Workflow Integration

Clinical workflows can be highly complex, making the successful integration of LLMs quite challenging. Interdisciplinary research approaches are needed to address this concern. Existing workflows can be extended, or new workflows can be created that encompass a user-centered, efficiency-oriented design of LLM-based applications. In each case, real-world evaluations are needed to investigate their actual efficiency and reliability and any potential hurdles such as their functional acceptance by HCPs and patients. The long-term impact of integrating LLMs into clinical workflows should be measured in the context of HCP skills, patient engagement and satisfaction, and overall health care and process quality. It will be important to identify the appropriate outcomes to measure and determine how best to assess them both immediately after implementation and throughout the product life span.

#### Quality Improvement

One of the major issues of LLMs is biased data and outcomes. Techniques that can ensure debiased and equal training are needed. Moreover, we highlight the need for controlled environments for validation and refinement purposes, such as the Digital Health Validitron. Similar platforms and frameworks could effectively facilitate the secure development and evaluation of LLMs for health care. Note that the real-world operational quality of LLMs should be evaluated in different clinical settings and patient populations to successfully test possible application configurations as well as user perceptions. Furthermore, cost-benefit analyses based on comprehensive evaluations including economic impacts are also necessary in this context. In summary, the impact of LLMs on cost savings and environmental sustainability needs further evaluation.

#### Ensuring Explainability

Due to the increasing spread and use of AI applications, it is important to ensure transparent, accurate, and interpretable explanation outcomes. This requirement also applies to LLMs even more in health care given the need to ensure patient safety. By making the entire decision-making process interpretable, concerns related to so-called black box AI can be addressed. Thus, we emphasize the need for techniques or even frameworks to achieve explainable LLMs to improve trust but also HCPs’ and patients’ understanding.

### Conclusions

In this paper, we reported expert agreed opinions regarding the SWOT of LLM-based systems in health care. Many use cases we collected have yet to come to fruition. However, it is a work in progress in terms of research and development of LLMs for many tasks in the health care domain. There are substantial threats to the successful implementation of LLMs, which include the quality and quality assessment, regulatory aspects, and integration with workflows. Research in these areas could contribute to the acceptance in real-world settings and reliability of LLM-based products in health care. We conclude by recommending that research should not only focus on testing the possibilities of LLMs for natural language–related tasks but also consider the workflows that these models could contribute to and the requirements regarding quality, integration, and regulations that are necessary for a successful implementation in practice. With this approach, it will be possible to generate a real impact in health care.

## Appendix

Multimedia Appendix 1 Items of the first-round questionnaire.

Multimedia Appendix 2 Summary of Conducting and Reporting Delphi Studies recommendations.

Multimedia Appendix 3 New items and rewording of the round-2 and round-3 questionnaires.

Multimedia Appendix 4 Evolution of agreement across rounds.

Multimedia Appendix 5 Percentage of participants assigning each score in round 3.

## Abbreviations

AI: artificial intelligence
BERT: Bidirectional Encoder Representations From Transformers
HCP: health care professional
LLM: large language model
NLP: natural language processing
SWOT: strengths, weaknesses, opportunities, and threats

## Data Availability

All data generated or analyzed during this study are included in this published article and its supplementary information files.

## References

[1] Wornow M, Xu Y, Thapa R, Patel B, Steinberg E, Fleming S, Pfeffer MA, Fries J, Shah NH (2023). The shaky foundations of large language models and foundation models for electronic health records. NPJ Digit Med.

[2] Yang R, Tan TF, Lu W, Thirunavukarasu AJ, Ting DS, Liu N (2023). Large language models in health care: development, applications, and challenges. Health Care Sci.

[3] Vaswani A, Shazeer N, Parmar N, Uszkoreit J, Jones L, Gomez AN, Kaiser L, Polosukhin I (2017). Attention is all you need. Proceedings of the Proceedings of the 31st International Conference on Neural Information Processing Systems.

[4] Lentzen M, Linden T, Veeranki S, Madan S, Kramer D, Leodolter W, Frohlich H (2023). A transformer-based model trained on large scale claims data for prediction of severe COVID-19 disease progression. IEEE J Biomed Health Inform.

[5] Lentzen M, Madan S, Lage-Rupprecht V, Kühnel L, Fluck J, Jacobs M, Mittermaier M, Witzenrath M, Brunecker P, Hofmann-Apitius M, Weber J, Fröhlich H (2022). Critical assessment of transformer-based AI models for German clinical notes. JAMIA Open.

[6] Gillioz A, Casas J, Mugellini E, Khaled OA (2020). Overview of the transformer-based models for NLP tasks. Proceedings of the 2020 Federated Conference on Computer Science and Information Systems.

[7] Singhal K, Azizi S, Tu T, Mahdavi SS, Wei J, Chung HW, Scales N, Tanwani A, Cole-Lewis H, Pfohl S, Payne P, Seneviratne M, Gamble P, Kelly C, Babiker A, Schärli N, Chowdhery A, Mansfield P, Demner-Fushman D, Agüera Y Arcas B, Webster D, Corrado GS, Matias Y, Chou K, Gottweis J, Tomasev N, Liu Y, Rajkomar A, Barral J, Semturs C, Karthikesalingam A, Natarajan V (2023). Large language models encode clinical knowledge. Nature.

[8] Wang W, Yang Z, Gao Y, Ney H (2021). Transformer-based direct hidden markov model for machine translation. Proceedings of the 59th Annual Meeting of the Association for Computational Linguistics and the 11th International Joint Conference on Natural Language Processing: Student Research Workshop.

[9] Liu Y, Lapata M (2019). Hierarchical transformers for multi-document summarization. Proceedings of the 57th Annual Meeting of the Association for Computational Linguistics.

[10] Li H, Wang AY, Liu Y, Tang D, Lei Z, Li W (2019). An augmented transformer architecture for natural language generation tasks. Proceedings of the International Conference on Data Mining Workshops.

[11] Wang Y, Gu Y, Yin Y, Han Y, Zhang H, Wang S, Li C, Quan D (2023). Multimodal transformer augmented fusion for speech emotion recognition. Front Neurorobot.

[12] Yang X, Bian J, Hogan WR, Wu Y (2020). Clinical concept extraction using transformers. J Am Med Inform Assoc.

[13] Devlin J, Chang MW, Lee K, Toutanova K (2019). BERT: pre-training of deep bidirectional transformers for language understanding. Proceedings of the North American Chapter of the Association for Computational Linguistics: Human Language Technologies.

[14] Chow JC, Sanders L, Li K (2023). Impact of ChatGPT on medical chatbots as a disruptive technology. Front Artif Intell.

[15] Cocci A, Pezzoli M, Lo Re M, Russo GI, Asmundo MG, Fode M, Cacciamani G, Cimino S, Minervini A, Durukan E (2024). Quality of information and appropriateness of ChatGPT outputs for urology patients. Prostate Cancer Prostatic Dis.

[16] Farrokhnia M, Banihashem SK, Noroozi O, Wals A (2023). A SWOT analysis of ChatGPT: implications for educational practice and research. Innov Educ Teach Int.

[17] Garg RK, Urs VL, Agarwal AA, Chaudhary SK, Paliwal V, Kar SK (2023). Exploring the role of ChatGPT in patient care (diagnosis and treatment) and medical research: a systematic review. Health Promot Perspect.

[18] Lee P, Bubeck S, Petro J (2023). Benefits, limits, and risks of GPT-4 as an AI chatbot for medicine. N Engl J Med.

[19] Vedula SS, Ghazi A, Collins JW, Pugh C, Stefanidis D, Meireles O, Hung AJ, Schwaitzberg S, Levy JS, Sachdeva AK (2022). Artificial intelligence methods and artificial intelligence-enabled metrics for surgical education: a multidisciplinary consensus. J Am Coll Surg.

[20] Collins JW, Marcus HJ, Ghazi A, Sridhar A, Hashimoto D, Hager G, Arezzo A, Jannin P, Maier-Hein L, Marz K, Valdastri P, Mori K, Elson D, Giannarou S, Slack M, Hares L, Beaulieu Y, Levy J, Laplante G, Ramadorai A, Jarc A, Andrews B, Garcia P, Neemuchwala H, Andrusaite A, Kimpe T, Hawkes D, Kelly JD, Stoyanov D (2022). Ethical implications of AI in robotic surgical training: a Delphi consensus statement. Eur Urol Focus.

[21] Shinners L, Aggar C, Grace S, Smith S (2021). Exploring healthcare professionals' perceptions of artificial intelligence: validating a questionnaire using the e-Delphi method. Digit Health.

[22] McKenna HP (1994). The Delphi technique: a worthwhile research approach for nursing?. J Adv Nurs.

[23] Hsu C, Sandford B (2007). The Delphi technique: making sense of consensus. Pract Assess Res Eval.

[24] Neely A (1993). Production/operations management: research process and content during the 1980s. Int J Oper Prod Manag.

[25] Meredith JR, Raturi A, Amoako‐Gyampah K, Kaplan B (1989). Alternative research paradigms in operations. J Oper Manag.

[26] Akkermans H, Bogerd P, Vos B (1999). Virtuous and vicious cycles on the road towards international supply chain management. J Econ Stud.

[27] Akkermans HA, Bogerd P, Yücesan E, van Wassenhove LN (2003). The impact of ERP on supply chain management: exploratory findings from a European Delphi study. Eur J Oper Res.

[28] Daniel EM, White A (2017). The future of inter-organisational system linkages: findings of an international Delphi study. Eur J Inf Syst.

[29] Liyanage H, Liaw ST, Jonnagaddala J, Schreiber R, Kuziemsky C, Terry AL, de Lusignan S (2019). Artificial intelligence in primary health care: perceptions, issues, and challenges. Yearb Med Inform.

[30] Lam K, Iqbal FM, Purkayastha S, Kinross JM (2021). Investigating the ethical and data governance issues of artificial intelligence in surgery: protocol for a Delphi study. JMIR Res Protoc.

[31] Boulkedid R, Abdoul H, Loustau M, Sibony O, Alberti C (2011). Using and reporting the Delphi method for selecting healthcare quality indicators: a systematic review. PLoS One.

[32] Humphrey-Murto S, Varpio L, Wood TJ, Gonsalves C, Ufholz LA, Mascioli K, Wang C, Foth T (2017). The use of the Delphi and other consensus group methods in medical education research: a review. Acad Med.

[33] Houben G, Lenie K, Vanhoof K (1999). A knowledge-based SWOT-analysis system as an instrument for strategic planning in small and medium sized enterprises. Decis Support Syst.

[34] Braun V, Clarke V (2006). Using thematic analysis in psychology. Qual Res Psychol.

[35] von der Gracht HA (2012). Consensus measurement in Delphi studies: review and implications for future quality assurance. Technol Forecast Soc Change.

[36] Jünger S, Payne SA, Brine J, Radbruch L, Brearley SG (2017). Guidance on Conducting and REporting DElphi Studies (CREDES) in palliative care: recommendations based on a methodological systematic review. Palliat Med.

[37] McMillan SS, King M, Tully MP (2016). How to use the nominal group and Delphi techniques. Int J Clin Pharm.

[38] Denecke K, May R (2023). Investigating conversational agents in healthcare: application of a technical-oriented taxonomy. Procedia Comput Sci.

[39] Xygi E, Andriopoulos AD, Koutsomitropoulos DA (2023). Question answering chatbots for biomedical research using transformers. Proceedings of the International Conference on Artificial Intelligence in Information and Communication.

[40] Saigaonkar S, Narawade V (2022). Predicting chronic diseases using clinical notes and fine-tuned transformers. Proceedings of the IEEE Bombay Section Signature Conference.

[41] López-García G, Jerez JM, Ribelles N, Alba E, Veredas FJ (2021). Transformers for clinical coding in Spanish. IEEE Access.

[42] Gao Y, Miller T, Xu D, Dligach D, Churpek MM, Afshar M (2022). Summarizing patients' problems from hospital progress notes using pre-trained sequence-to-sequence models. Proc Int Conf Comput Ling.

[43] Nooralahzadeh F, Gonzalez NP, Frauenfelder T, Fujimoto K, Krauthammer M (2021). Progressive transformer-based generation of radiology reports. Proceedings of the Empirical Methods in Natural Language Processing.

[44] Tian S, Erdengasileng A, Yang X, Guo Y, Wu Y, Zhang J, Bian J, He Z (2021). Transformer-based named entity recognition for parsing clinical trial eligibility criteria. Proceedings of the 12th ACM Conference on Bioinformatics, Computational Biology, and Health Informatics.

[45] Langlais É, Thériault-Lauzier P, Marquis-Gravel G, Kulbay M, So DY, Tanguay JF, Ly HQ, Gallo R, Lesage F, Avram R (2023). Novel artificial intelligence applications in cardiology: current landscape, limitations, and the road to real-world applications. J Cardiovasc Transl Res.

[46] Banja JD, Hollstein RD, Bruno MA (2022). When artificial intelligence models surpass physician performance: medical malpractice liability in an era of advanced artificial intelligence. J Am Coll Radiol.

[47] May R, Biermann C, Krüger J, Saake G, Leich T (2022). A systematic mapping study of security concepts for configurable data storages. Proceedings of the 26th ACM International Systems and Software Product Line Conference - Volume A.

[48] Raza S, Osivand Pour P, Bashir SR Fairness in machine learning meets with equity in healthcare. arXiv.

[49] Denecke K, Baudoin CR (2022). A review of artificial intelligence and robotics in transformed health ecosystems. Front Med (Lausanne).

[50] Greco CM, Simeri A, Tagarelli A, Zumpano E (2023). Transformer-based language models for mental health issues: a survey. Pattern Recognit Lett.

[51] Vasey B, Nagendran M, Campbell B, Clifton DA, Collins GS, Denaxas S, Denniston AK, Faes L, Geerts B, Ibrahim M, Liu X, Mateen BA, Mathur P, McCradden MD, Morgan L, Ordish J, Rogers C, Saria S, Ting DS, Watkinson P, Weber W, Wheatstone P, McCulloch P (2022). Reporting guideline for the early stage clinical evaluation of decision support systems driven by artificial intelligence: DECIDE-AI. BMJ.

[52] Kelly S, Kaye SA, Oviedo-Trespalacios O (2023). What factors contribute to the acceptance of artificial intelligence? a systematic review. Telemat Inform.

[53] Kokol P, Vošner HB, Kokol M, Završnik J (2022). The quality of digital health software: should we be concerned?. Digit Health.

[54] Sezgin E, Sirrianni J, Linwood SL (2022). Operationalizing and implementing pretrained, large artificial intelligence linguistic models in the US health care system: outlook of generative pretrained transformer 3 (GPT-3) as a service model. JMIR Med Inform.

[55] Aung YY, Wong DC, Ting DS (2021). The promise of artificial intelligence: a review of the opportunities and challenges of artificial intelligence in healthcare. Br Med Bull.

[56] Embi PJ (2021). Algorithmovigilance-advancing methods to analyze and monitor artificial intelligence-driven health care for effectiveness and equity. JAMA Netw Open.

[57] Capurro D, Huckdale K, Chapman W, Pantano V (2022). The digital health validitron. Australian Healthcare and Hospitals Association.

[58] Porsdam Mann S, Earp BD, Nyholm S, Danaher J, Møller N, Bowman-Smart H, Hatherley J, Koplin J, Plozza M, Rodger D, Treit PV, Renard G, McMillan J, Savulescu J (2023). Generative AI entails a credit–blame asymmetry. Nat Mach Intell.

