# Supplementary material for: Potential of Large Language Models in Health Care: Delphi Study
Source: J Med Internet Res. 2024 May 13;26:e52399. doi: 10.2196/52399 (PMC11130776; doi:10.2196/52399)
Supplement: Multimedia Appendix 1 [file jmir_v26i1e52399_app1.docx]

# **Multimedia Appendix 1: Items of the first round questionnaire**

**Demographics:**

- E-mail;
- Gender (Female / Male / Nonbinary / Prefer not to tell);
- Education/Background (Computer Science/Engineering; Health informatics; Medicine; Nursing; Physiotherapy; Psychology; Other Health Sciences; Sociology; Other);
- Years of working experience (<5 years; 5-10 years; >10 years);
- Sector you are currently working in (Academia; Industry; Public health sector; Private health sector; Other);
- Continent where you are working currently (Africa; Asia; Europe; Australia and Oceania; North America; South America);
- Level of experience regarding LLMs (I am expert. I know and user apply nearly all of their functions; I use their basic functions regularly; I know how they work; I tested ChatGPT, but have only basic knowledge on the underlying technology; I have no knowledge)

**Open-ended questions:**

- Which use cases do you envision that involve LLMs (e.g. in patient-professional interaction, interaction between health professionals)?
- What are the benefits of LLMs in healthcare?
- Which shortcomings of applying LLMs in healthcare do you see?
- Which risks do you see for the medical profession when it comes to an adoption of LLMs in health IT?
- Which risks do you see for patient care when it comes to an adoption of LLMs in health IT?
- Which risks do you see for the field of health IT when it comes to an adoption of LLMs in health IT?
- Which risks do you see related to data protection of healthcare data when it comes to an adoption of LLMs in health IT?
- When would you consider digital solutions based on LLMs be reliable?

**5-Likert questions:**

- To what extent will future healthcare rely upon LLM-based solutions?
- LLMs will be replaced by other technologies in the coming 5 years
- Applications based on LLMs will be used by healthcare professionals
- LLMs will be combined with other technologies in future health applications
- LLMs will replace other technologies
- Solutions based on LLMs will help addressing the shortage of skilled health professionals (e.g. by taking over documentation tasks)?
- LLM-based digital health solutions will contribute to discrimination in healthcare because they rely upon biased data
- The medical device regulation hampers the introduction of solutions based on LLMs
- I consider LLMs, specifically their resource consumption, ecologically sustainable
- Solutions based on LLMs will offend the sensibilities of healthcare professionals
- Solutions based on LLMs will offend the sensibilities of patients
- Solutions based on LLMs will offend the sensibilities of other people involved in the care process
- Healthcare professionals (physicians, nurses) will lose competencies through the increased use of LLMs (e.g. become less competent in writing long texts)
- Students of medicine will lose competencies through the increased use of LLMs (e.g. become less competent in writing long texts)
- Patients will lose competencies through the increased use of LLMs (e.g. become less competent in writing long texts)
- Applications based on LLMs will be used by patients
- LLMs will have an impact on future technologies in healthcare
- The introduction of LLMs in digital health solutions will result in cost savings in the health sector
