# Supplementary material for: Potential of Large Language Models in Health Care: Delphi Study
Source: J Med Internet Res. 2024 May 13;26:e52399. doi: 10.2196/52399 (PMC11130776; doi:10.2196/52399)
Supplement: Multimedia Appendix 2 [file jmir_v26i1e52399_app2.docx]

# **Multimedia Appendix 2: Summary of CREDES reporting recommendations**

| Recommendation | Item # | Explanation | Reported on |
| --- | --- | --- | --- |
| Purpose and rationale | 8 | The purpose of the study should be clearly defined and demonstrate the appropriateness of the use of the Delphi technique as a method to achieve the research aim.  A rationale for the choice of the Delphi technique as the most suitable method needs to be provided | The purpose is reported in section 1. Introduction. Pages X-X  Appropriateness and rationale could be found in section 2.  Methods.  Pages X-X |
| Expert panel | 9 | Criteria for the selection of participants and transparent information on recruitment of the panel, socio-demographic details including information on expertise regarding the topic in question, (non)response and response rates over the ongoing iterations should be reported | Panel information is reported in section 2.6. Recruitment and panel size. Pages X-X  Socio-demographic details and response rates are reported in section 3.1 Characteristics of the panel. Pages X-X |
| Description of methods | 10 | The methods employed need to be comprehensible; this includes information on preparatory steps, piloting of material and survey instruments, design of the survey instrument(s), the number and design of survey rounds, methods of data analysis, processing and synthesis of participants’ responses to inform the subsequent survey round and methodological decisions taken by the research team throughout the process | Preparatory steps are reported in section 2.1 Preparatory phase. Pages X-X.  Piloting, survey design, rounds, and synthesis are reported on section 2. Methods. Pages X-X  Data analysis and team decisions are reported on 2.3 Consensus and stopping criteria and 2.4 Data analysis. Pages X-X |
| Procedure | 11 | Flow chart to illustrate the stages of the Delphi process, including a preparatory phase, the actual ‘Delphi rounds’, interim steps of data processing and analysis, and concluding steps | Flowchart is included in section 2. Methods. Pages X-X |
| Definition and attainment of consensus | 12 | It needs to be comprehensible to the reader how consensus was achieved throughout the process, including strategies to deal with non-consensus | Consensus definition is reported in section 2.3 Consensus and stopping criteria. Pages X-X  Strategies to deal with dissent and consensus are reported in section 2.4. Data analysis. Pages X-X |
| Results | 13 | Reporting of results for each round separately is highly advisable in order to make the evolving of consensus over the rounds transparent. This includes figures showing the average group response, changes between rounds, as well as any modifications of the survey instrument such as deletion, addition or modification of survey items based on previous rounds | Results of round 3 are reported in section 3.3-3.8. Pages X-X  Results of round 1 and round 2 are reported in Appendix IV.  Figures are reported in Appendix V.  Modifications are reported in Appendix II. |
| Discussion of limitations | 14 | Reporting should include a critical reflection of potential limitations and their impact of the resulting guidance | Limitations are reported in section 4.3 Limitations. Pages X-X |
| Adequacy of conclusions | 15 | The conclusions should adequately reflect the outcomes of the Delphi study with a view to the scope and applicability of the resulting practice guidance | Conclusions are reported in section 4.4. Conclusion. Pages X-X |
| Publication and dissemination* | 16 | The main results and discussion . | Discussion is reported in section 4.Discussion. Pages X-X |
