# Supplementary material for: Potential of Large Language Models in Health Care: Delphi Study
Source: J Med Internet Res. 2024 May 13;26:e52399. doi: 10.2196/52399 (PMC11130776; doi:10.2196/52399)
Supplement: Multimedia Appendix 3 [file jmir_v26i1e52399_app3.docx]

# **Multimedia Appendix 3: New items and rewording of the round-2 and round-3 questionnaires**

## III.1 Round 2

After Round 1, just the email address was collected from the experts in the demographic section. As result of the qualitative analysis, 83 new items were added as 5-Likert-statements. These items were grouped into 7 new sections and 11 dimensions. In addition, 5 open-ended questions were included to provide participants with the opportunity to share any comments on the added items or to report any potential factor to be included in subsequent rounds.

**Benefits and shortcomings of LLMs:**

- Please rate the likelihood that the following use cases applying LLMs will result in realistic healthcare scenarios supporting clinical tasks:
  - Support in the diagnostic process (e.g. by analyzing patient data)
  - Patient triage
  - Prediction of risks for disease development
  - Automatic treatment plan generation based on available information
  - Verbalising interactions (factual knowledge comes from humans, but interaction is realized by LLM-based systems)
  - Virtual health assistant for patients to provide medical assistance and information
  - Provision of automatic follow-up in chronic diseases
  - Virtual health assistants for patient education
  - Virtual health assistants for answering patient queries
- Please rate the likelihood that the following use cases applying LLMs will result in realistic healthcare scenarios supporting in documentation tasks:
  - Summarization (e.g. of medical records, clinical conversations)
  - Generation of lay-person summaries from clinical narratives
  - Medical charting assistance
  - Automatic structuring of clinical narrative
  - Automatic clinical encoding
  - Virtual health assistants for administrative tasks
- Please rate the likelihood that the following use cases applying LLMs will result in realistic healthcare scenarios supporting in medical research and education:
  - Literature review and research (eg to support evidence-based medicine)
  - Clinical trial matching (i.e. match patients with appropriate clinical trials)
  - Automatic generation of guidelines based on the evidence available in the published scientific literature
  - Development of educational resources, such as online courses and simulations, to support medical education and training

**Benefits of LLMs:**

- Please indicate your level of agreement that the introduction of LLMs in healthcare applications can lead to the following benefits or improvements:
  - Improved process automatization
  - Reduced workload for healthcare professionals
  - Improved clinical communication
  - Improved quality of health services by reducing the burden of routine tasks
  - Faster diagnosis and treatment
  - More efficient data handling and extraction
  - Improved health outcomes
  - Increased patients' empowerment
  - Resource optimization
  - Personalized care
  - Reduced healthcare costs
  - Facilitated patient-professional interaction (e.g. through explanations or automatic translation to other languages or other cultural levels)
  - Reduction of human errors
  - Increased caregivers empowerment

**Shortcomings and risks of LLMs in healthcare:**

- Please indicate your level of agreement that the introduction of LLMs in healthcare applications can lead to the following risks in healthcare:
  - Limited interpretability of generated outputs
  - Ethical risks (e.g. fairness, transparency, accountability)
  - Lack of explainability of systems decision-making processes
  - Risk of misinformation of patients (e.g. due to hallucinations of the models)
  - Risk of inaccurate communication
  - Risk of increasing health inequities because of lack of competence to use these tools
  - Risk of biased decisions
  - Risk of information overload of patients
  - Risk of information overload of healthcare professionals
  - Risk of errors due to carelessness of health professionals in checking outputs
  - Risk of dehumanization of care
  - Cybersecurity risks
  - Negative clinical outcomes due to improper use
- Please indicate your level of agreement that the introduction of LLMs in healthcare applications can lead to the LLMs in healthcare applications can lead to the following risks for the medical profession:
  - Overconfidence in LLM-based systems
  - Misdiagnosis due to wrong generated results
  - Risk of losing knowledge and competencies (e.g. health professionals may be unable to realize certain tasks on their own when technology is inaccessible or incorrect)
  - Liability for errors made by LLM-based systems
  - Impact on jobs in the healthcare sector (transition to new roles and skills)
  - Loss of communication skills
  - Risk of attempts to replace health practitioners with some tools
  - Loss of trust of patients in healthcare professionals
  - Lack of understanding of the underlying technology
  - Reduced need for medical professionals, originating from automation of tasks
- Please indicate your level of agreement that the introduction of LLMs in healthcare applications can lead to the following risks for patient care:
  - Incorrect treatment plans
  - Wrong personal health decisions due to the use of unverified information
  - Incorrect diagnoses
  - Loss of patient-professional contact
  - Loss of trust in healthcare professionals
  - Lack of transparency of system use
  - Risk of inaccurate communications
  - Accessibility issues

**Risks related to data protection:**

- Please indicate your level of agreement that the introduction of LLMs in healthcare applications can lead to the following risks related to data protection:
  - Use of unregulated cloud services may risk data security and privacy
  - Disclosure of sensitive patient data during training and inference
  - Vulnerabilities in data storage systems or communication channels
  - Uncontrolled access by third parties
  - Breach of GDPR
  - Breach of patient confidentiality
  - Risk of patient reidentification
  - Risk that individual patient data may be accessed or used inappropriately
  - Fraudulent use of information

**Risk for the health IT field:**

- There are several aspects related to health IT that can hamper the introduction of LLMs in healthcare applications. Please indicate your level of agreement:
  - Missing reimbursement models for use of LLM-based systems hampers the adoption of technology
  - Competitive pressure leads to market release of LLM-based systems of low quality (in terms of safety, effectiveness etc.)
  - Unresolved responsibilities for system errors or wrong outputs hamper adoption of LLM-based systems
  - Lack of understanding of clinical risks leads to systems that can harm patients
  - Companies lack of competence to ensure development of systems compliant with regulations
  - LLM-based systems will lack integration into clinical systems
  - Lack of skilled workers for developing LLM-based systems will hamper development of high-quality systems
  - A missing standard quality assessment framework for LLM-based systems will lead to low quality systems released to market
  - Financial constraints at healthcare institutions for maintenance of LLM-based systems will hamper adoption of high-quality systems

**Needs for future adoption and implementation of high-quality LLM-based systems:**

- The successful implementation and adoption of LLM-based systems in healthcare goes along with several needs. Please indicate your level of agreement with the following statements:
  - Successful adoption in practice requires algorithmovigilance (i.e. algorithms' evaluation and monitoring)
  - Successful adoption in practice requires training of health professionals
  - Successful adoption in practice requires training of health IT personnel
  - Successful adoption in practice requires a cultural change in healthcare
  - Successful adoption in practice requires adaptation of jobs in the healthcare domain
  - Successful adoption in practice requires reimbursement models for LLM-based systems and their use in healthcare
  - Successful adoption in practice requires regulations on data privacy for such systems
  - Successful adoption in practice requires regulations on data ownership
  - Successful adoption in practice requires proper standards for data security and data privacy
  - Successful adoption in practice requires quality assessment standards
  - Successful adoption in practice requires guidelines for interpretation of results of LLM-based systems and their use in clinical practice

**Reliability of systems based on LLMs:**

- Please indicate your level of agreement to the following conditions that need to be fulfilled in order to consider LLM-based systems reliable in healthcare:
  - Control mechanisms or human in the loop are established to ensure reliability of LLM-based systems
  - The system is validated for accuracy
  - The system is tested in simulated settings with real users
  - The system is interoperable with existing healthcare systems
  - The system meets federal regulations
  - The system is tested in real settings
  - Explanations on the reasoning behind model predictions and recommendations are available
  - The system outputs are reliable
  - The system outputs are reproducible
  - The system is robust against a wide range of inputs
  - Quality of the data underlying the system is ensured
  - A standardized quality assessment is available for the system
  - The system can solve easy routine tasks with nearly 100% accuracy
  - The system has been proven to be non-inferior in a variety of clinical settings

## III.2 Round 3

The qualitative analysis of the responses of the open-ended questions led to 7 changes in the description of the items:

- "Support in the diagnostic process (e.g., by analyzing patient data)" was changed to "Support in the diagnostic process (e.g., by analyzing or mining patient data)"
- "Prediction of risks for disease development" was changed to "Support in the diagnostic process (e.g., by analyzing patient data)"
- "More efficient data handling and extraction" was changed to "More efficient data handling and extraction (e.g., availability of relevant information at fingertips)"
- "Negative clinical outcomes due to improper use" was changed to "Negative clinical outcomes due to improper use of LLM-based solutions"
- "Misdiagnosis due to wrong generated results" was changed to "Misdiagnosis due to wrong generated results or inaccurate and unreliable medical information"
- "Lack of understanding of the underlying technology" was changed to "Lack of understanding of the underlying technology for a careful use for assistance"
- "Developing solutions compliant with regulations is complex for health IT companies" was changed to "Developing and delivering solutions compliant with regulations is complex for health IT companies"

An item was removed:

- "Increased patients' empowerment"

Finally, 4 new items were added:

- "Design of the chemical compositions of new drugs" was included in the third dimension of section "Benefits and shortcomings of LLMs"
- "Improved interoperability" was included in section "Benefits of LLMs"
- "Successful adoption in practice requires integration with existing EHRs if the LLM-based solution supports the decision-making process" was included in section "Needs for future adoption and implementation of high-quality LLM-based systems"
- "Successful adoption of LLM-based solutions supporting the decision-making process in practice requires co-design of new workflows with healthcare professionals" was included in section "Needs for future adoption and implementation of high-quality LLM-based systems"
