# Supplementary material for: Potential of Large Language Models in Health Care: Delphi Study
Source: J Med Internet Res. 2024 May 13;26:e52399. doi: 10.2196/52399 (PMC11130776; doi:10.2196/52399)
Supplement: Multimedia Appendix 4 [file jmir_v26i1e52399_app4.docx]

# **Multimedia Appendix 4: Evolution of agreements in rounds**

The accumulated results in each round are presented in this appendix.

## IV.1 Perceived likelihood that the use transformer-based systems will support healthcare tasks

| **Item** | **Round 2** | | | **Round 3** | | |
| --- | --- | --- | --- | --- | --- | --- |
|  | **Median** | **IQR** | **% 4 or 5 scores** | **Median** | **IQR** | **% 4 or 5 scores** |
| *Supporting in clinical tasks* | | | | | | |
| Virtual health assistant for patients (education) | 5 | 1 | 91.3 | 4 | 1 | 100 |
| Automatic follow-up in chronic diseases | 4 | 1 | 78.3 | 5 | 1 | 95.2 |
| Virtual health assistant for patients (medical assistance and information) | 4 | 1 | 95.7 | 4 | 1 | 95.2 |
| Prediction of risk for disease development | 4 | 1 | 91.3 | 4 | 1 | 95.2 |
| Virtual health assistant for patients (answering queries) | 5 | 1 | 91.3 | 5 | 1 | 90.5 |
| Diagnostic process | 4 | 1 | 91.3 | 4 | 1 | 90.5 |
| Patient triage | 4 | 1 | 78.3 | 4 | 1 | 85.7 |
| Automatic treatment plan generation | 4 | 1 | 60.9 | 4 | 0.25 | 81.0 |
| Verbalising interactions | 4 | 1.5 | 73.9 | 4 | 0.25 | 76.2 |
| *Documentation tasks* | | | | | |  |
| Automatic clinical encoding | 5 | 1 | 87.0 | 5 | 1 | 95.2 |
| Virtual health assistants for administrative tasks | 4 | 1 | 87.0 | 4 | 1 | 95.2 |
| Summarization | 4 | 1 | 87.0 | 4 | 1 | 90.5 |
| Automatic structuring of clinical narratives | 4 | 1 | 78.3 | 4 | 1 | 90.5 |
| Medical charting assistance | 4 | 1 | 78.3 | 4 | 1 | 90.5 |
| Generation of lay-person summaries | 4 | 0 | 78.3 | 4 | 0 | 81.0 |
| *Medical research and education* | | | | | | |
| Literature review and research | 5 | 1 | 82.6 | 4 | 1 | 90.5 |
| Clinical trial matching | 4 | 1.5 | 73.9 | 4 | 1 | 90.5 |
| Development of educational resources | 4 | 1 | 87.0 | 4 | 1 | 85.7 |
| Automatic generation of guidelines | 4 | 1.5 | 65.2 | 4 | 0 | 76.2 |
| Design of the chemical compositions of new drugs | na | na | na | 4 | 2 | 57.1 |

## IV.2 Benefits of using LLM-based systems in healthcare

| **Item** | **Round 2** | | | **Round 3** | | |
| --- | --- | --- | --- | --- | --- | --- |
|  | **Median** | **IQR** | **% 4 or 5 scores** | **Median** | **IQR** | **% 4 or 5 scores** |
| More efficient data handling and extraction | 4 | 1 | 91.3 | 4 | 1 | 95.2 |
| Improved process automation | 4 | 1 | 95.7 | 4 | 1 | 95.2 |
| Improved quality of health services | 5 | 1 | 95.7 | 4 | 1 | 90.5 |
| Personalized care | 4 | 1 | 73.9 | 4 | 0 | 90.5 |
| Improved health outcomes | 4 | 1 | 69.6 | 4 | 0 | 81.0 |
| Faster diagnosis and treatment | 4 | 0.5 | 73.9 | 4 | 0 | 81.0 |
| Facilitated patient-professional interaction | 4 | 1 | 78.3 | 4 | 1 | 76.2 |
| Improved clinical communication | 4 | 0.5 | 73.9 | 4 | 1 | 71.4 |
| Increased caregivers empowerment | 4 | 1 | 69.6 | 4 | 1 | 71.4 |
| Reduced workload for healthcare professionals | 4 | 1 | 82.6 | 4 | 2 | 71.4 |
| Resource optimization | 4 | 1 | 82.6 | 4 | 1 | 71.4 |
| Reduction of human errors | 4 | 1 | 65.4 | 4 | 1 | 71.4 |
| Improved interoperability | na | na | na | 4 | 1 | 66.7 |
| Reduced healthcare costs | 3 | 1 | 39.1 | 3 | 1 | 38.1 |

## IV.3 Shortcomings and risks of LLM-based systems in healthcare

| **Item** | **Round 2** | | | **Round 3** | | |
| --- | --- | --- | --- | --- | --- | --- |
|  | **Median** | **IQR** | **% 4 or 5 scores** | **Median** | **IQR** | **% 4 or 5 scores** |
| *Risks in healthcare* | | | | | | |
| Cybersecurity risks | 4 | 2 | 69.6 | 4 | 1 | 85.7 |
| Risk of misinformation of patients | 4 | 1 | 87.0 | 4 | 1 | 85.7 |
| Ethical risks | 5 | 1 | 82.6 | 5 | 1 | 81.0 |
| Risk of biased decisions | 4 | 1 | 87.0 | 4 | 1 | 81.0 |
| Risk of inaccurate communication | 4 | 1.5 | 69.6 | 4 | 1 | 71.4 |
| Lack of explainability of systems decision-making processes | 4 | 1.5 | 73.9 | 4 | 2 | 71.4 |
| Risk of increasing health inequities | 4 | 1.5 | 69.6 | 4 | 3 | 66.7 |
| Limited interoperability of generated outputs | 4 | 1 | 65.2 | 4 | 1 | 61.9 |
| Risk of dehumanization of care | 4 | 2 | 56.5 | 4 | 2 | 61.9 |
| Risk of errors (HCPs) | 4 | 1 | 65.2 | 4 | 1 | 57.1 |
| Negative clinical outcomes | 4 | 1 | 65.2 | 4 | 2 | 57.1 |
| Risk of information overload of patients | 4 | 2 | 52.2 | 3 | 2 | 47.6 |
| Risk of information overload of HCPs | 3 | 2 | 39.1 | 3 | 2 | 42.9 |
| *Risks in medical profession* | | | | | | |
| Overconfidence in LLMs | 4 | 2 | 65.2 | 4 | 0 | 76.2 |
| Impact on jobs in healthcare sector | 4 | 2 | 69.6 | 4 | 2 | 71.4 |
| Misdiagnosis due to wrong generated results | 4 | 1 | 56.5 | 4 | 1 | 66.7 |
| Liability for error made by LLM-based systems | 4 | 1 | 60.9 | 4 | 1 | 61.9 |
| Lack of understanding of the underlying technology | 4 | 1 | 73.9 | 4 | 2 | 61.9 |
| Risk of losing knowledge and competencies | 3 | 1 | 43.5 | 3 | 2 | 42.9 |
| Risk of attempts to replace healthcare practitioners with some tools | 3 | 2 | 30.4 | 3 | 2 | 38.1 |
| Loss of communication skills | 2 | 1 | 17.4 | 2 | 2 | 28.6 |
| Loss of trust of patients in HCPs | 3 | 1.5 | 26.1 | 3 | 2 | 28.6 |
| Reduced need for medical professionals | 2 | 2 | 17.4 | 2 | 2 | 9.5 |
| *Risks for patients* | | | | | | |
| Risk of inaccurate communication | 4 | 1.5 | 69.6 | 4 | 1 | 71.4 |
| Lack of transparency of system use | 4 | 1 | 69.6 | 4 | 2 | 61.9 |
| Wrong personal health decisions due to the use of unverified information | 4 | 1 | 65.2 | 4 | 1 | 61.9 |
| Accessibility issues | 4 | 2 | 56.5 | 4 | 2 | 52.4 |
| Loss of patient-professional contact | 3 | 2 | 43.5 | 4 | 2 | 52.4 |
| Incorrect treatment plans | 4 | 1 | 56.5 | 4 | 2 | 52.4 |
| Incorrect diagnoses | 3 | 1 | 43.5 | 3 | 2 | 47.6 |
| Loss of trust in HCPs | 3 | 2 | 30.4 | 3 | 2 | 28.6 |
| *Risks related to data protection* | | | | | | |
| Use of unregulated cloud services may risk data security and privacy | 4 | 2 | 69.6 | 4 | 1 | 85.7 |
| Disclosure of sensitive patient data during training and inference | 4 | 0.5 | 78.3 | 4 | 0 | 85.7 |
| Breach of patient confidentiality | 4 | 2 | 65.2 | 4 | 0 | 81.0 |
| Fraudulent use of information | 4 | 2 | 65.2 | 4 | 1 | 76.2 |
| Vulnerabilities in data storage systems or communication channels | 4 | 2 | 69.6 | 4 | 0 | 76.2 |
| Risk of individual patient data may be accessed or used inappropriately | 4 | 1 | 60.9 | 4 | 0 | 76.2 |
| Breach of GDPR | 4 | 2 | 69.6 | 4 | 1 | 71.4 |
| Risk of patient reidentification | 4 | 2 | 65.2 | 4 | 1 | 71.4 |
| Uncontrolled access by third parties | 4 | 2 | 60.9 | 4 | 2 | 66.7 |
| *Risks for the health IT field* | | | | | | |
| Unresolved responsibilities for system error or wrong outputs hamper adoption of LLM-based systems | 4 | 1 | 78.3 | 4 | 1 | 85.7 |
| Developing and delivering solutions compliant with regulations is complex for health IT companies | 4 | 1.5 | 69.6 | 4 | 0 | 81.0 |
| Competitive pressure leads to market release of LLM-based systems of low quality | 4 | 1.5 | 73.9 | 4 | 0 | 81.0 |
| Lack of understanding of clinical risks leads to systems that can harm patients | 4 | 1.5 | 65.2 | 4 | 0 | 76.2 |
| Financial constraints at healthcare institutions for maintenance of LLM-based systems will hamper adoption of high-quality systems | 4 | 2 | 52.2 | 4 | 1 | 71.4 |
| Lack of skilled workers for developing LLM-based systems will hamper development of high-quality systems | 4 | 1 | 69.6 | 4 | 1 | 71.4 |
| A missing standard quality assessment framework for LLM-based systems will lead to low quality systems released to market | 3 | 2.5 | 34.8 | 3 | 3 | 47.6 |
| LLM-based systems will lack integration into clinical systems | 3 | 2 | 39.1 | 4 | 1 | 66.7 |
| Missing reimbursement models for LLM-based systems hampers the adoption of technology | 3 | 2.5 | 34.8 | 3 | 3 | 47.6 |
| Companies lack of competence to ensure development of systems compliant with regulations | 3 | 1 | 43.5 | 3 | 1 | 47.6 |

## IV.4 Needs for future adoption and implementation of high-quality LLM-based systems

| **Item** | **Round 2** | | | **Round 3** | | |
| --- | --- | --- | --- | --- | --- | --- |
|  | **Median** | **IQR** | **% 4 or 5 scores** | **Median** | **IQR** | **% 4 or 5 scores** |
| Successful adoption in practice requires training of HCPs | 5 | 1 | 100 | 5 | 1 | 100 |
| Successful adoption in practice requires quality assessment standards | 5 | 1 | 100 | 5 | 1 | 100 |
| Successful adoption in practice requires regulations on data privacy for such systems | 5 | 1 | 100 | 5 | 1 | 95.2 |
| Successful adoption in practice requires proper standards for data security and data privacy | 5 | 1 | 100 | 5 | 1 | 95.2 |
| Successful adoption in practice requires algorithmovigilance | 5 | 1 | 91.3 | 5 | 1 | 95.2 |
| Successful adoption in practice requires training of health IT personnel | 5 | 1 | 100 | 5 | 1 | 95.2 |
| Successful adoption of LLM-based solutions supporting the decision making process in practice requires co-design of new workflows with healthcare professionals | na | na | na | 5 | 1 | 81.0 |
| Successful adoption in practice requires regulations on data ownership | 5 | 1 | 100 | 5 | 1 | 87.5 |
| Successful adoption in practice requires guidelines for interpretation of results of LLM-based systems and their use in clinical practice | 5 | 1 | 95.7 | 5 | 1 | 85.7 |
| Successful adoption in practice requires a cultural change in healthcare | 4 | 1 | 78.3 | 5 | 1 | 81.0 |
| Successful adoption in practice requires integration with existing EHRs if the LLM-based solution supports the decision-making process | na | na | na | 5 | 1 | 81.0 |
| Successful adoption in practice requires adaptation of jobs in the healthcare domain | 4 | 0.5 | 73.9 | 4 | 1 | 81.0 |
| Successful adoption in practice requires reimbursement models for LLM-based systems and their use in healthcare | 4 | 2 | 69.6 | 4 | 0 | 76.2 |

## IV.5 Reliability of systems based on LLMs

| **Item** | **Round 2** | | | **Round 3** | | |
| --- | --- | --- | --- | --- | --- | --- |
|  | **Median** | **IQR** | **% 4 or 5 scores** | **Median** | **IQR** | **% 4 or 5 scores** |
| The system is tested in real settings | 5 | 0.5 | 95.7 | 5 | 0 | 100 |
| The system outputs are reproducible | 5 | 1 | 95.7 | 5 | 1 | 100 |
| The system outputs are reliable | 5 | 1 | 100 | 5 | 1 | 100 |
| The system is robust against a wide range of inputs | 5 | 1 | 95.7 | 5 | 1 | 100 |
| Quality of the data underlying the system is ensured | 5 | 1 | 100 | 5 | 1 | 100 |
| The system is tested in simulated settings with real users | 4 | 1 | 95.7 | 5 | 1 | 100 |
| The system is validated for accuracy | 4 | 1 | 95.7 | 4 | 1 | 100 |
| The system meets federal regulations | 5 | 1 | 95.7 | 5 | 1 | 95.2 |
| The system is interoperable with existing healthcare systems | 5 | 1 | 95.7 | 5 | 1 | 95.2 |
| Control mechanisms or human in the loop are established to ensure reliability of LLM-based systems | 5 | 1 | 91.3 | 5 | 1 | 90.5 |
| A standardized quality assessment is available for the system | 4 | 1 | 87.0 | 5 | 1 | 90.5 |
| The system has been proven to be non-inferior in a variety of clinical settings | 4 | 1 | 87.0 | 4 | 1 | 90.5 |
| Explanations on the reasoning behind model predictions and recommendations are available | 4 | 1 | 87.0 | 4 | 1 | 90.5 |
| The system can solve easy routine tasks with nearly 100% accuracy | 4 | 1 | 82.6 | 4 | 1 | 81.0 |

## IV.6 Future of LLMs

| **Item** | **Round 1** | | | **Round 2** | | | **Round 3** | | |
| --- | --- | --- | --- | --- | --- | --- | --- | --- | --- |
|  | **Median** | **IQR** | **% 4 or 5 scores** | **Median** | **IQR** | **% 4 or 5 scores** | **Median** | **IQR** | **% 4 or 5 scores** |
| LLMs will be combined with other technologies in future health applications | 5 | 1 | 96.4 | 4 | 1 | 95.7 | 4 | 1 | 100 |
| Applications based on LLMs will be used by healthcare professionals | 4 | 0.25 | 89.3 | 4 | 1 | 100 | 4 | 1 | 100 |
| LLMs will have an impact on future technologies in healthcare | 4 | 1 | 85.7 | 4 | 1 | 95.7 | 4 | 0 | 95.2 |
| Applications based on LLMs will be used by patients | 4 | 1 | 92.9 | 4 | 0.5 | 91.3 | 4 | 1 | 85.7 |
| LLMs will replace other technologies | 4 | 1.25 | 64.3 | 4 | 1 | 65.2 | 4 | 0 | 85.7 |
| The medical device regulation hampers the introduction of solutions based on LLMs | 4 | 2 | 53.6 | 4 | 1 | 52.2 | 4 | 2 | 66.7 |
| Solutions based on LLMs will help addressing the shortage of skilled health professionals | 4 | 1 | 71.0 | 4 | 1 | 56.5 | 4 | 1 | 66.7 |
| To what extent will future healthcare rely upon LLM-based solutions? | 4 | 0 | 82.1 | 4 | 0 | 82.6 | 4 | 1 | 57.1 |
| LLM-based digital health solutions will contribute to discrimination in healthcare because they rely upon biased data | 4 | 1 | 57.1 | 3 | 2 | 39.1 | 4 | 1 | 52.4 |
| I consider LLMs, specifically their resource consumption, ecologically sustainable | 3 | 1 | 35.7 | 3 | 2 | 30.4 | 3 | 2 | 33.3 |
| Students of medicine will lose competencies through the increased use of LLMs | 3 | 2.25 | 39.3 | 2 | 2 | 30.4 | 2 | 2 | 33.3 |
| The introduction of LLMs in digital health solutions will result in cost savings in the health sector | 3 | 1 | 39.3 | 3 | 1 | 43.5 | 3 | 1 | 28.6 |
| LLMs will be replaced by other technologies in the coming 5 years | 3 | 0.25 | 25.0 | 3 | 1 | 47.8 | 3 | 1 | 28.6 |
| Healthcare professionals (physicians, nurses) will lose competencies through the increased use of LLMs | 3 | 2 | 35.7 | 3 | 2 | 34.8 | 2 | 1 | 28.6 |
| Solutions based on LLMs will offend the sensibilities of healthcare professionals | 2.5 | 1 | 17.9 | 3 | 1 | 21.7 | 3 | 1 | 23.8 |
| Patients will lose competencies through the increased use of LLMs | 2 | 1 | 10.7 | 3 | 2 | 34.8 | 2 | 1 | 19.0 |
| Solutions based on LLMs will offend the sensibilities of patients | 2 | 1 | 10.7 | 2 | 1 | 17.4 | 3 | 1 | 9.5 |
| Solutions based on LLMs will offend the sensibilities of other people involved in the care process | 2 | 1 | 7.1 | 3 | 1 | 21.7 | 3 | 1 | 9.5 |
