# Supplementary material for: Potential of Large Language Models in Health Care: Delphi Study
Source: J Med Internet Res. 2024 May 13;26:e52399. doi: 10.2196/52399 (PMC11130776; doi:10.2196/52399)
Supplement: Multimedia Appendix 5 [file jmir_v26i1e52399_app5.docx]

# **Multimedia Appendix 5: Percentage of participants assigning each score in round 3**

## V.1 Perceived likelihood of the use LLM-based systems will support healthcare tasks

### V.1.1 Supporting in clinical trials


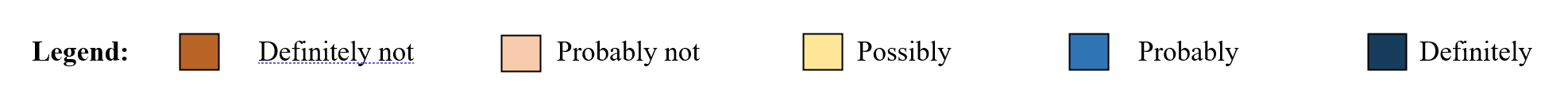


| 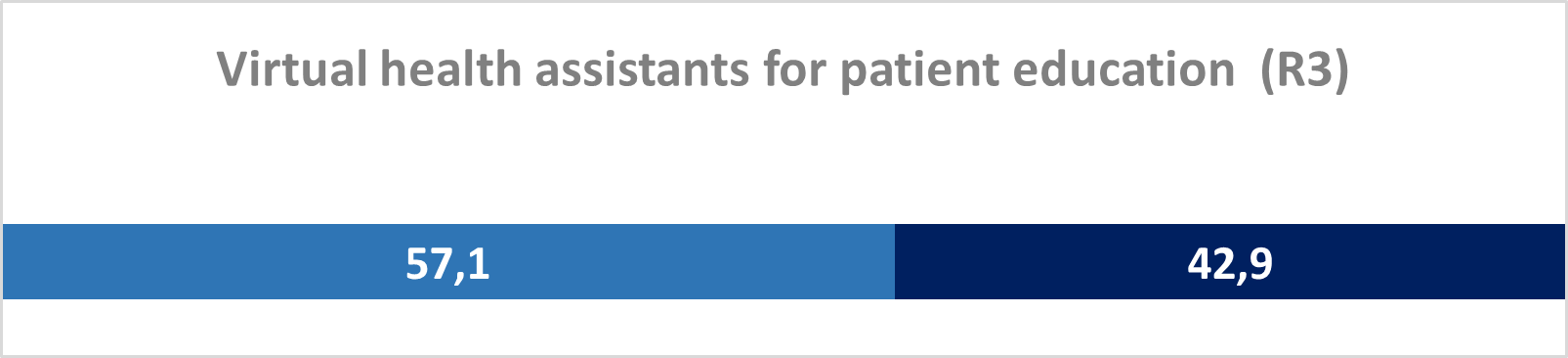 | 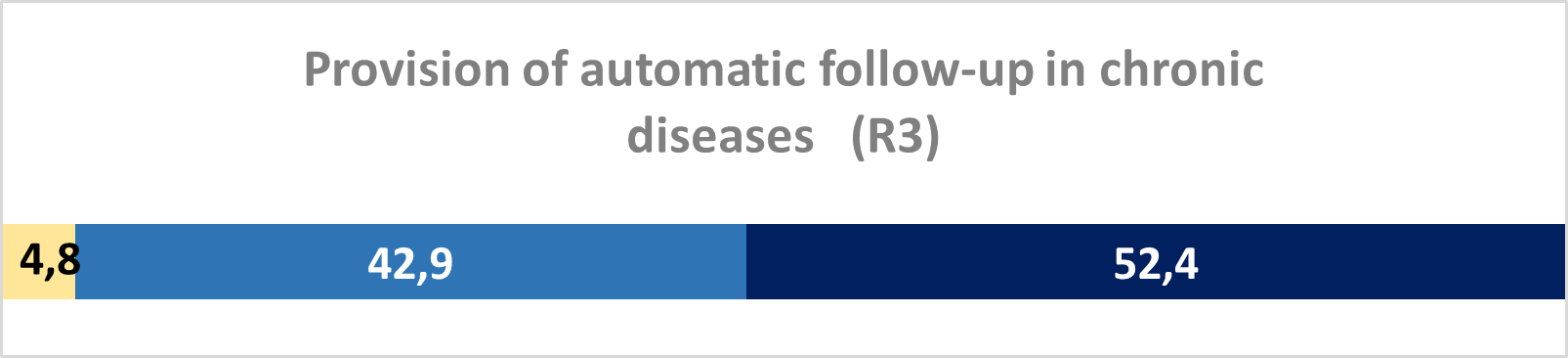 |
| --- | --- |
| 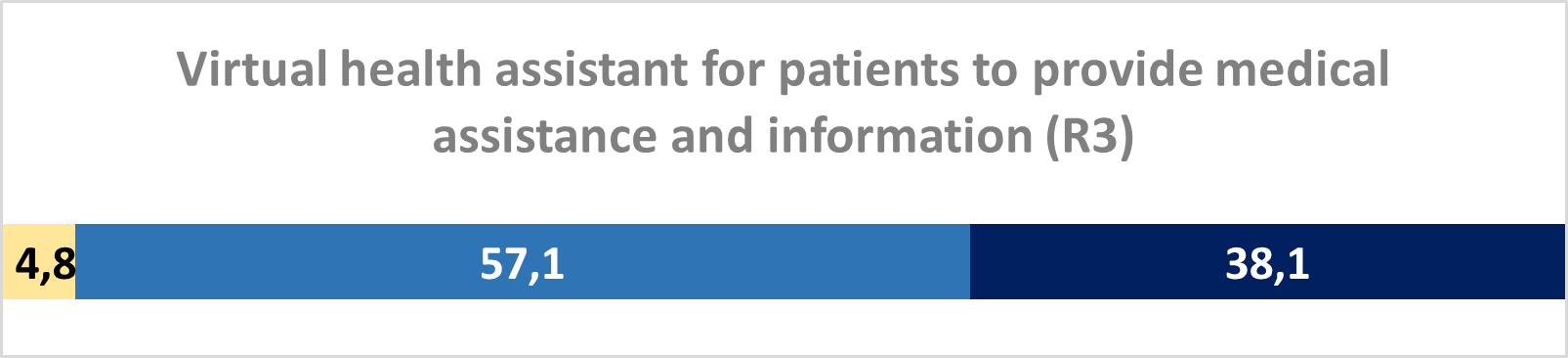 | 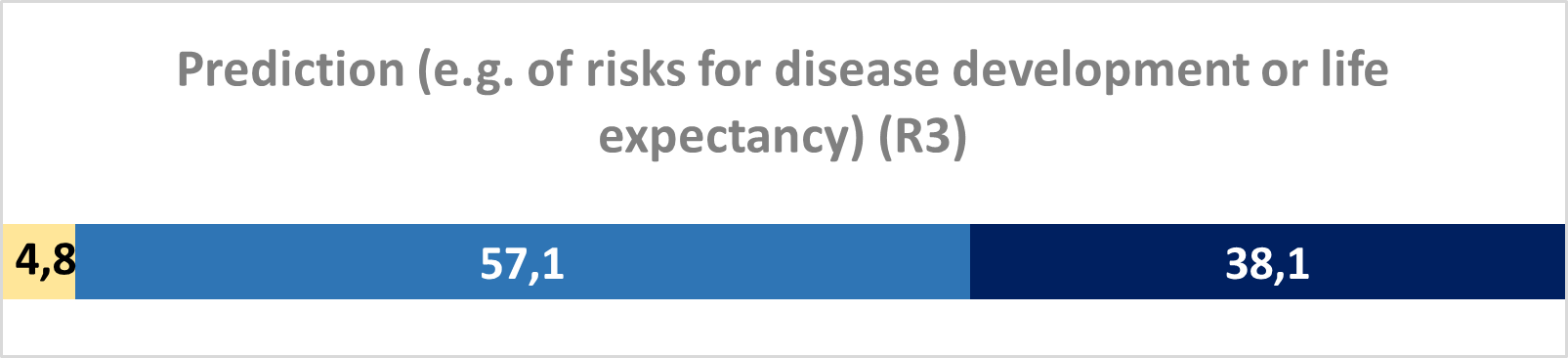 |
| 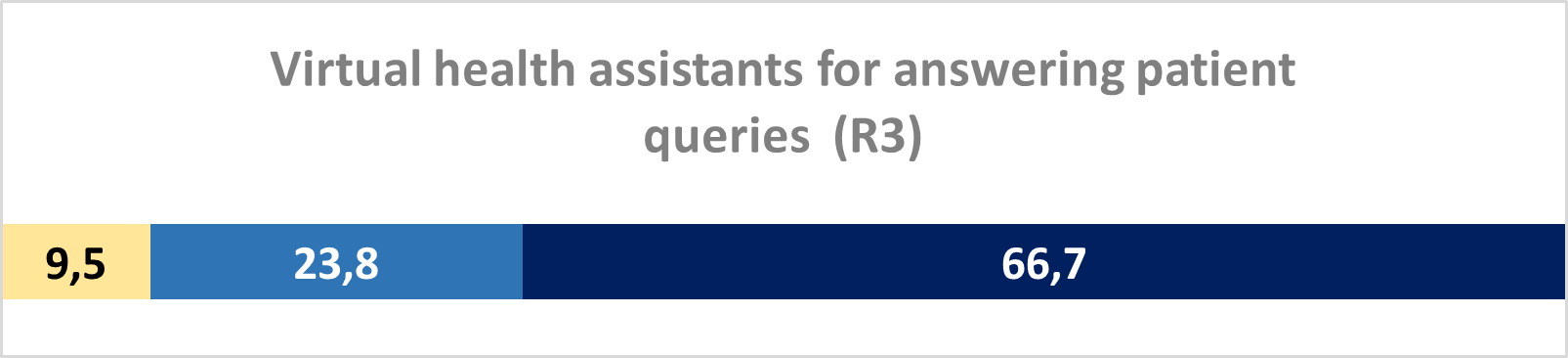 | 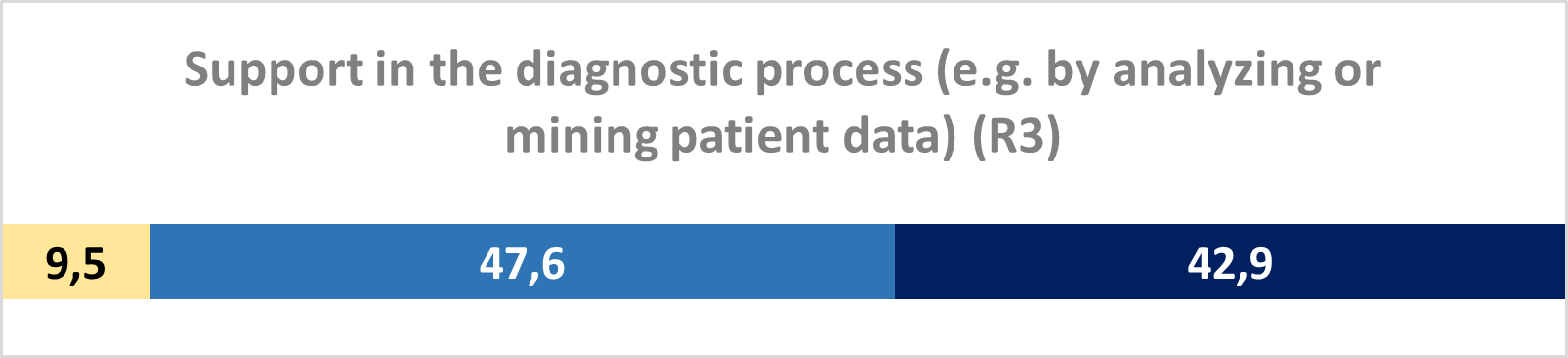 |
| 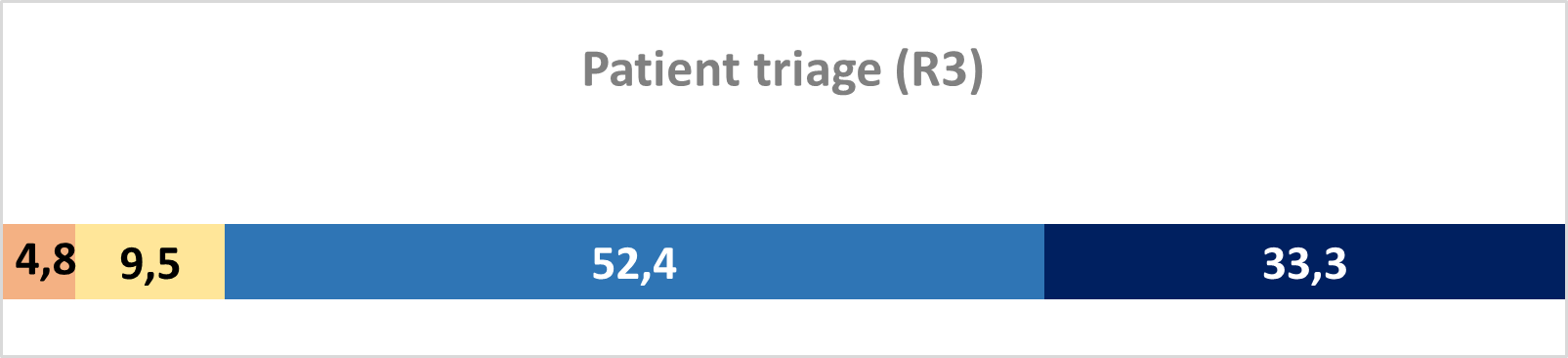 | 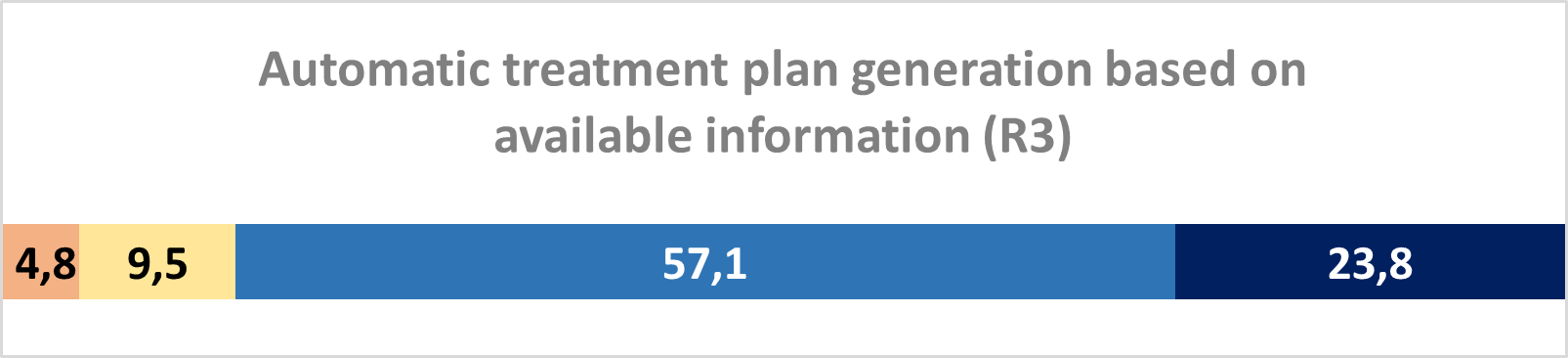 |
| 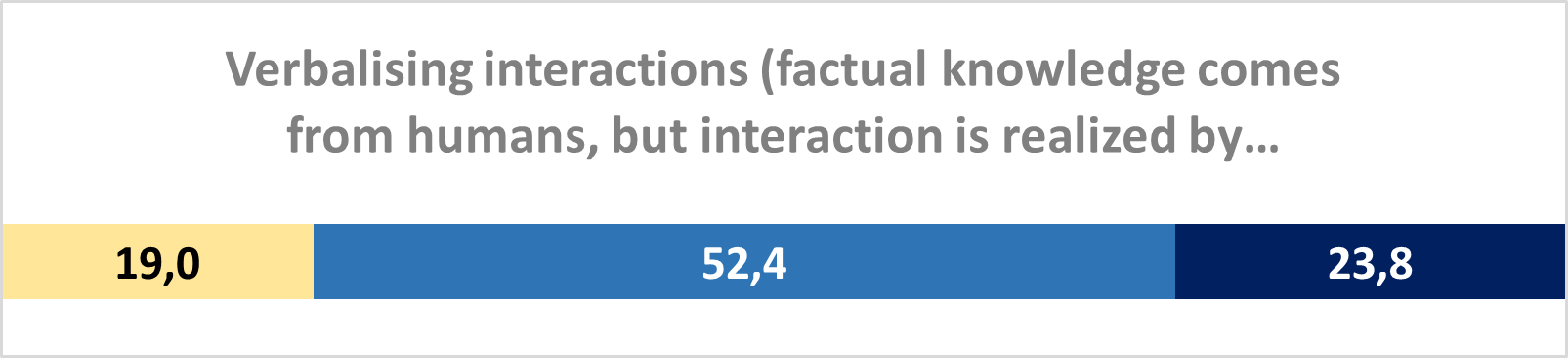 |  |

##

### V.1.2 Documentation tasks


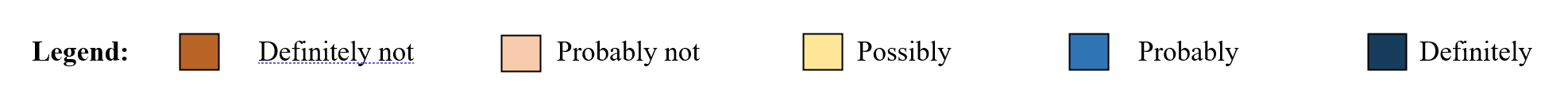


| 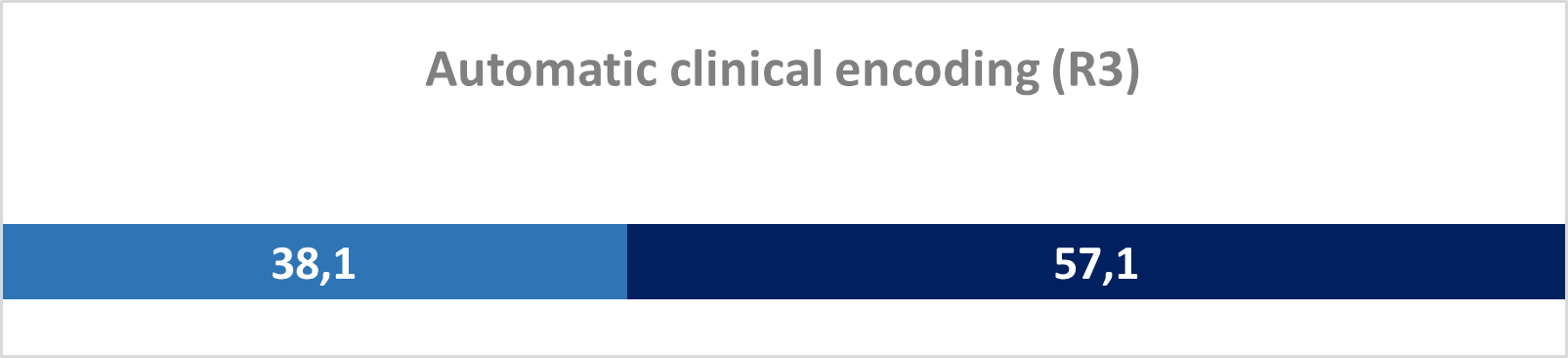 | 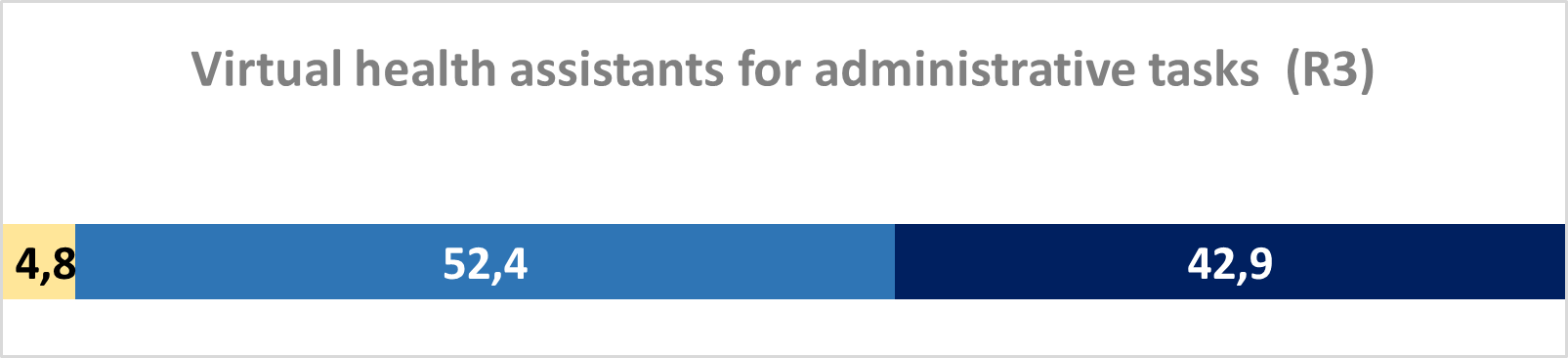 |
| --- | --- |
| 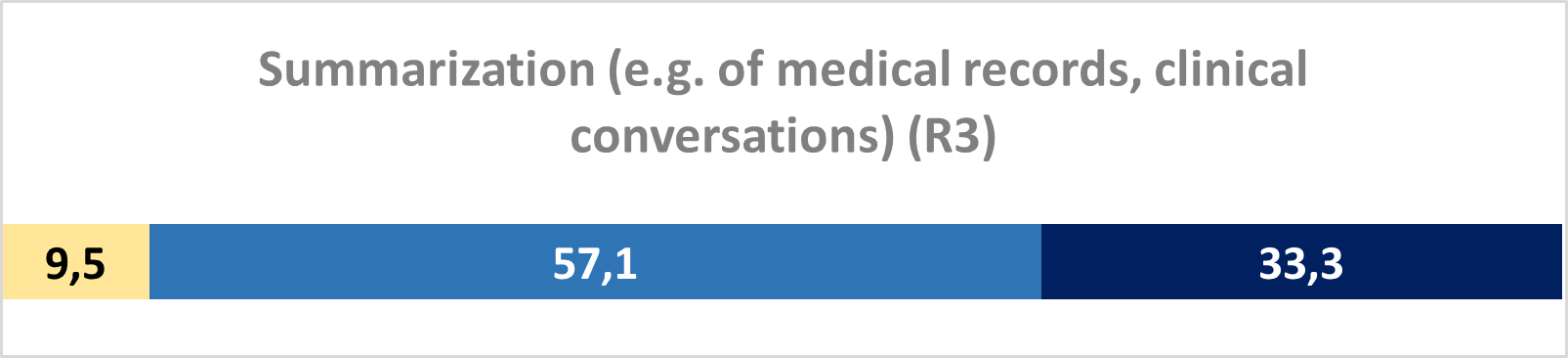 | 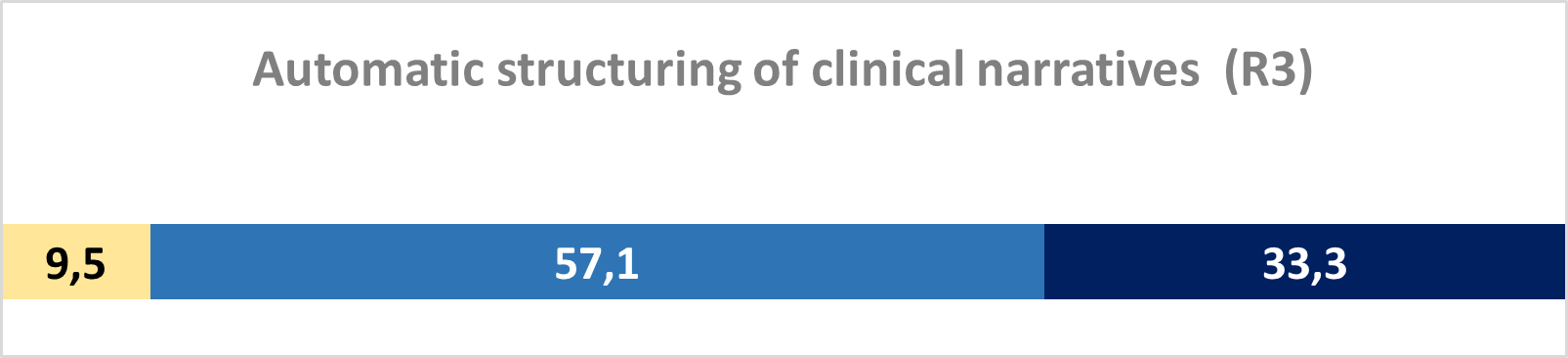 |
| 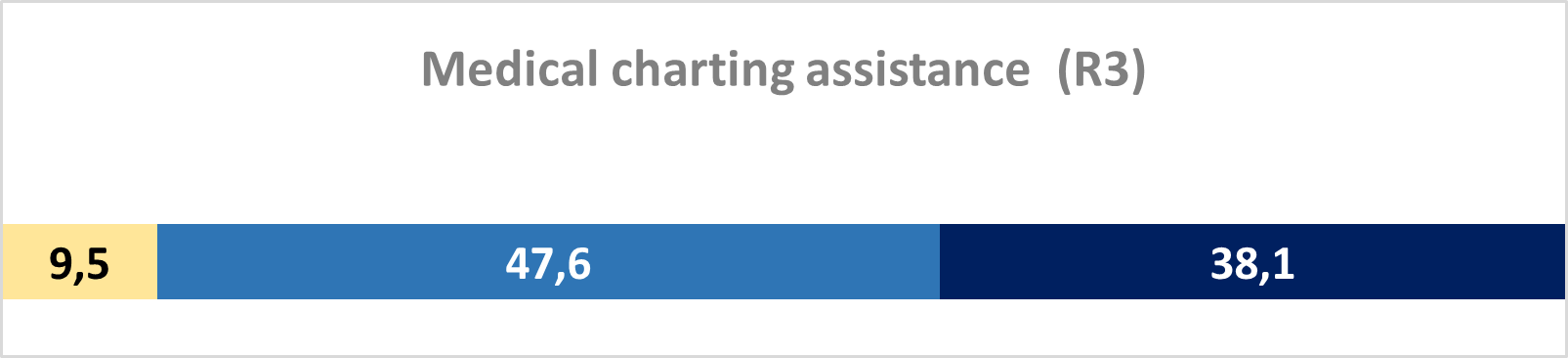 | 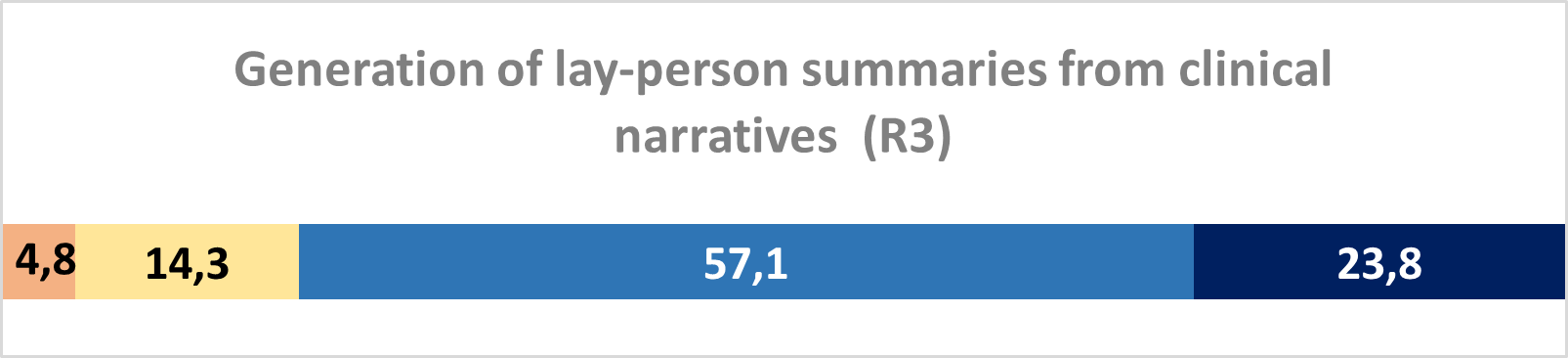 |

###

### V.1.3 Medical research and education


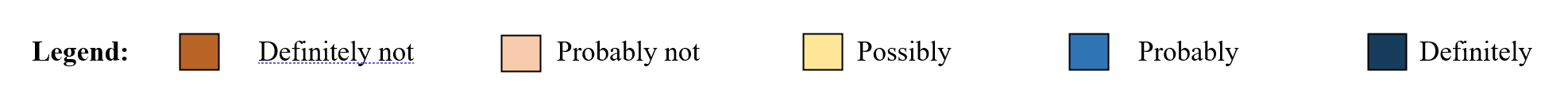


| 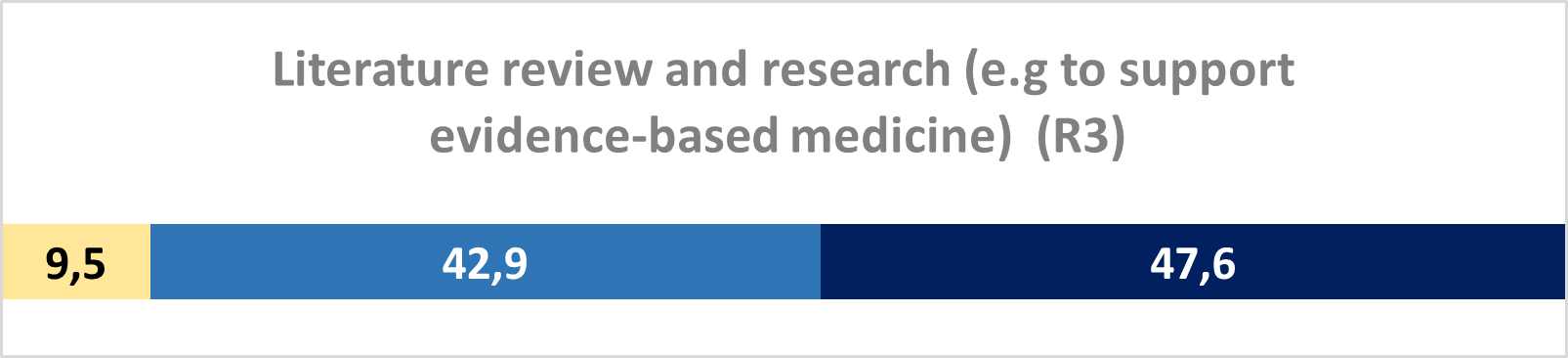 | 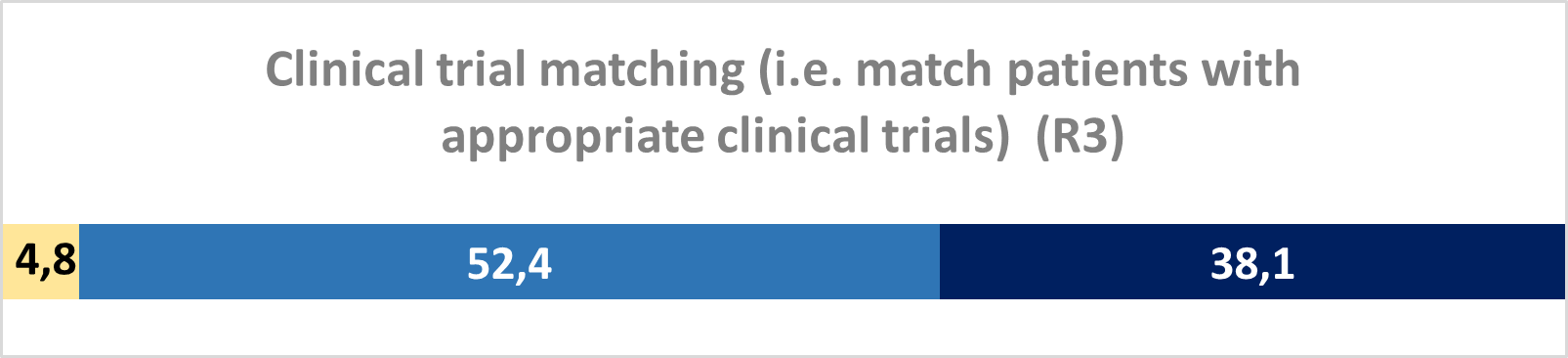 |
| --- | --- |
| 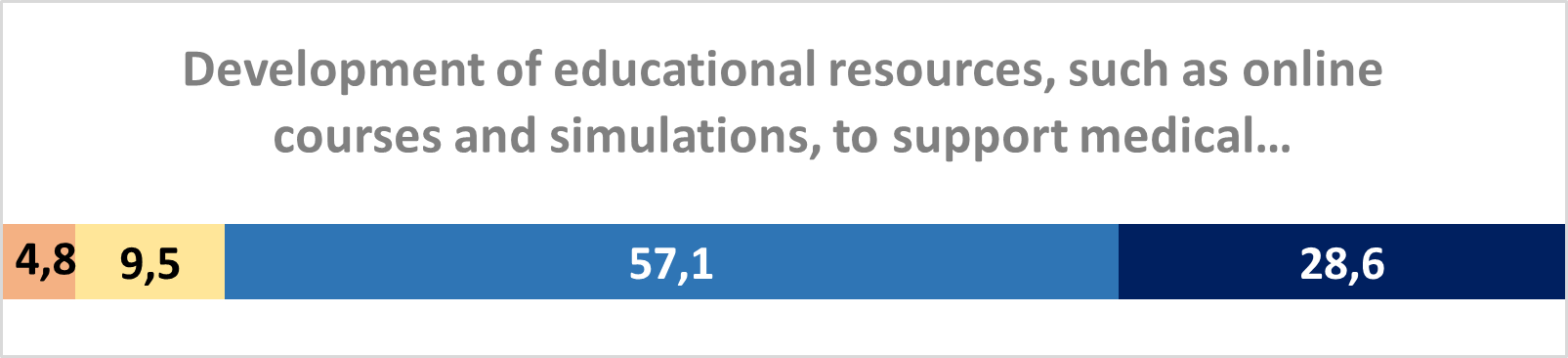 | 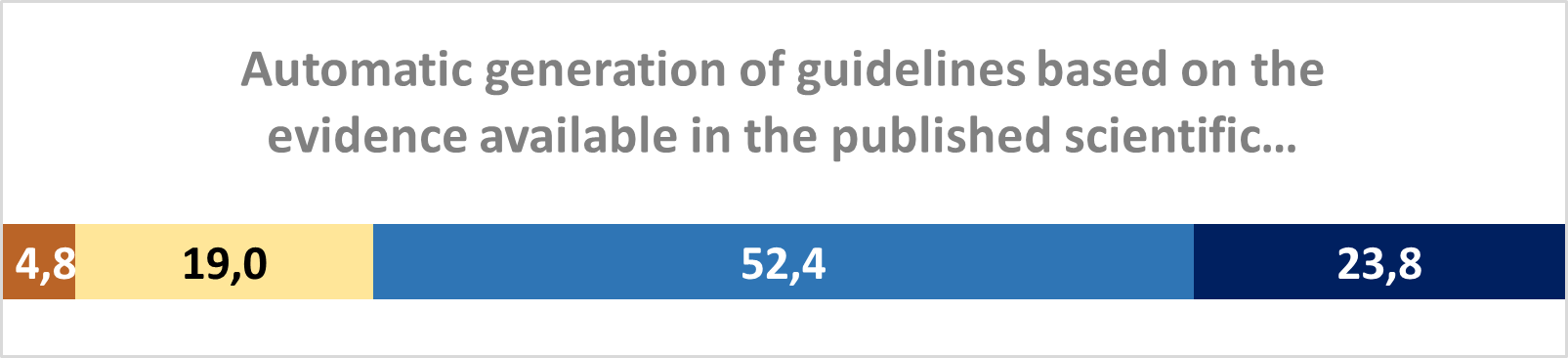 |
| 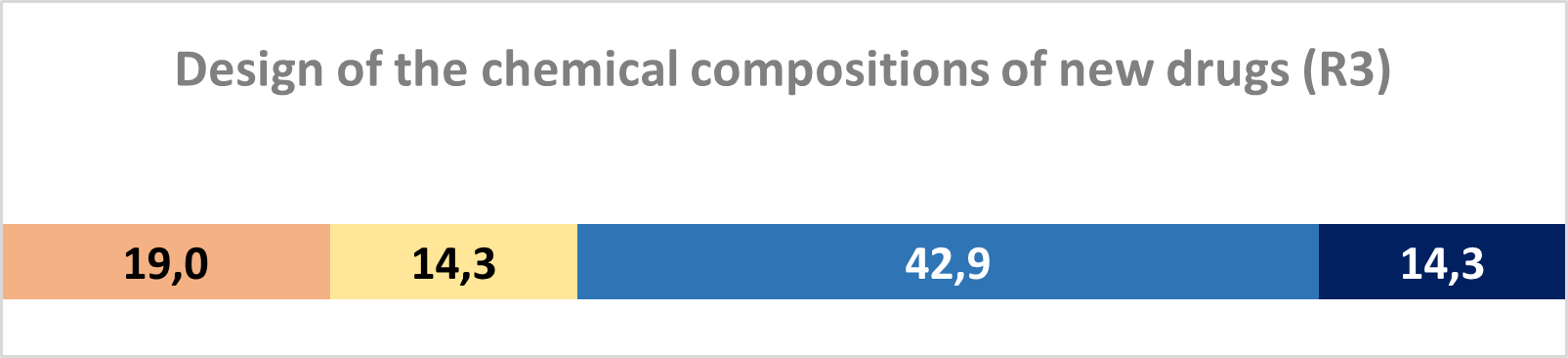 |  |

## V.2 Benefits of using LLM-based systems in healthcare


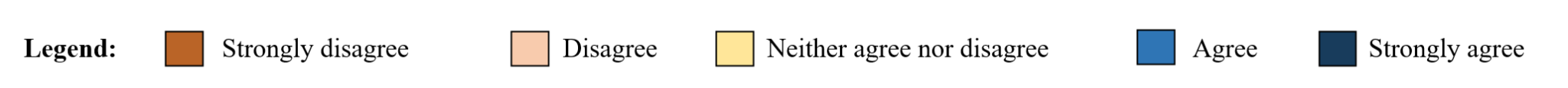


| 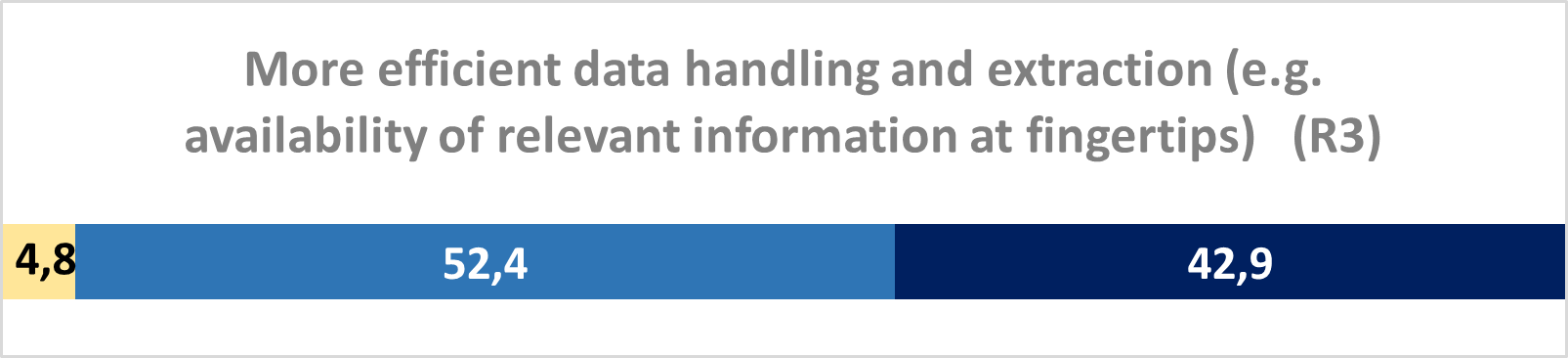 | 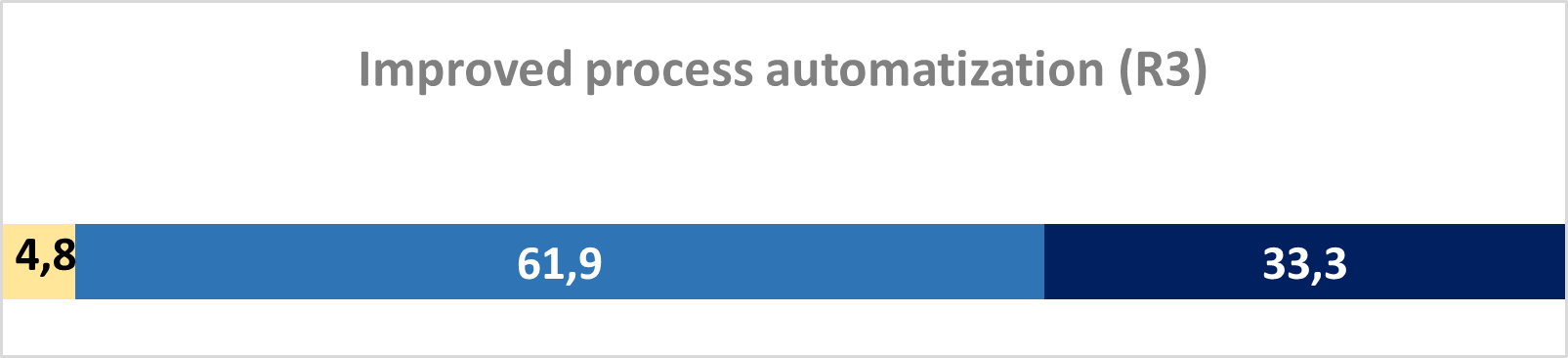 |
| --- | --- |
| 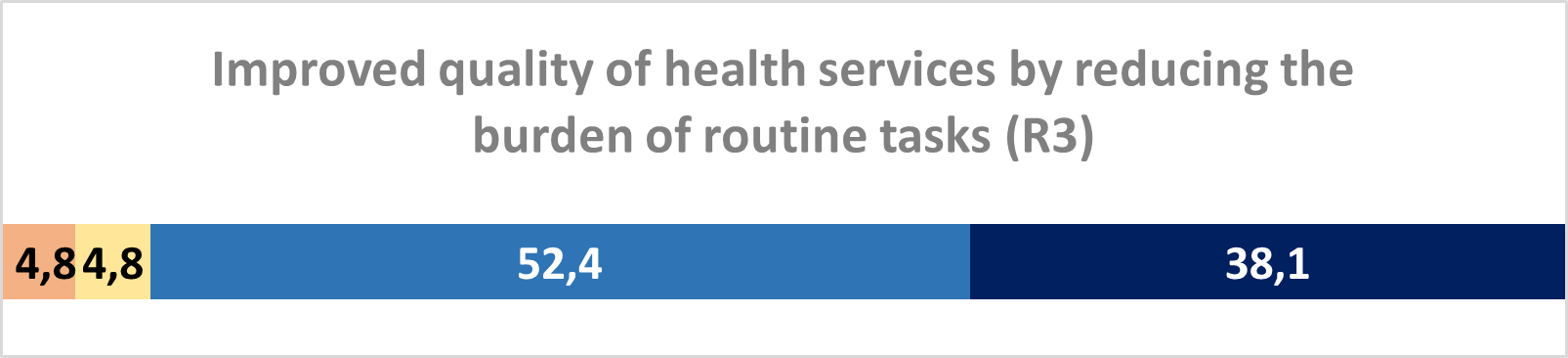 | 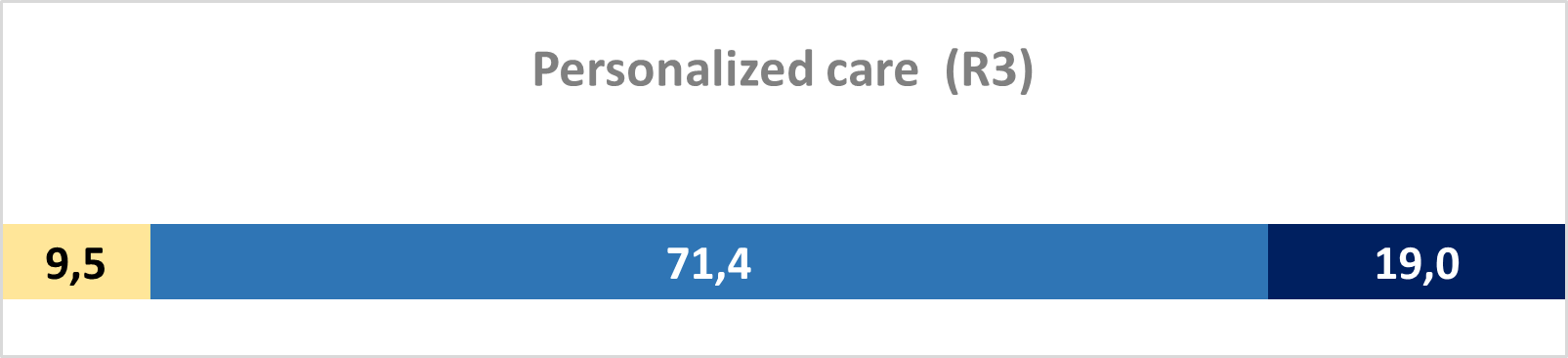 |
| 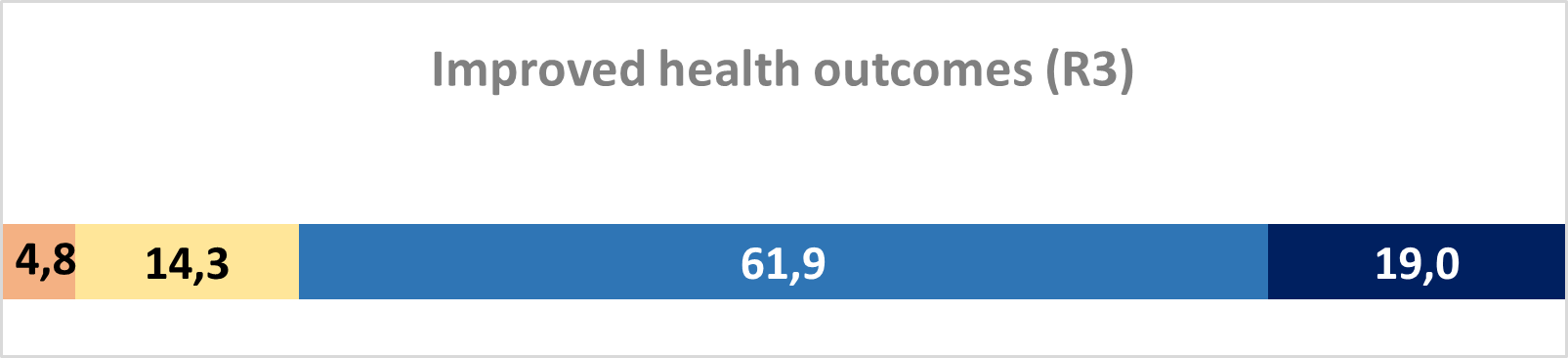 | 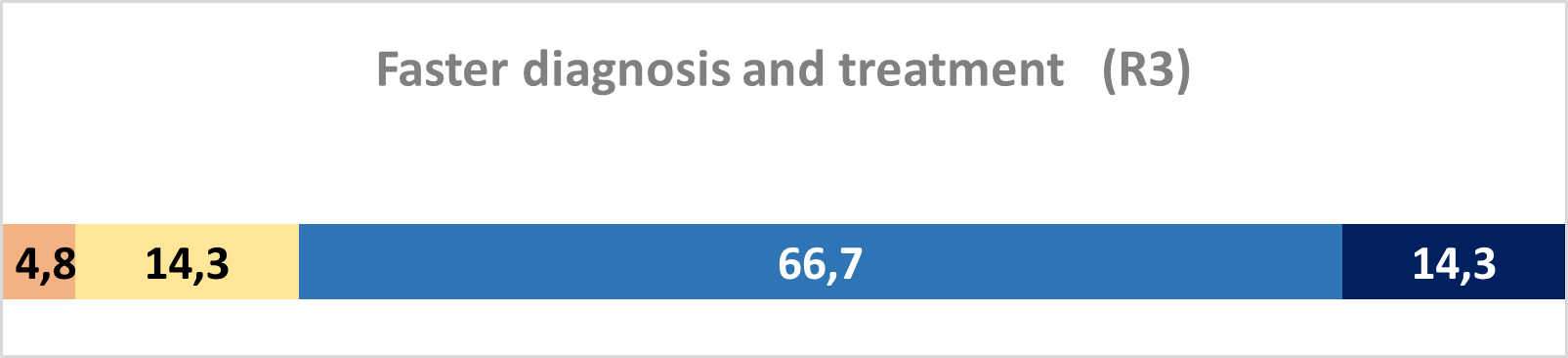 |
| 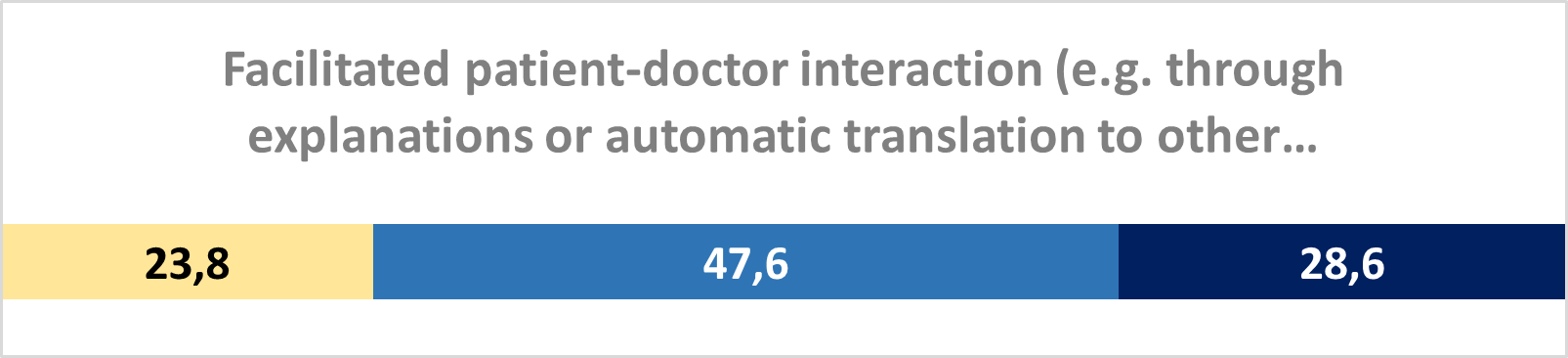 | 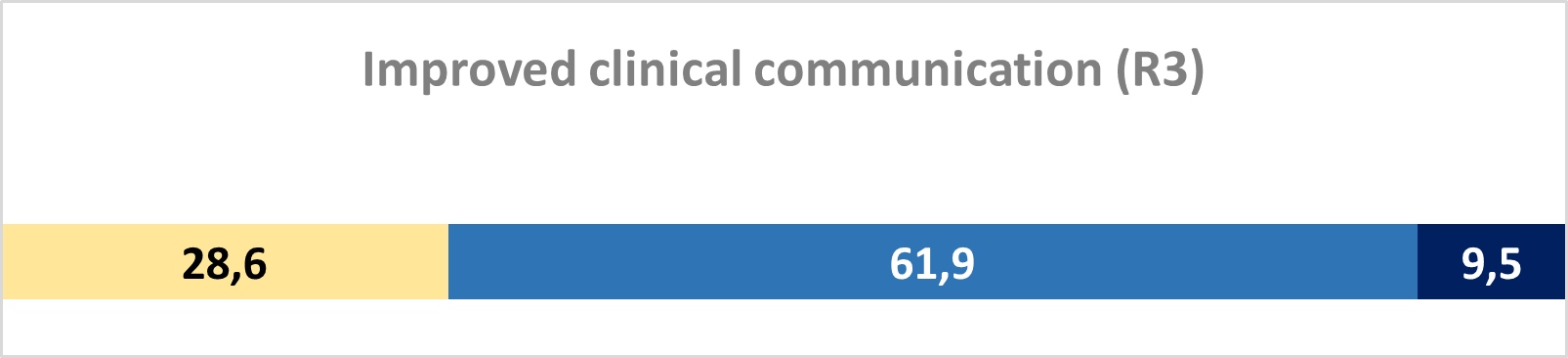 |
| 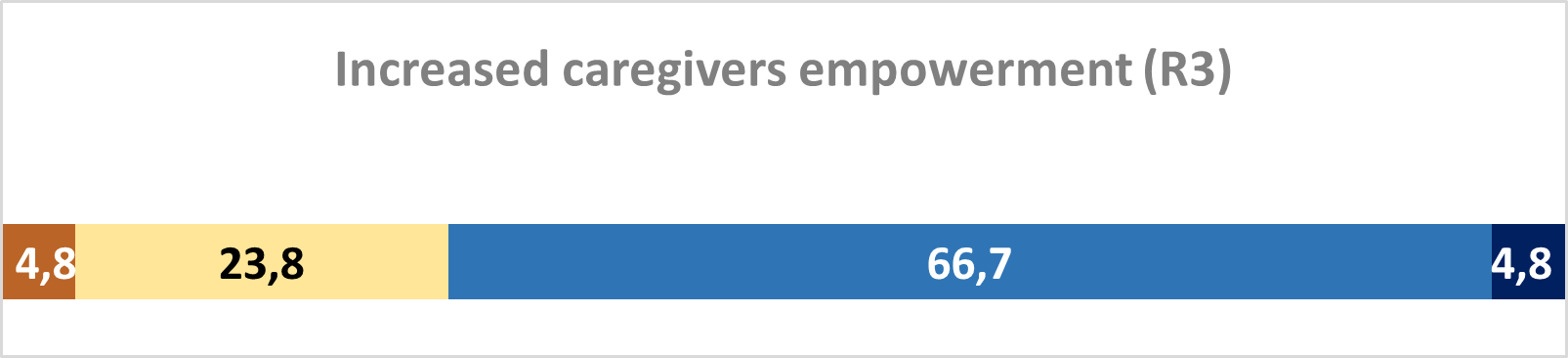 | 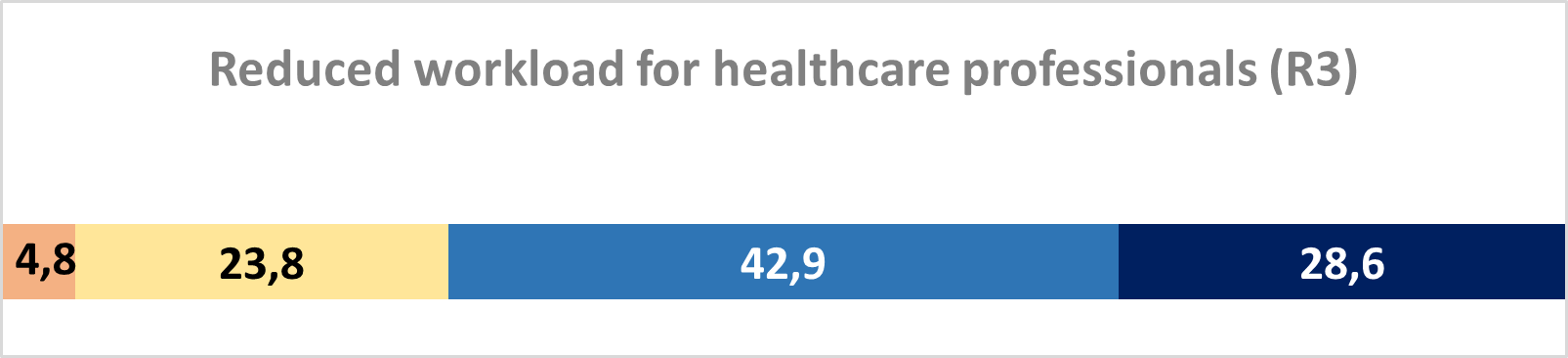 |
| 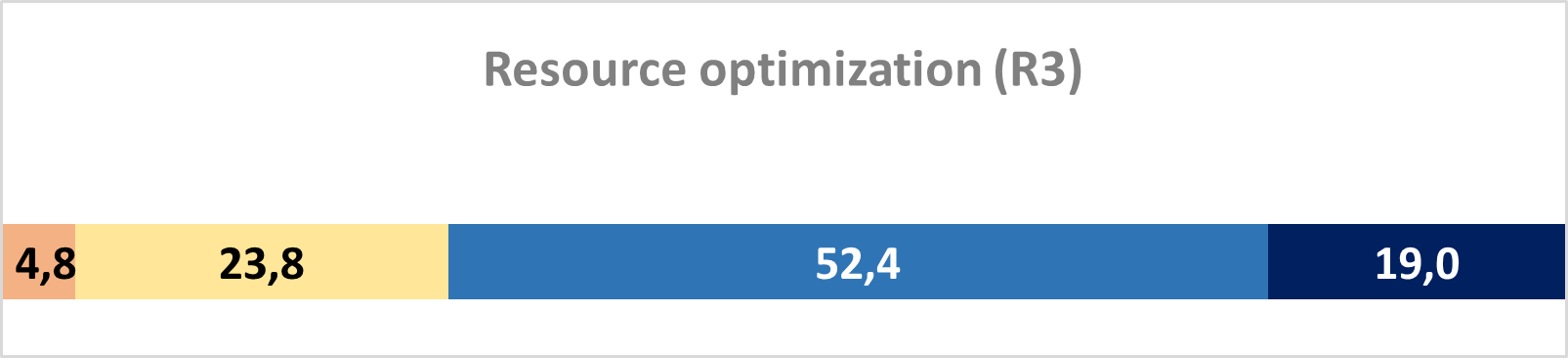 | 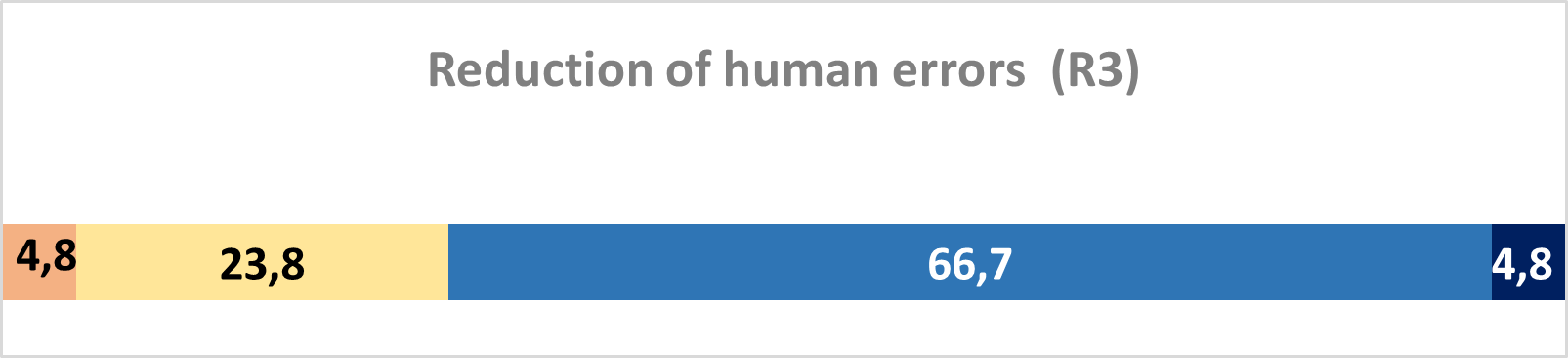 |
| 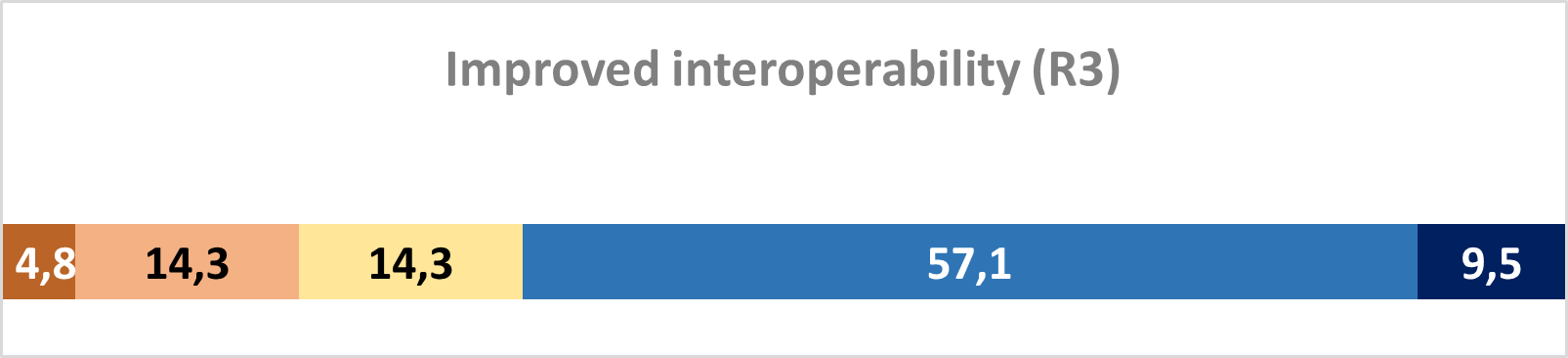 | 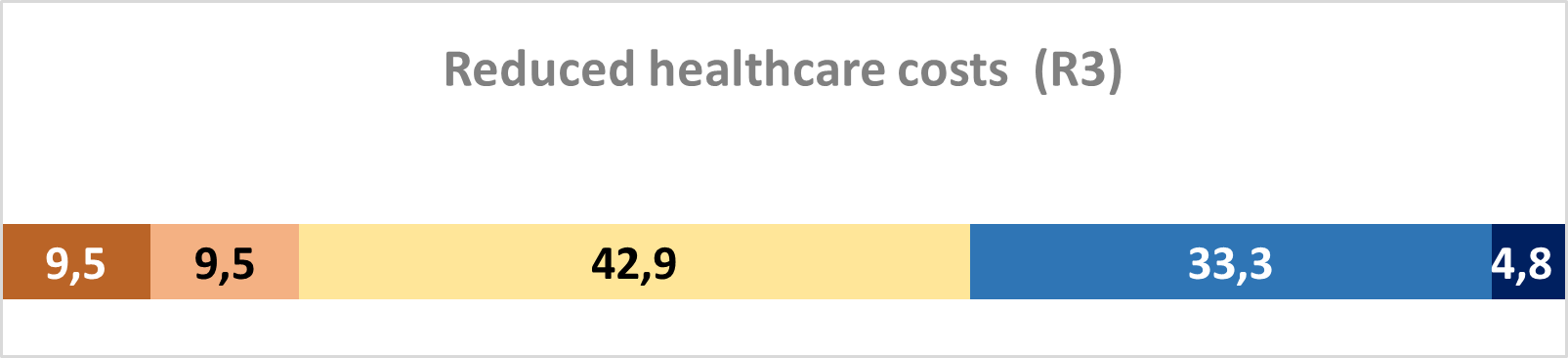 |

## V.3 Shortcomings and risks of LLM-based systems in healthcare

### V.3.1 Risks in healthcare


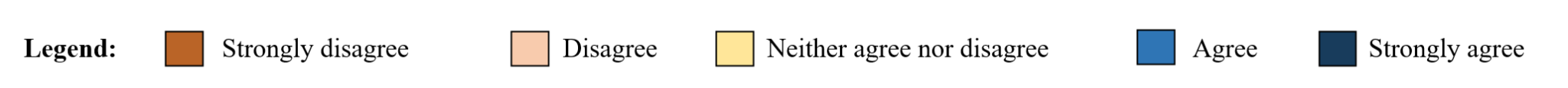


| 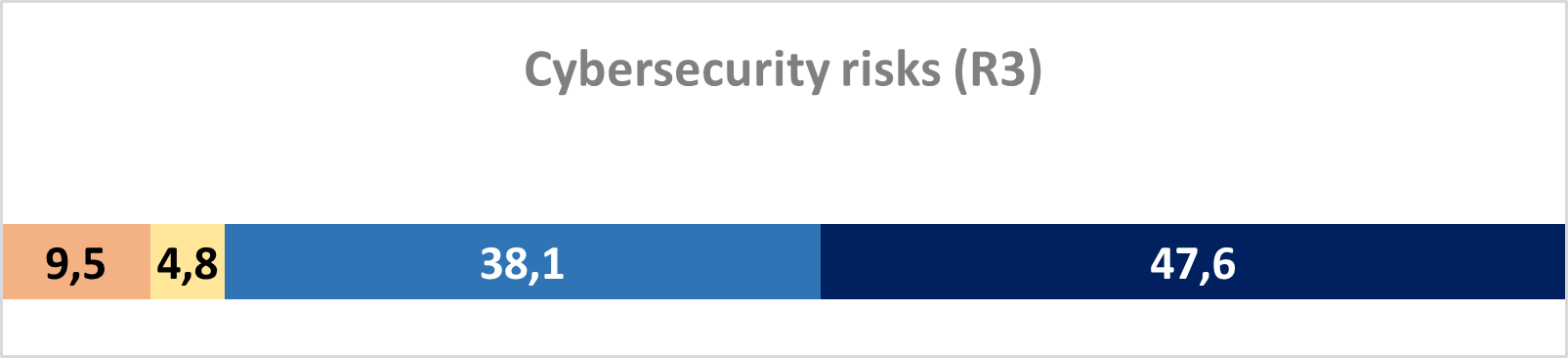 | 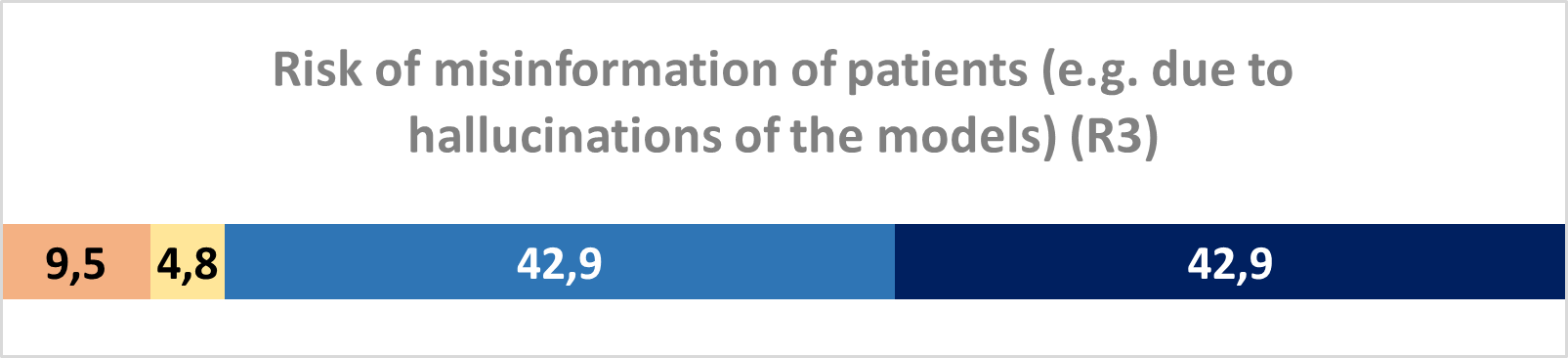 |
| --- | --- |
| 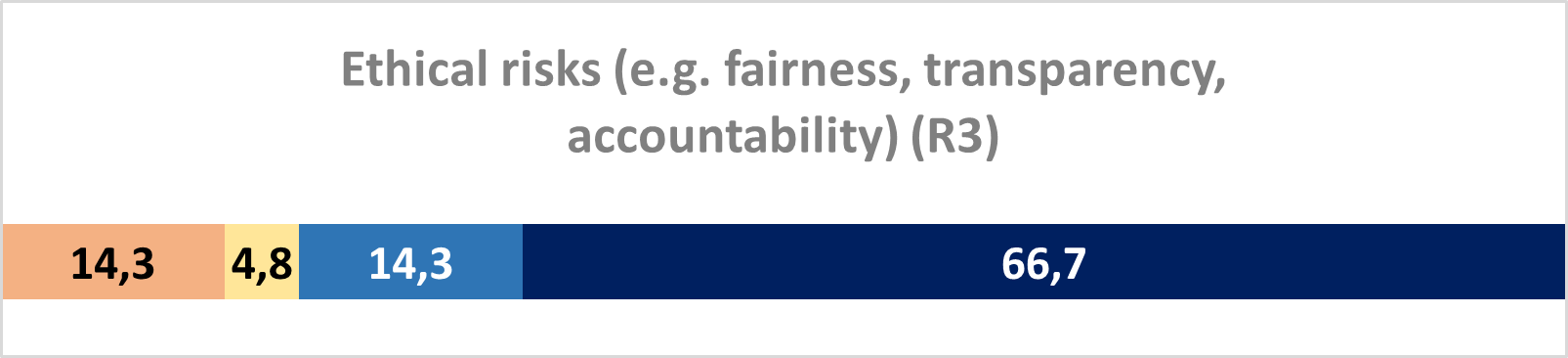 | 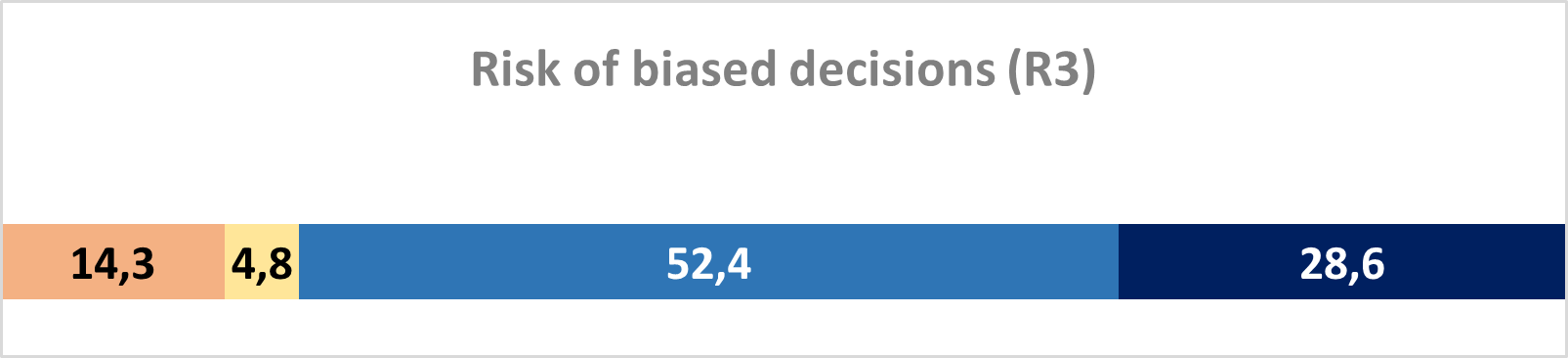 |
| 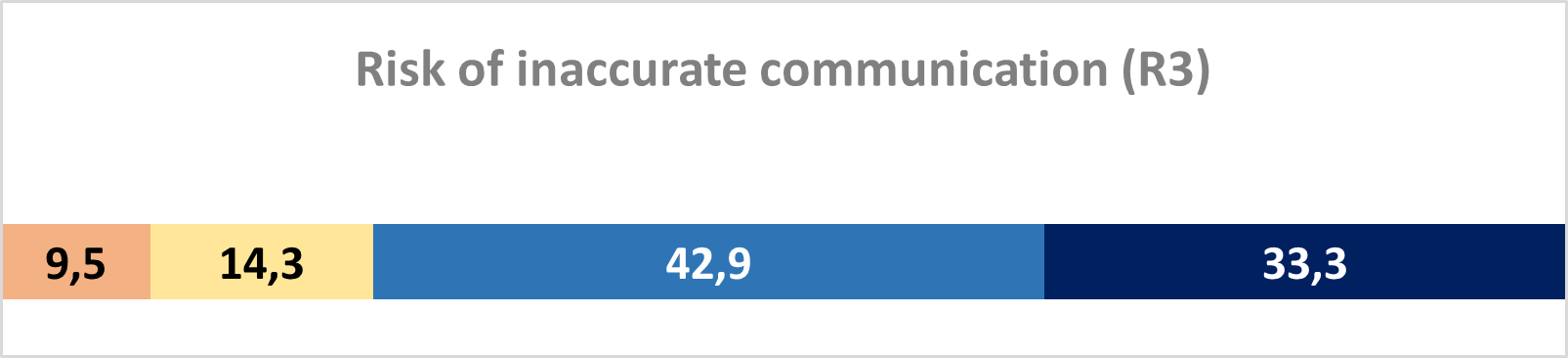 | 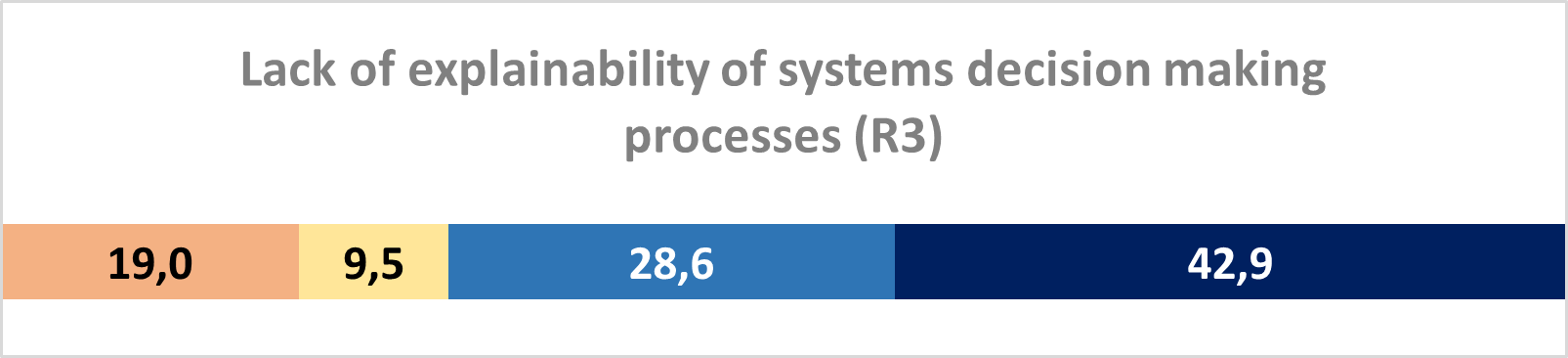 |
| 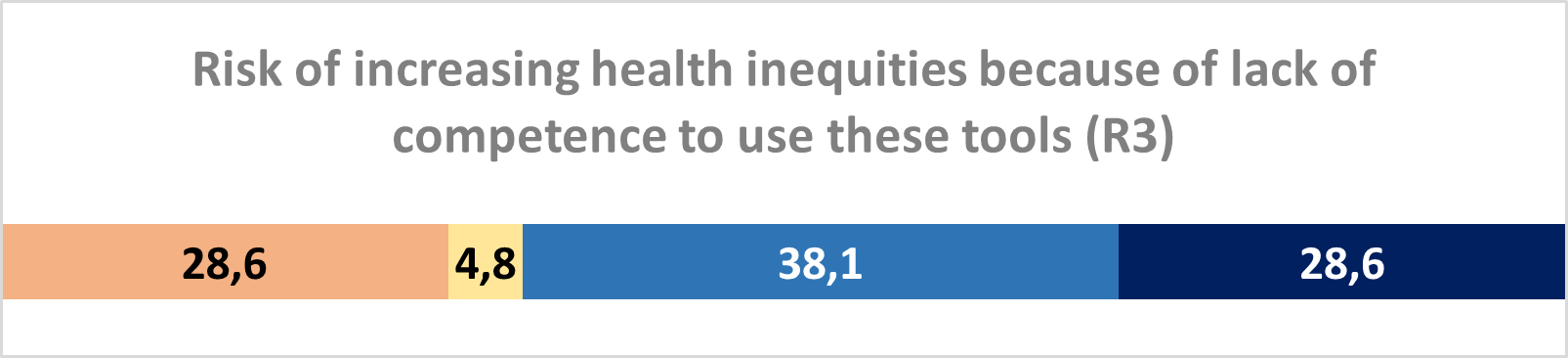 | 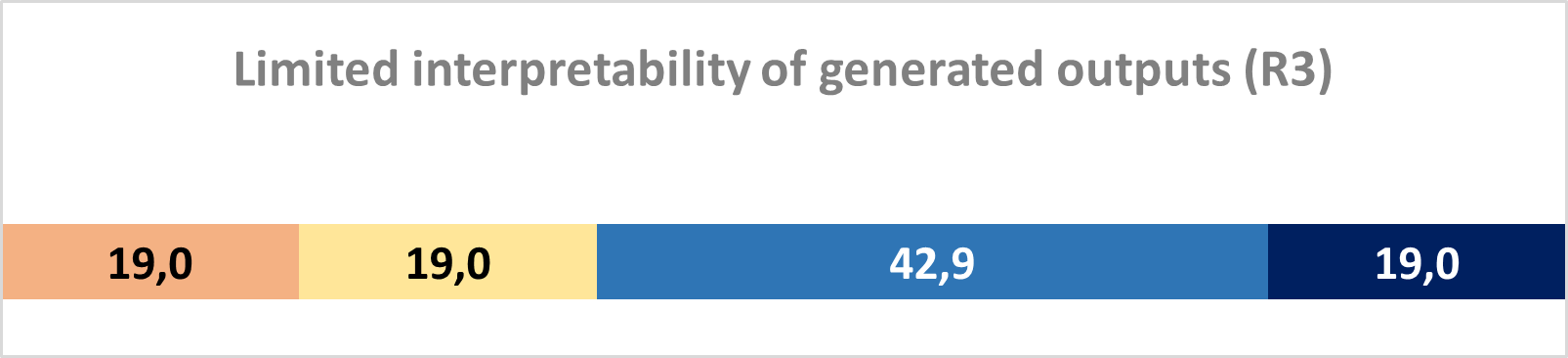 |
| 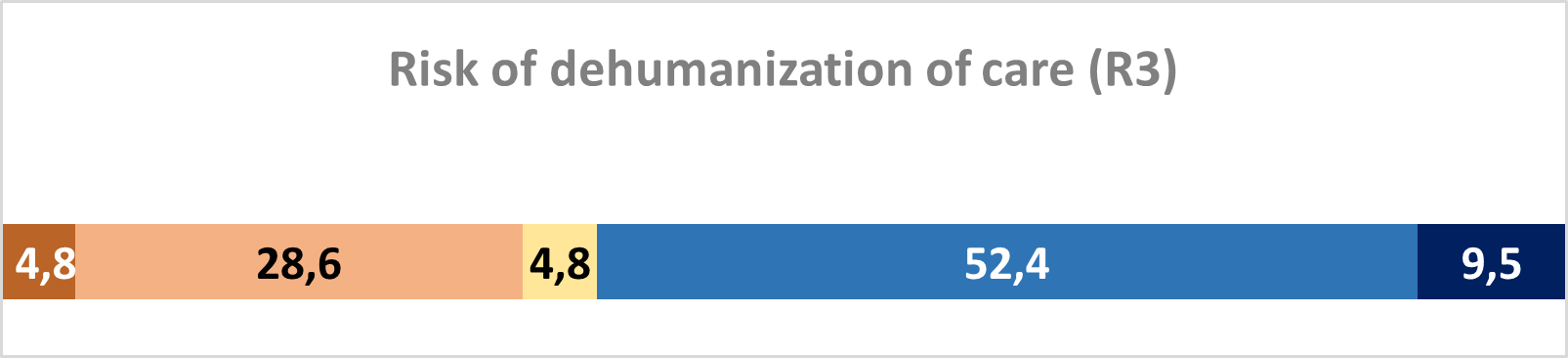 | 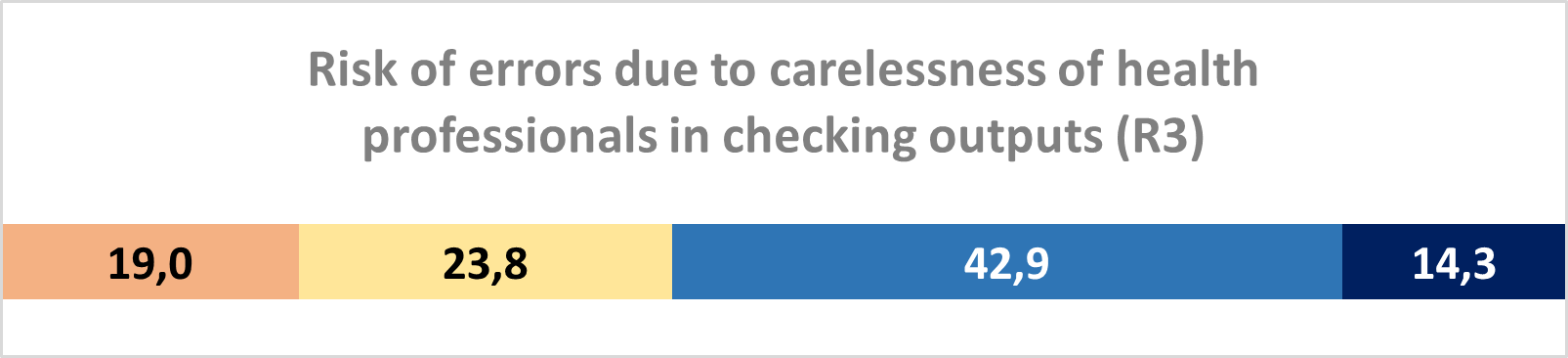 |
| 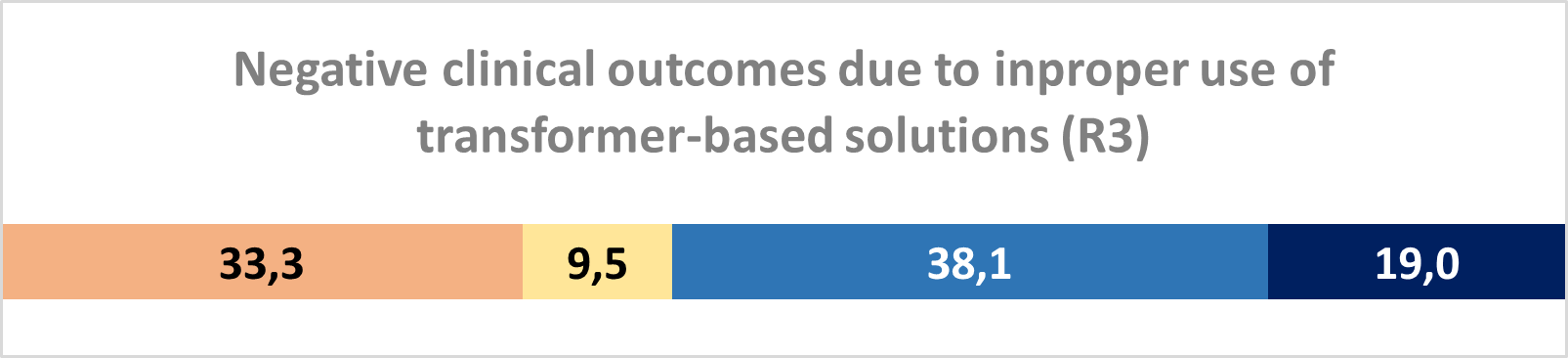 | 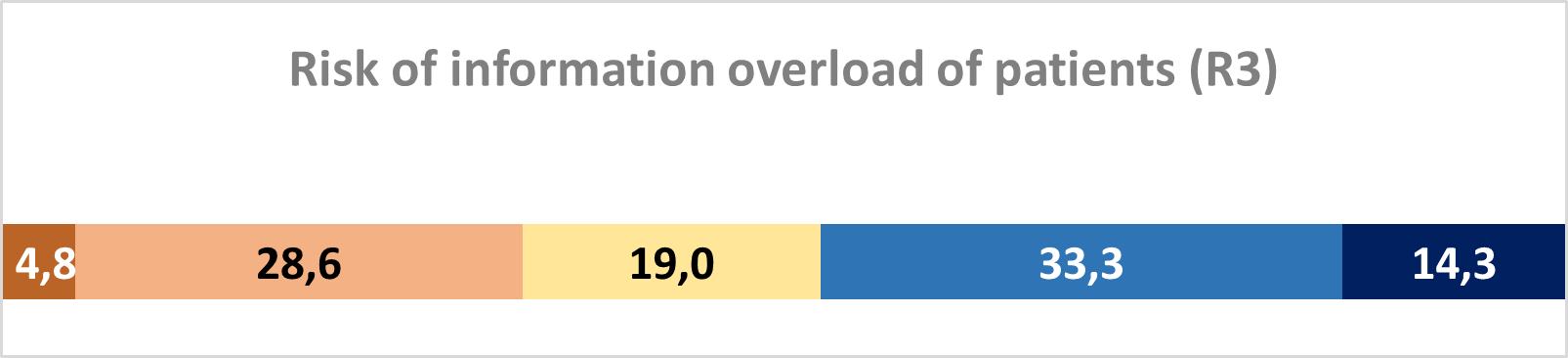 |
| 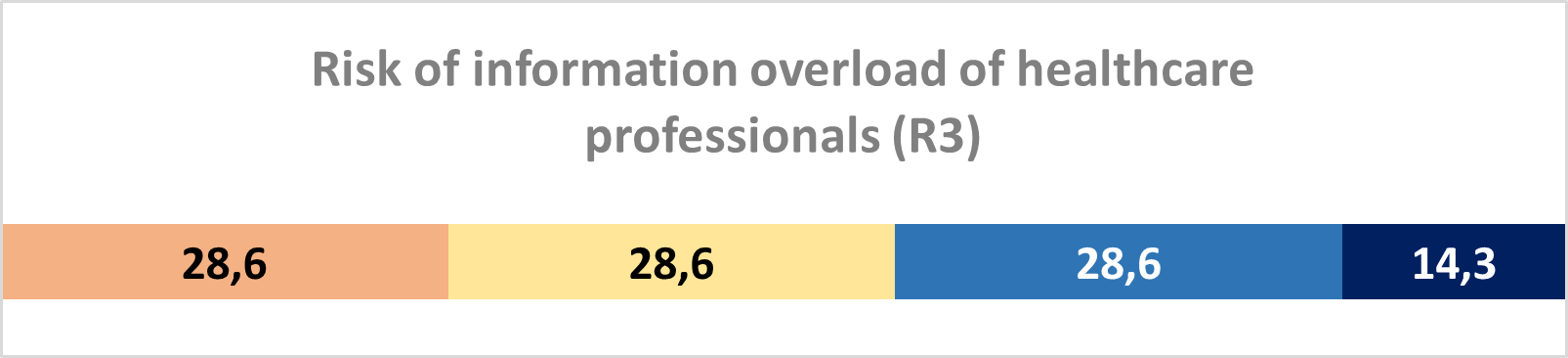 |  |

### V.3.2 Risks in medical profession


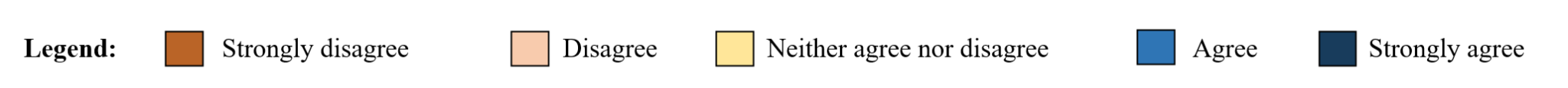


| 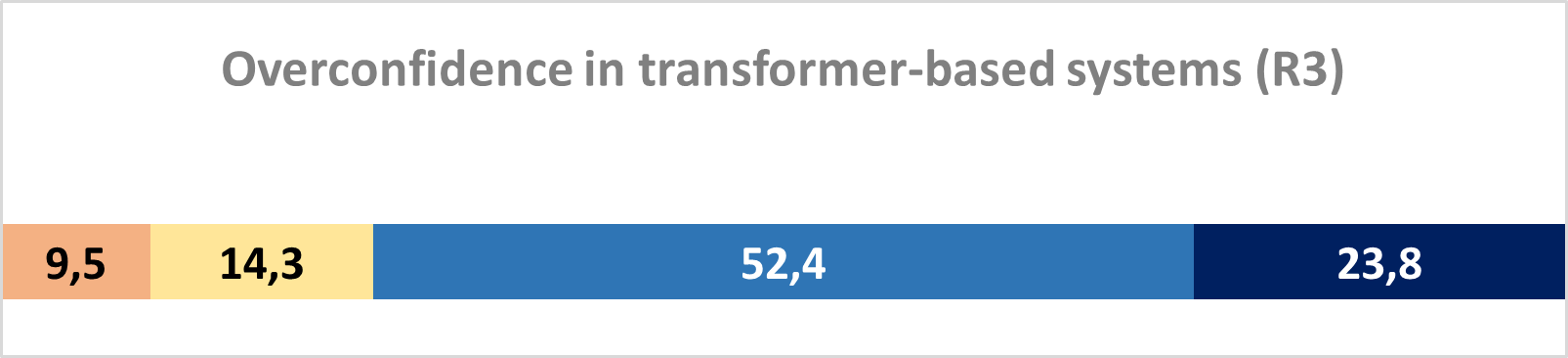 | 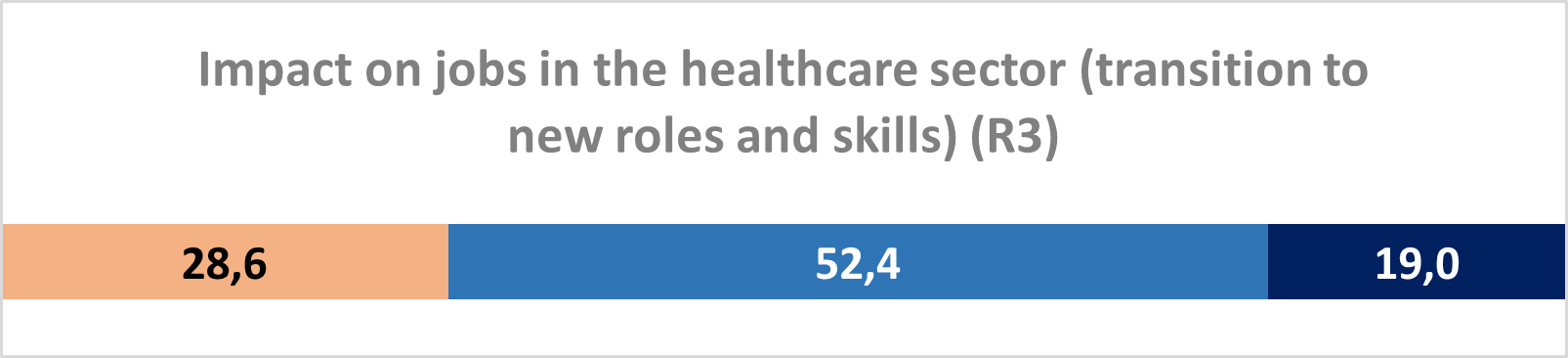 |
| --- | --- |
| 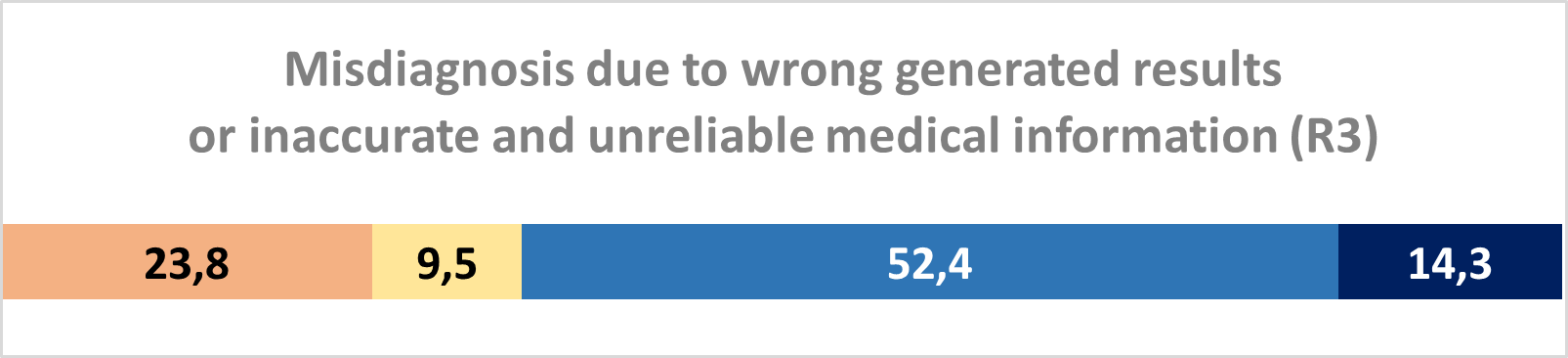 | 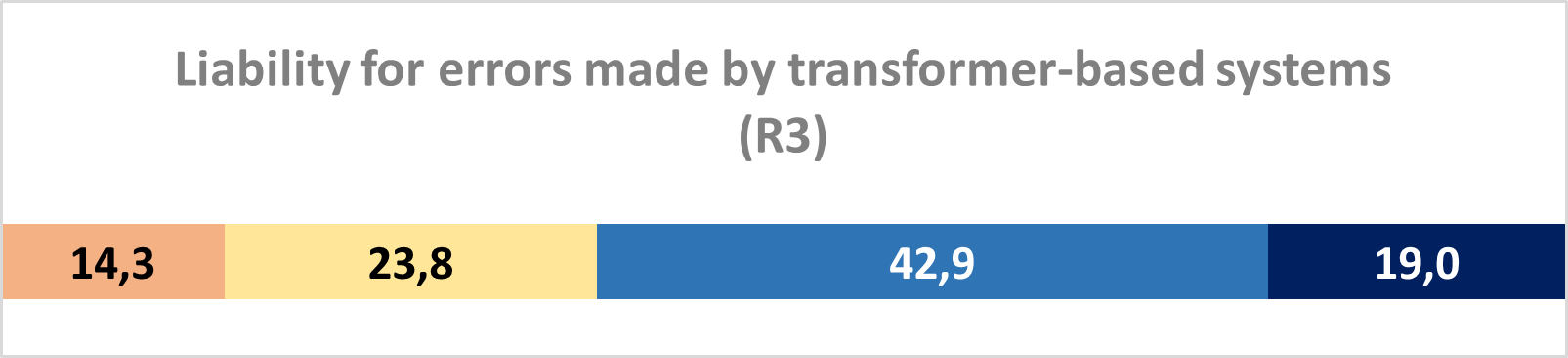 |
| 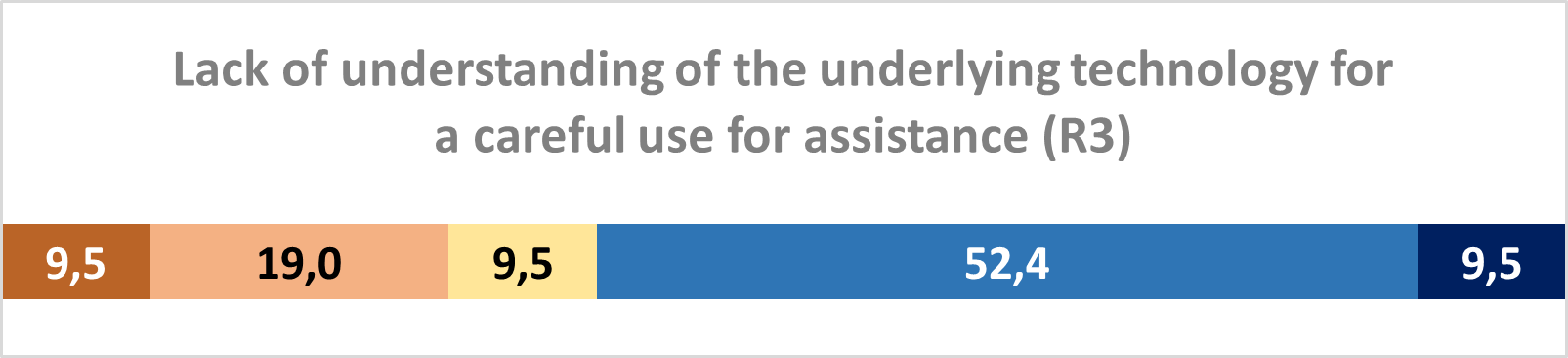 | 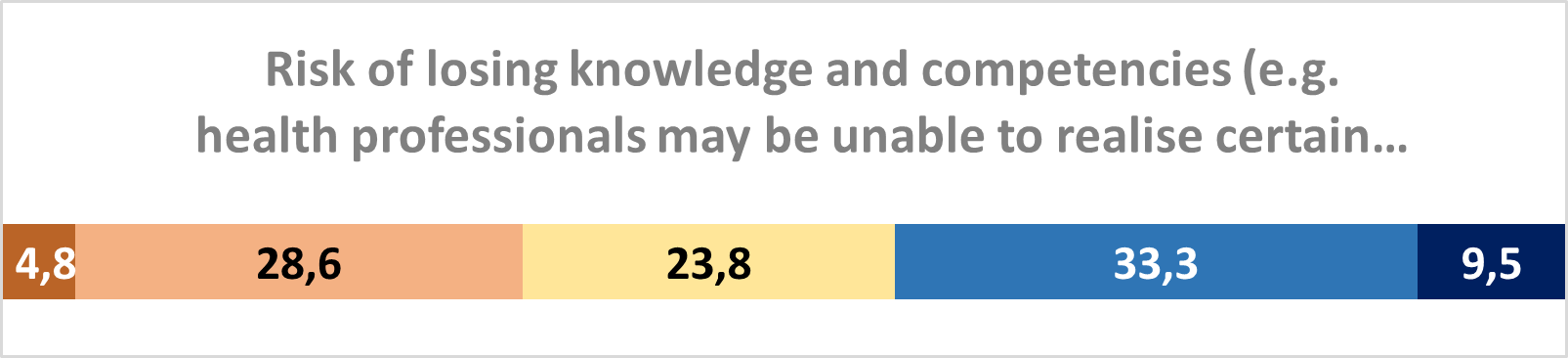 |
| 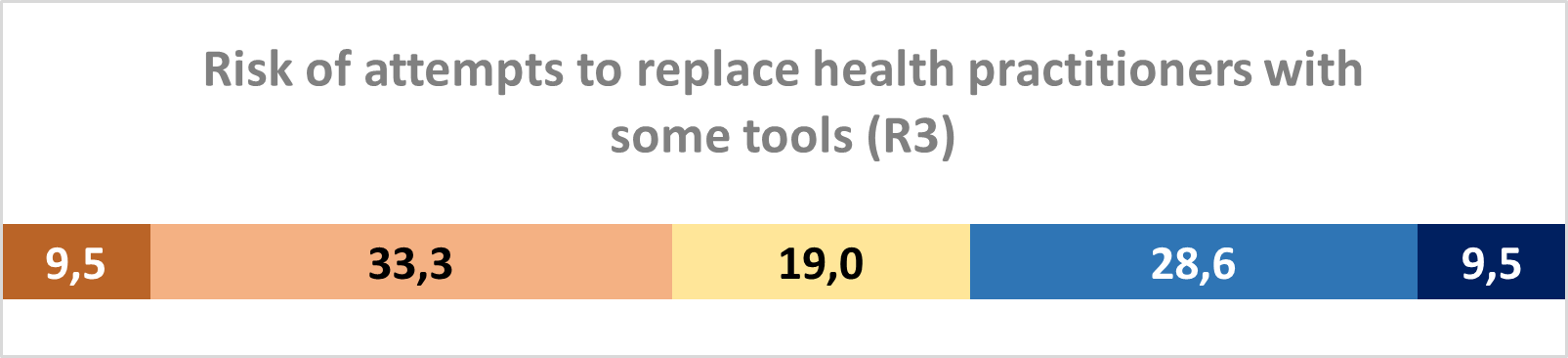 | 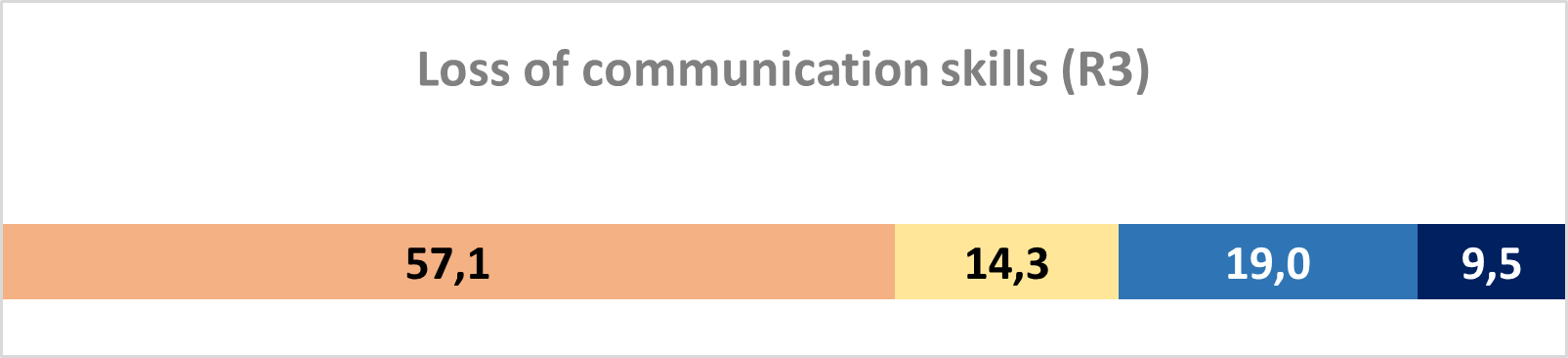 |
| 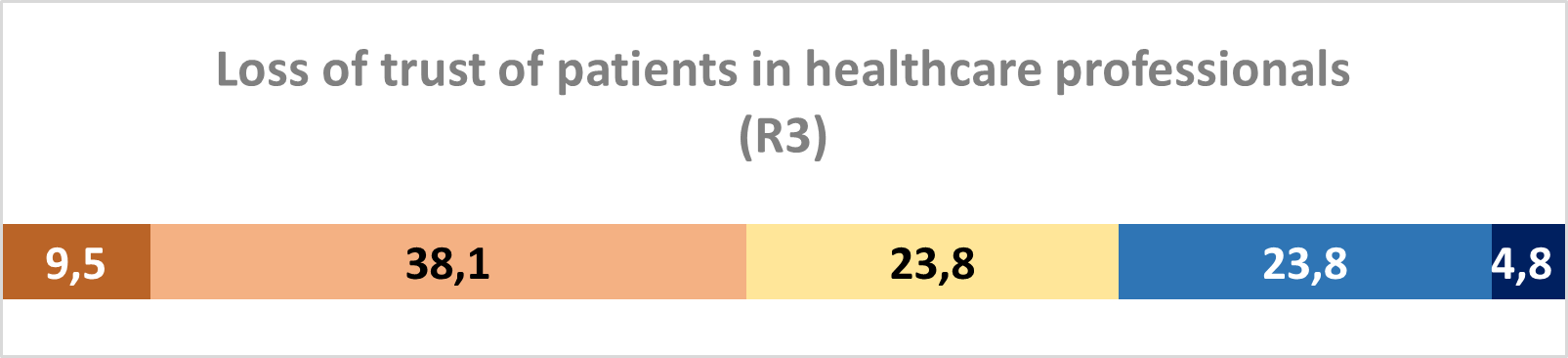 | 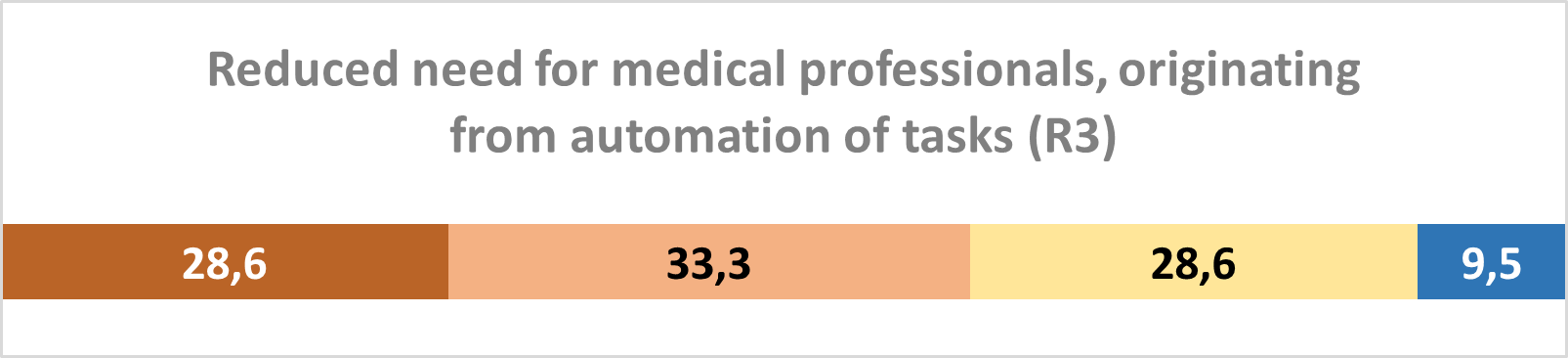 |

### V.3.3 Risks for patients


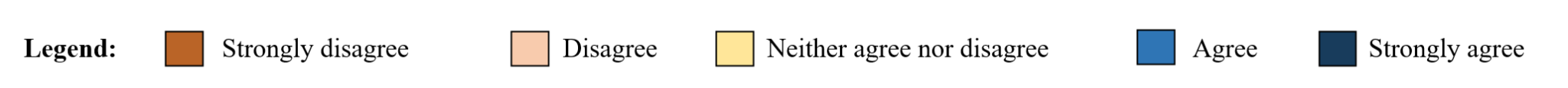


| 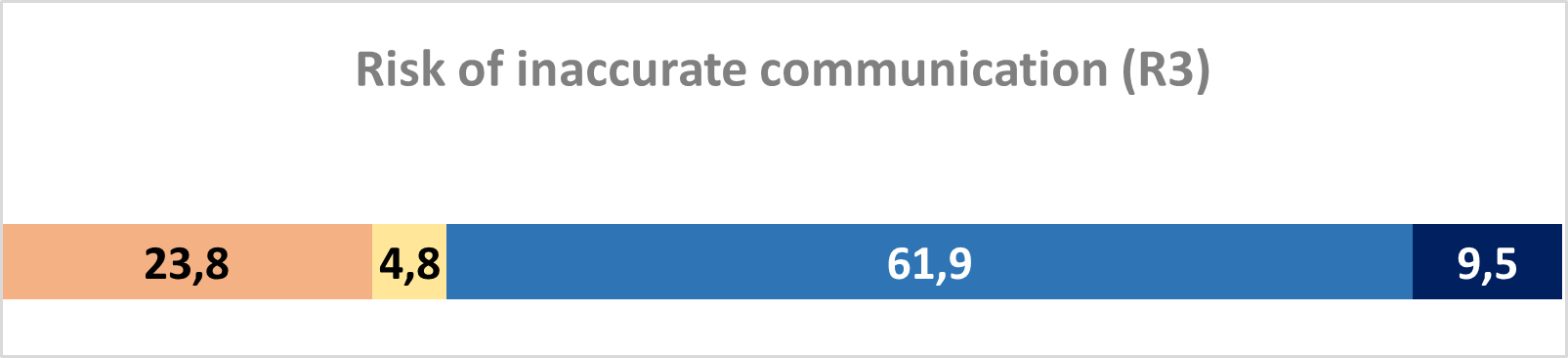 | 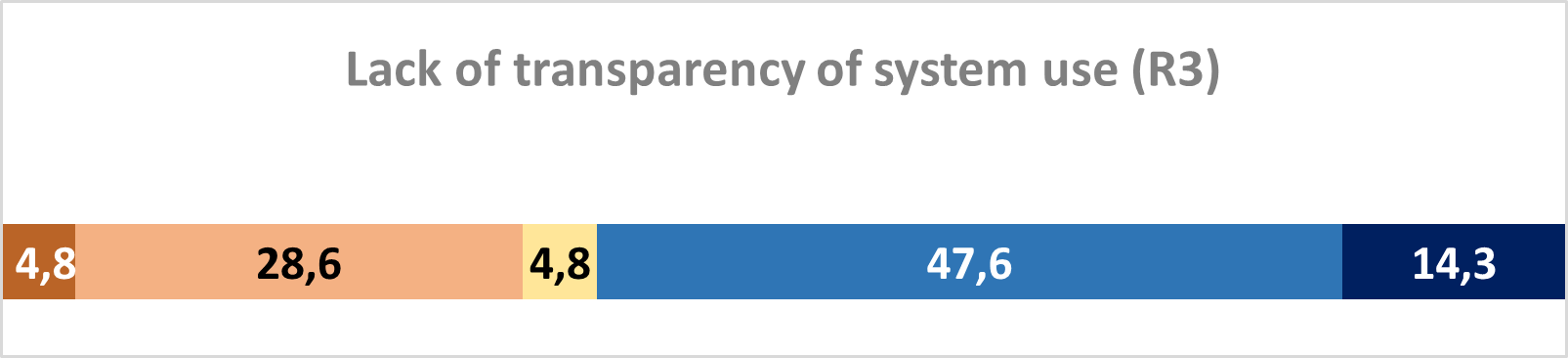 |
| --- | --- |
| 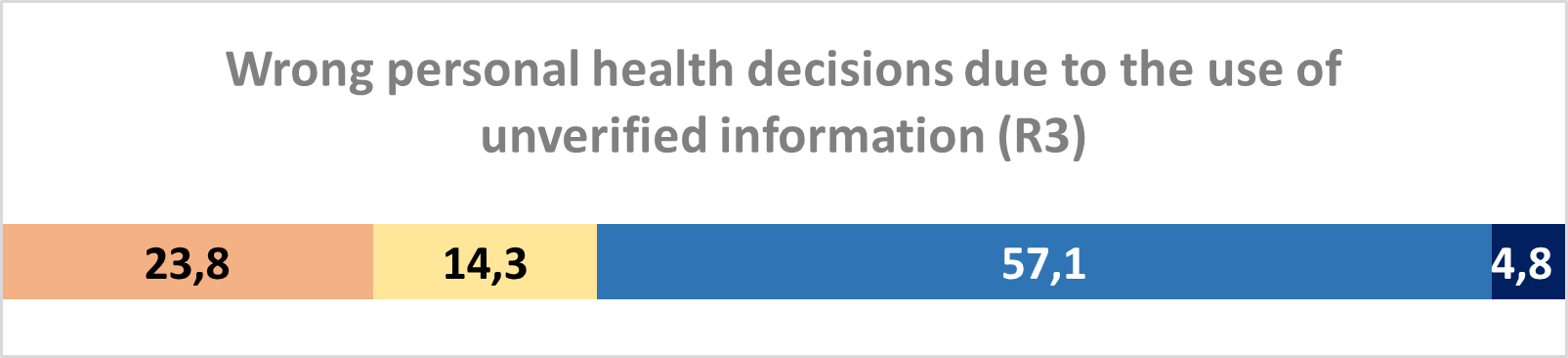 | 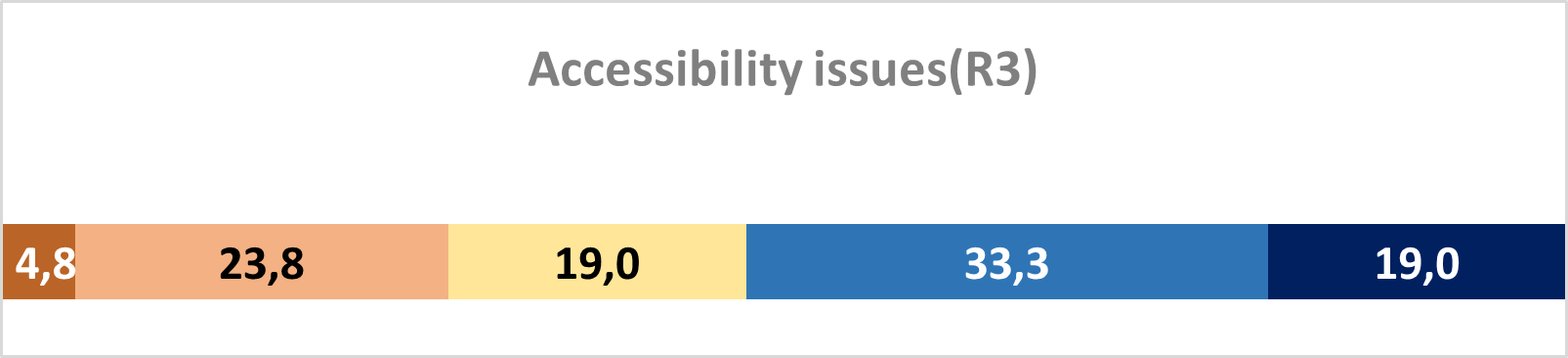 |
| 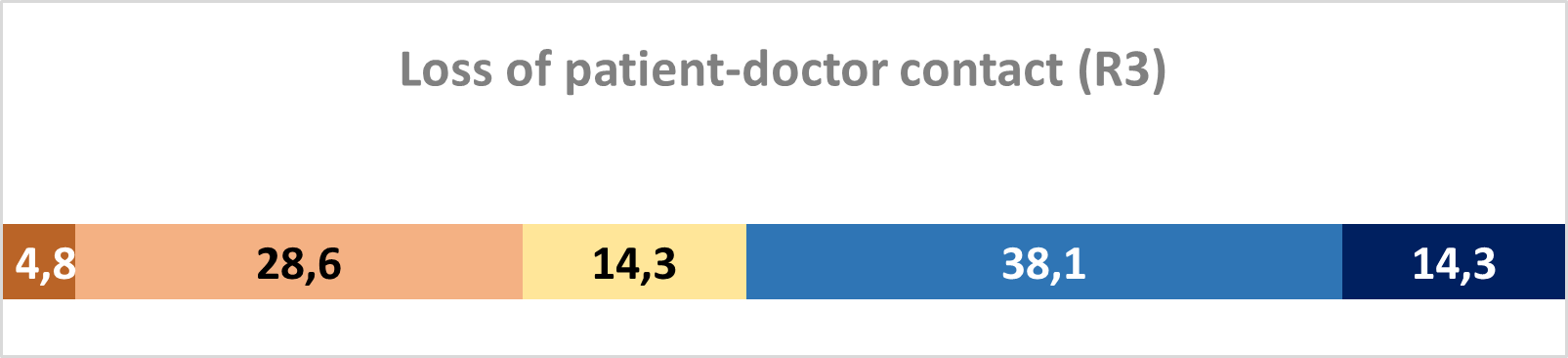 | 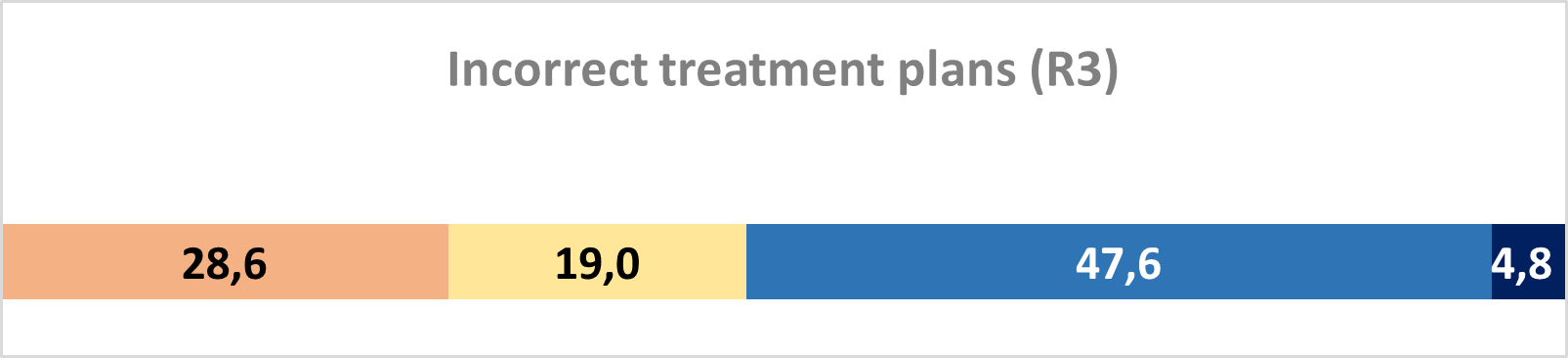 |
| 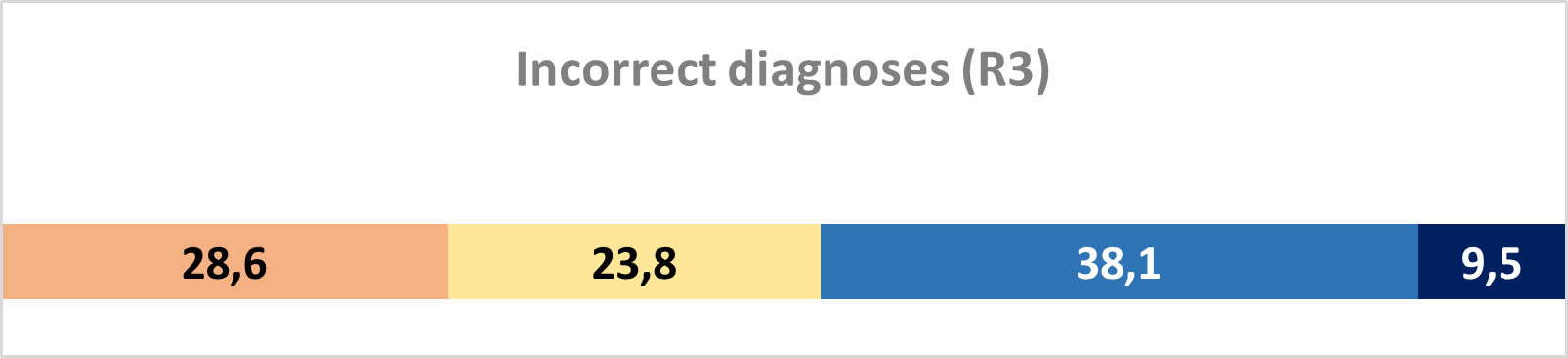 | 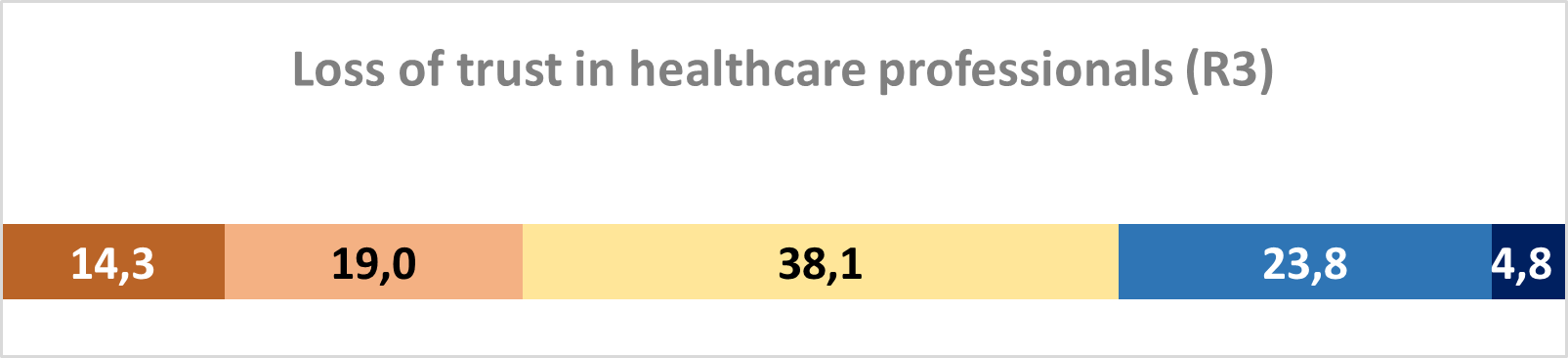 |

### V.3.4 Risks related to data protection


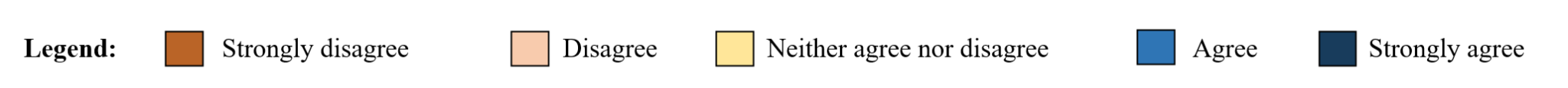


| 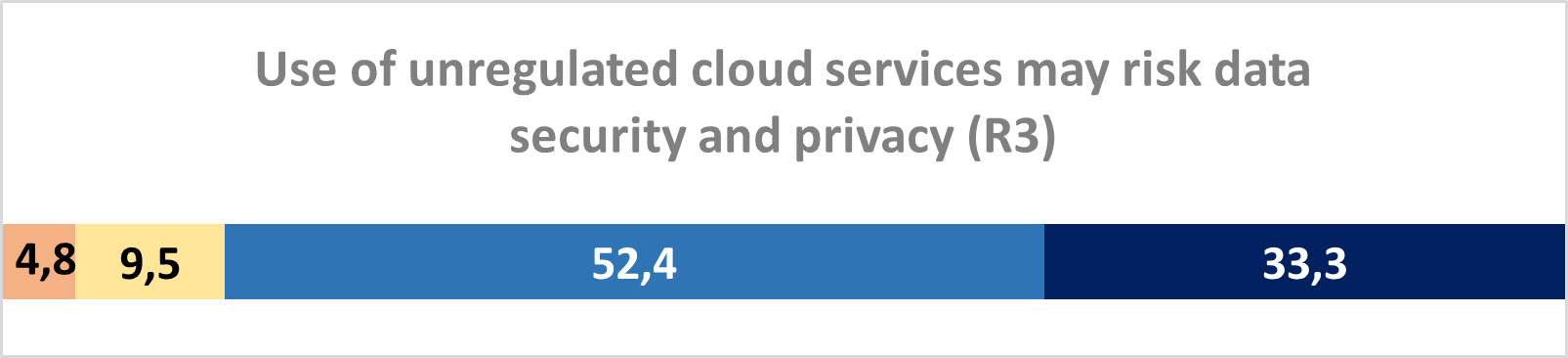 | 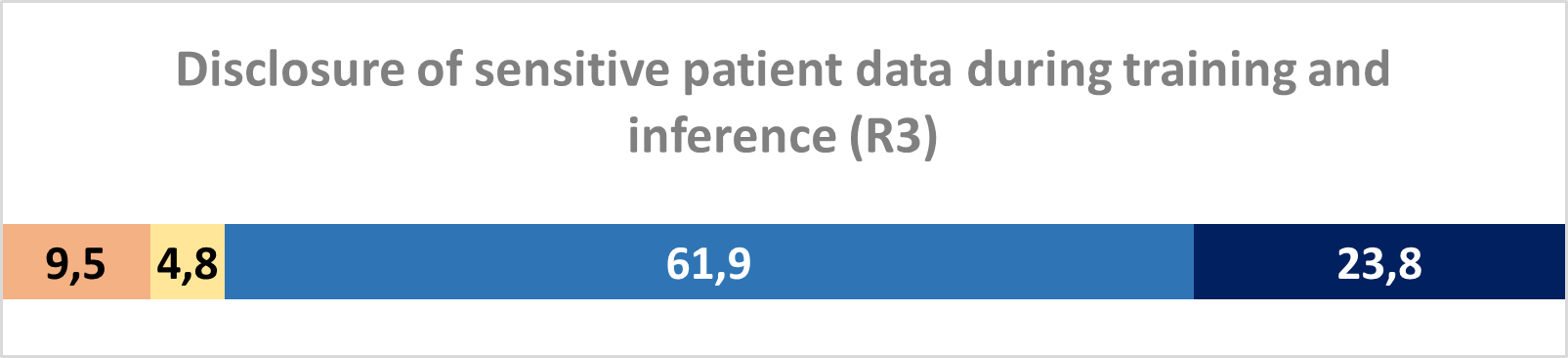 |
| --- | --- |
| 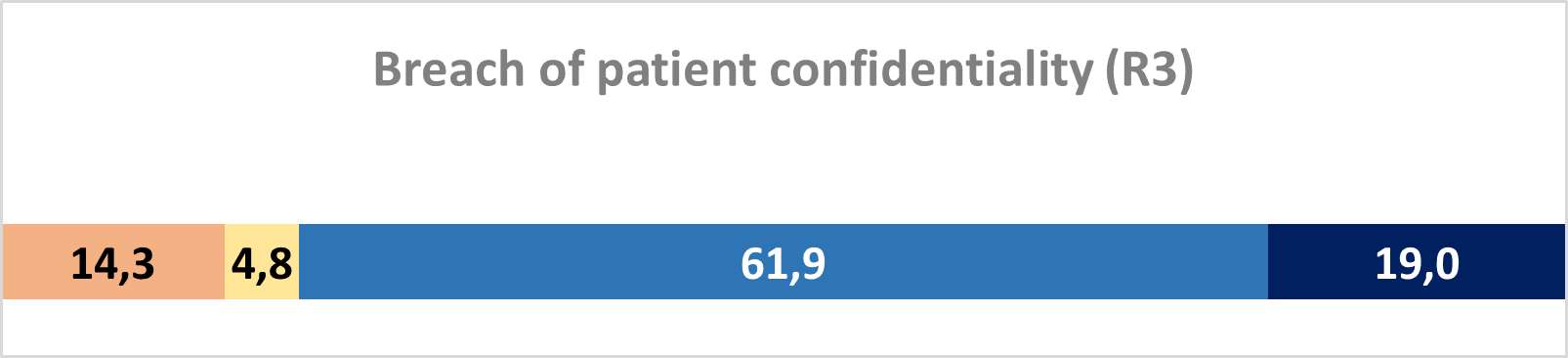 | 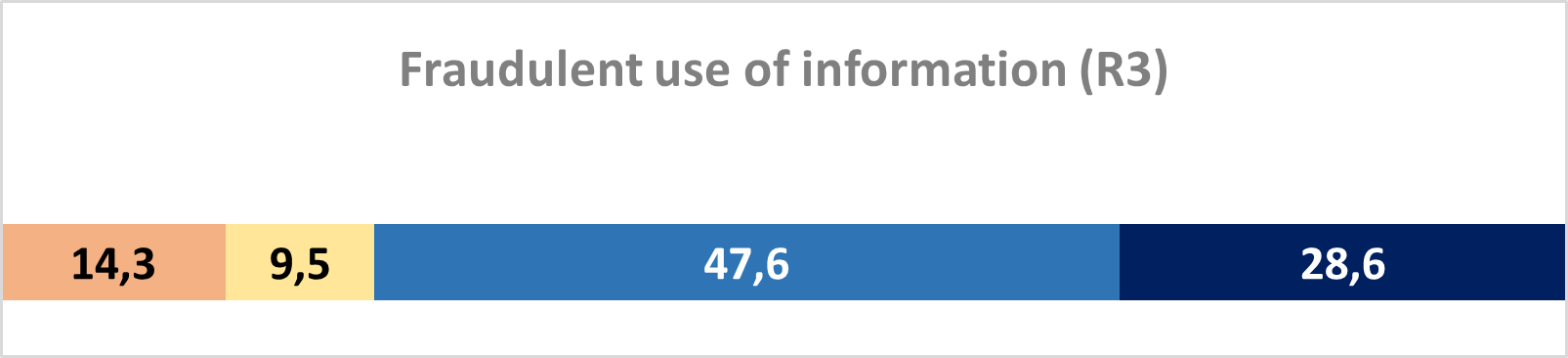 |
| 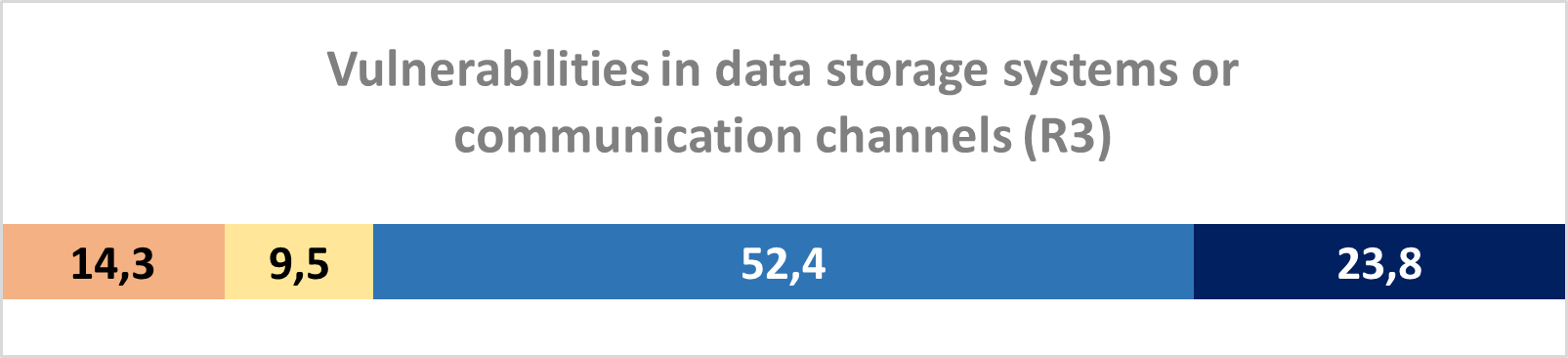 | 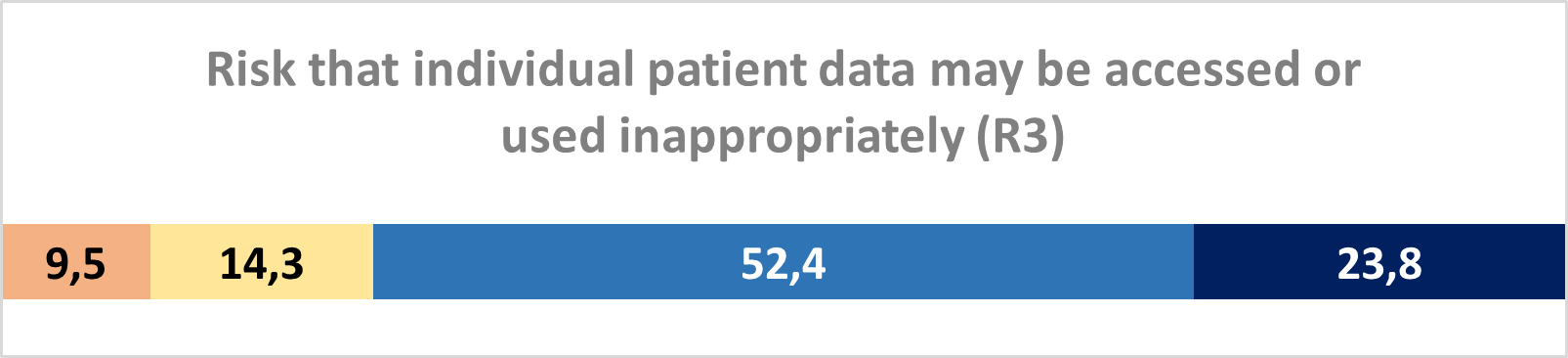 |
| 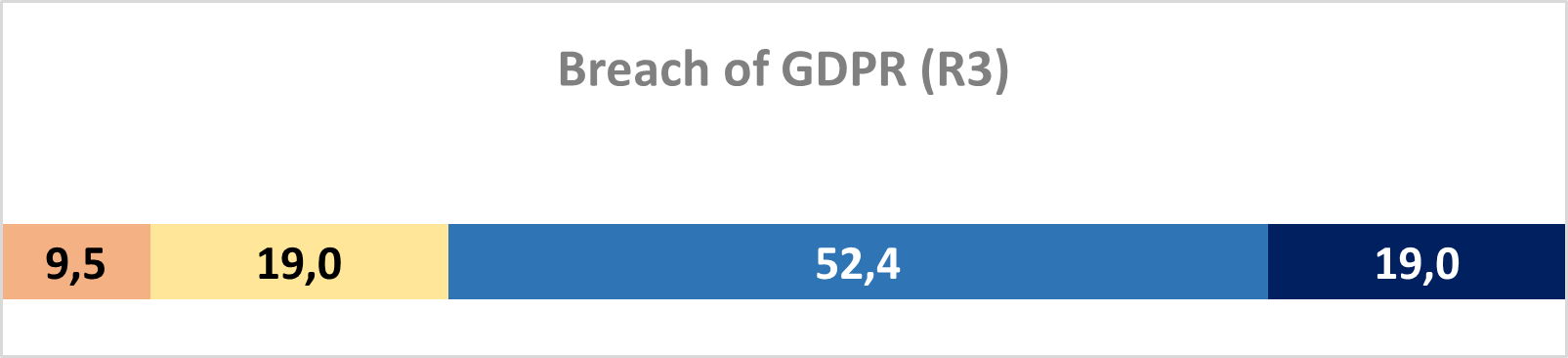 | 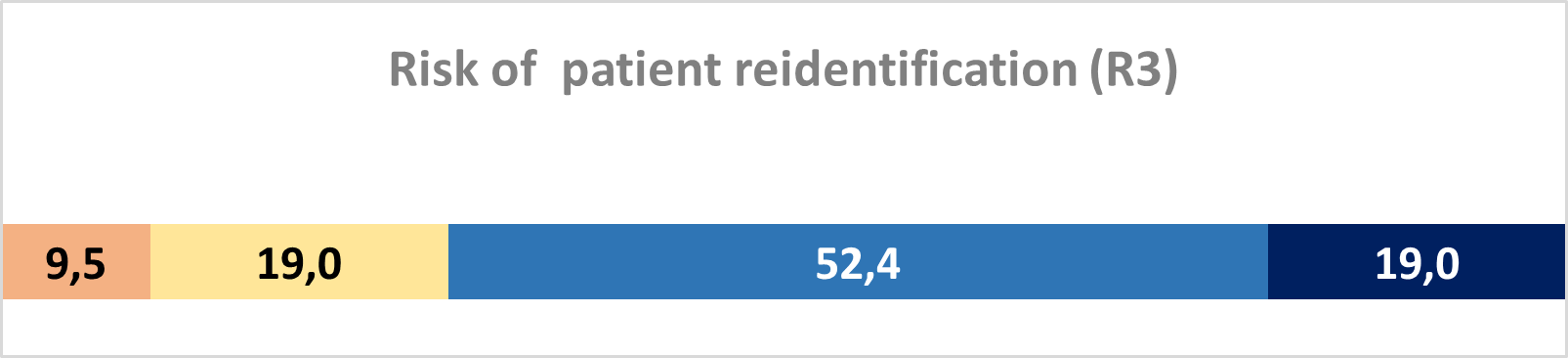 |
| 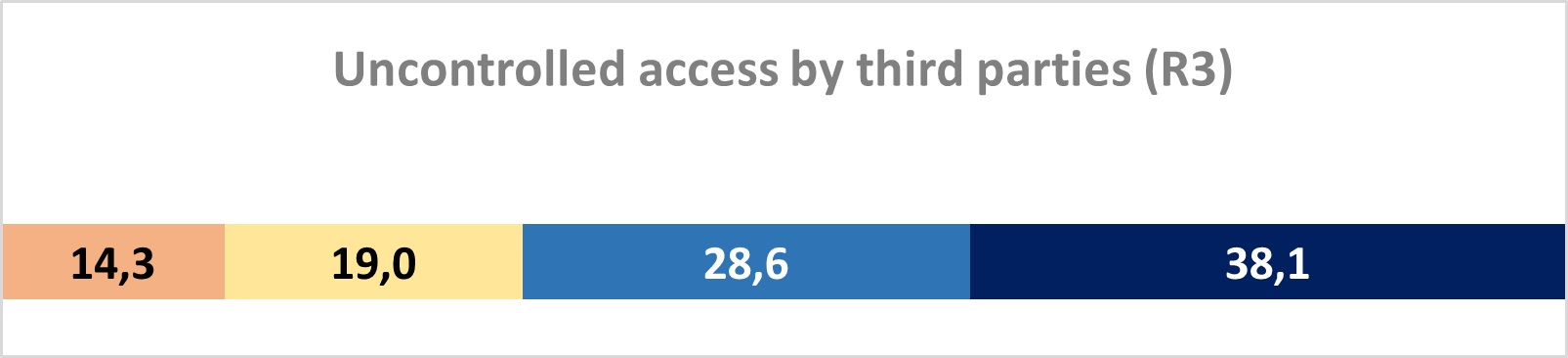 |  |

### V.3.5 Risks for the health IT field


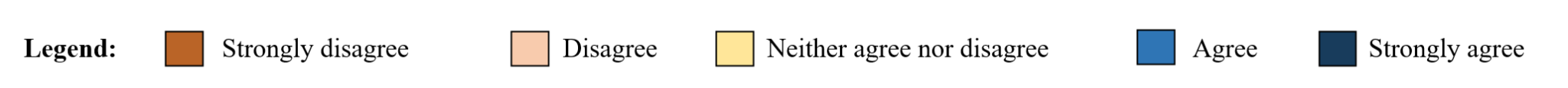


| 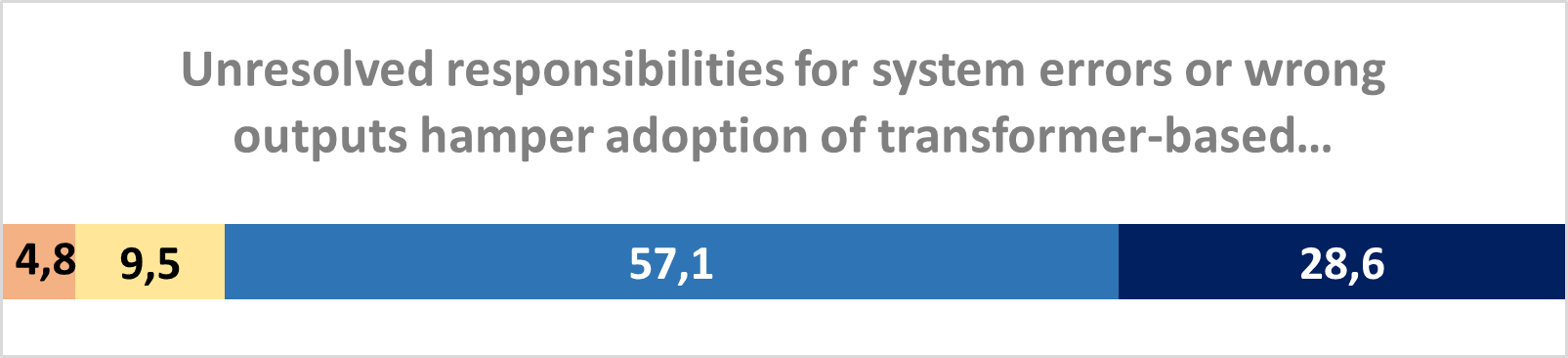 | 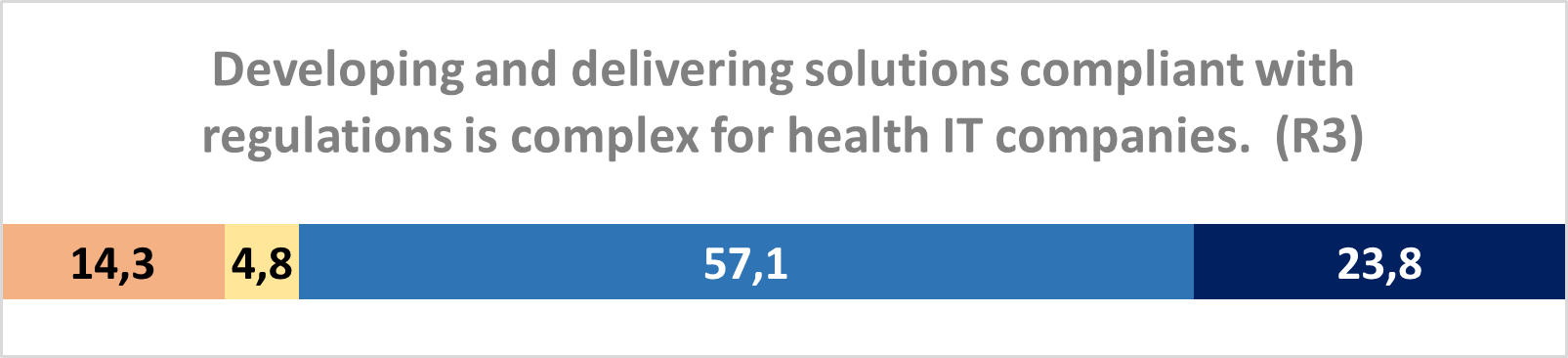 |
| --- | --- |
| 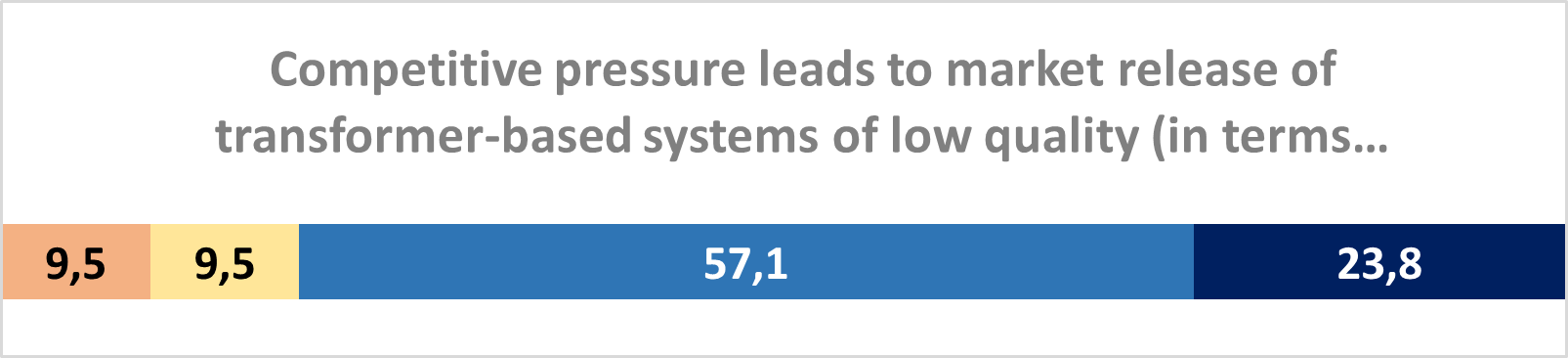 | 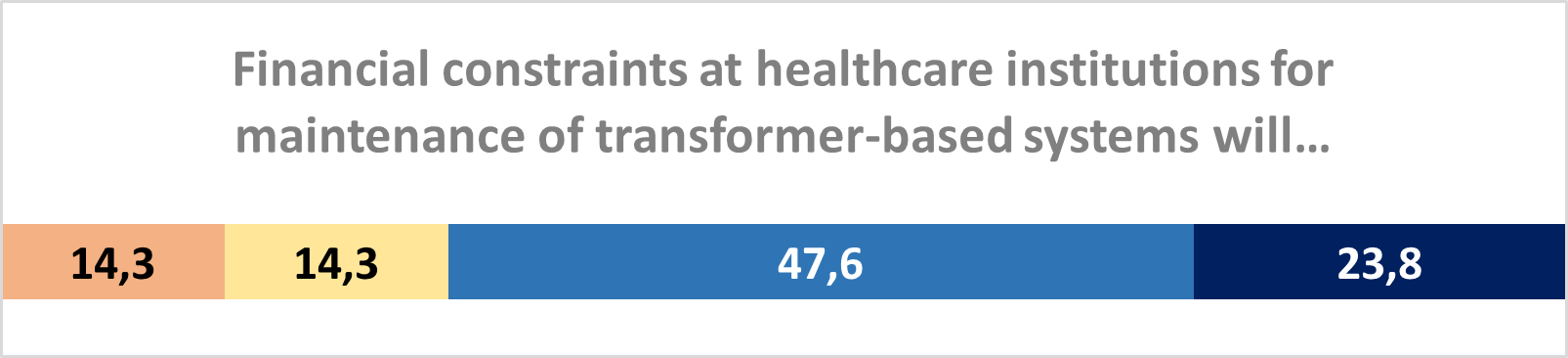 |
| 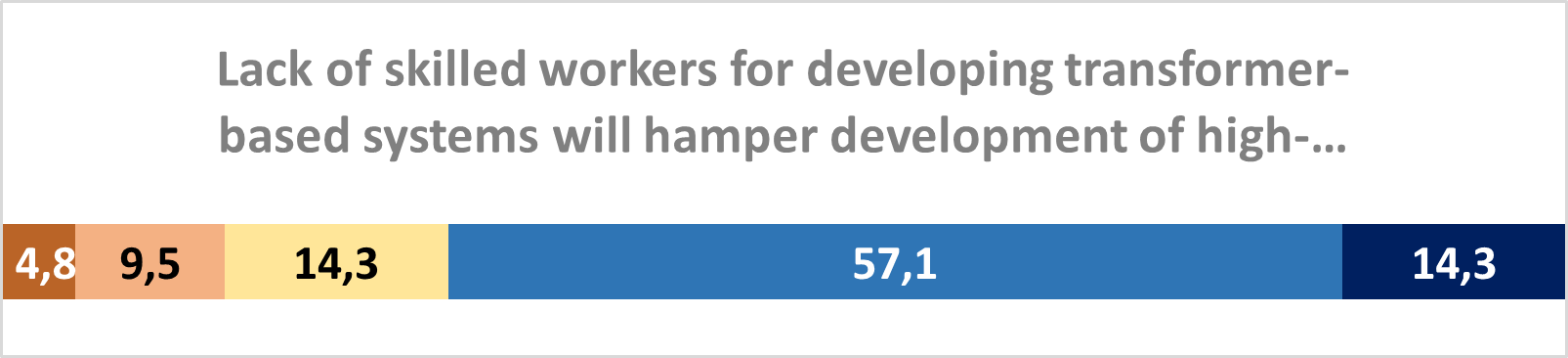 | 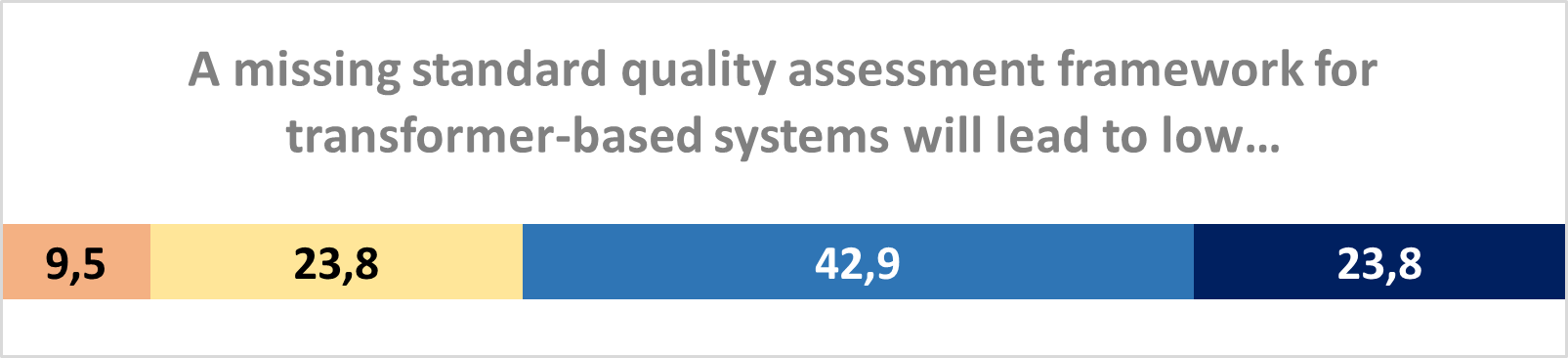 |
| 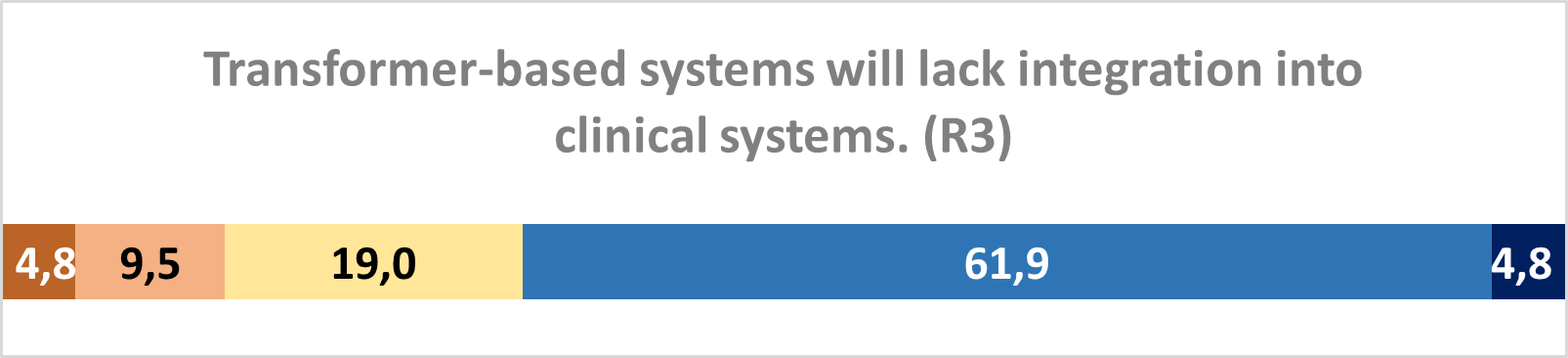 | 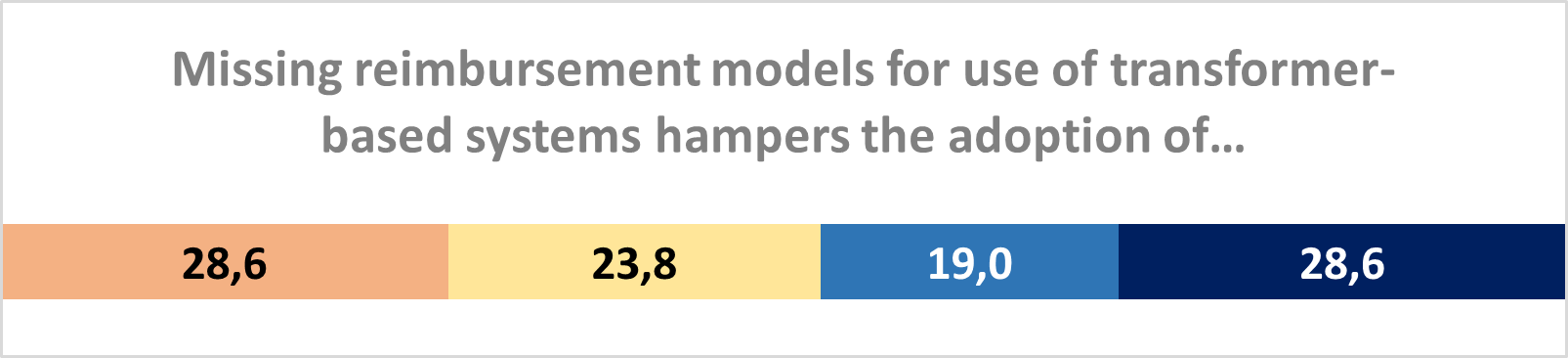 |
| 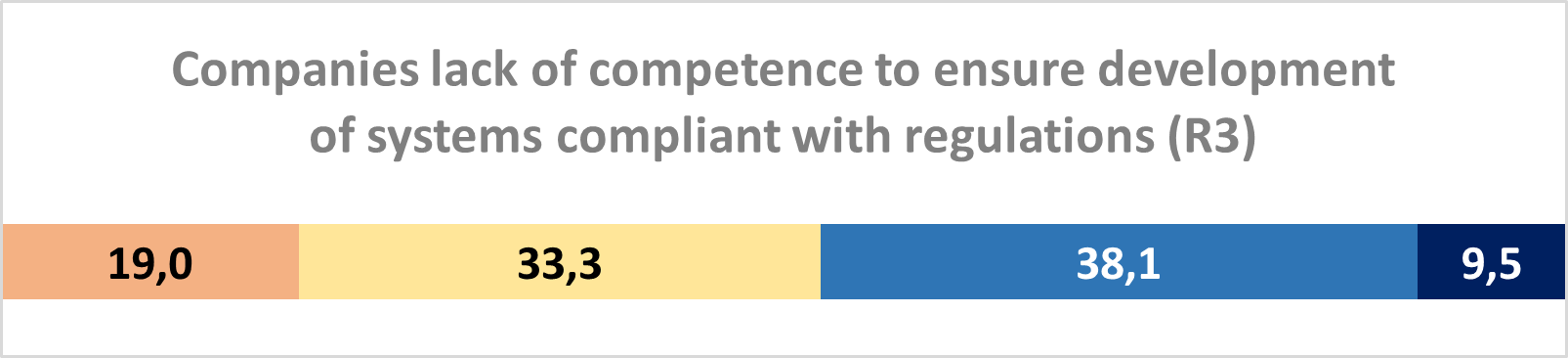 |  |

## V.4 Needs for future adoption and implementation of high-quality LLM-based systems


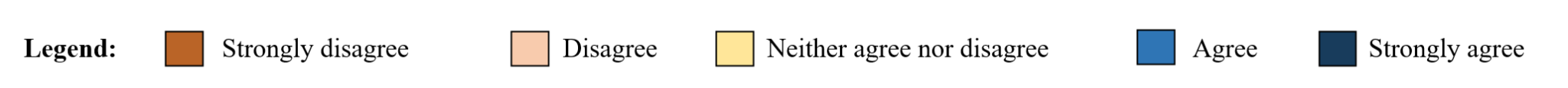


| 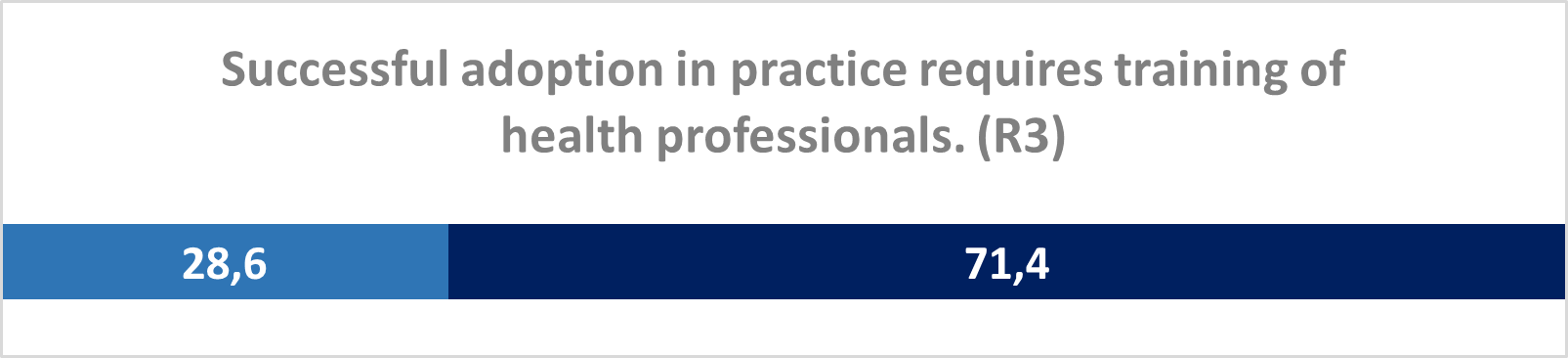 | 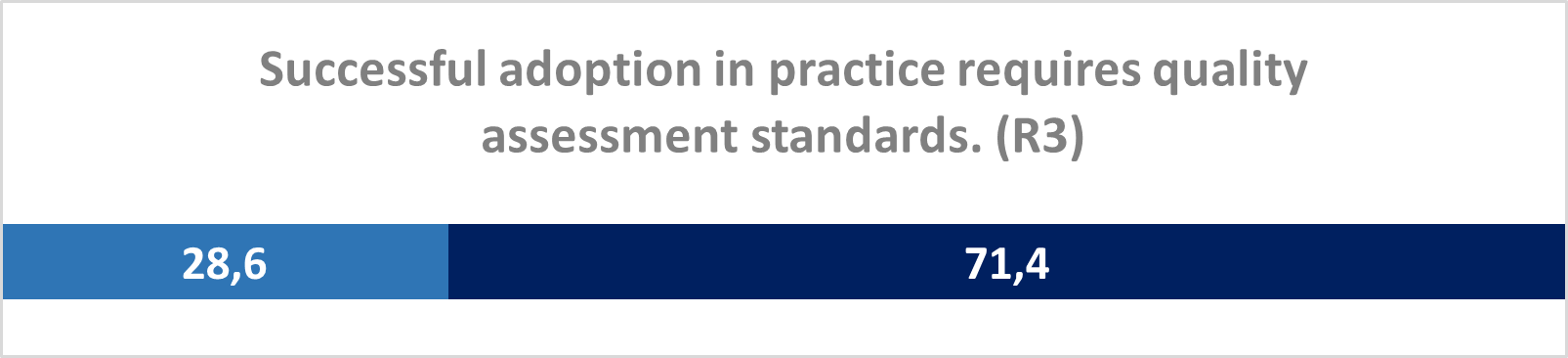 |
| --- | --- |
| 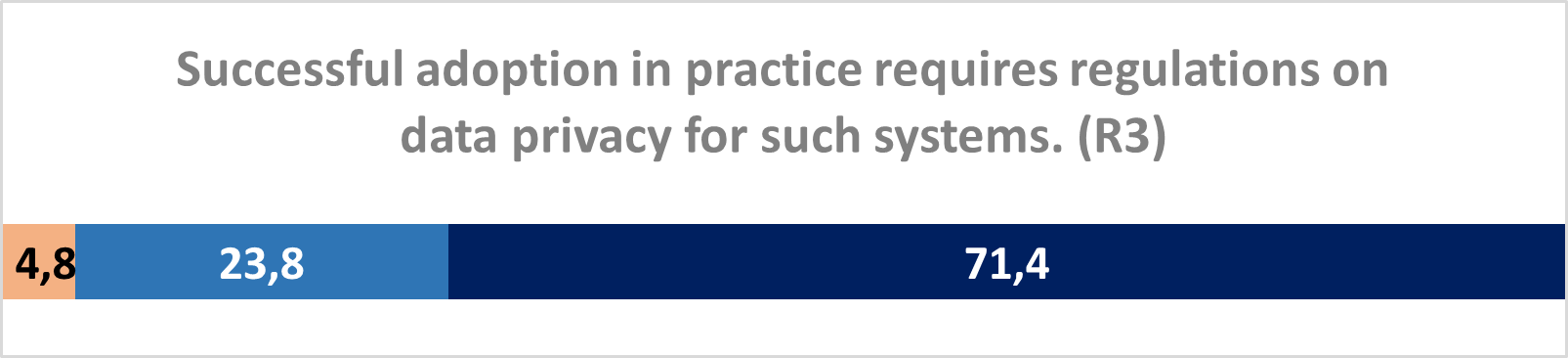 | 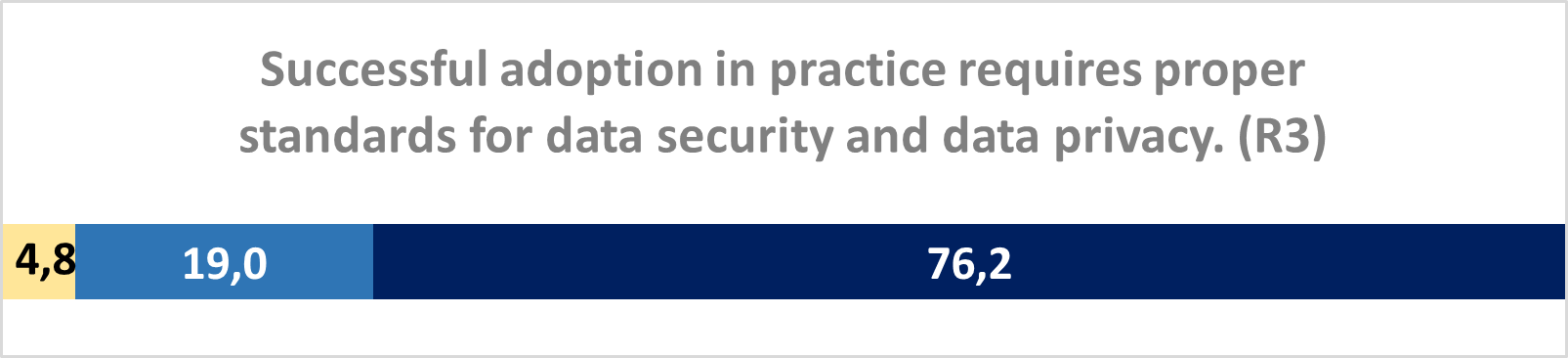 |
| 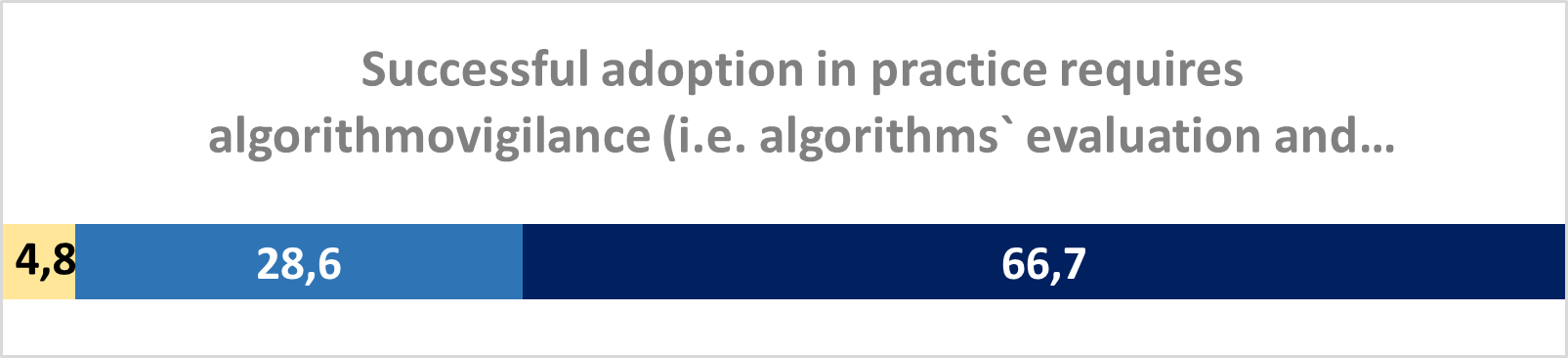 | 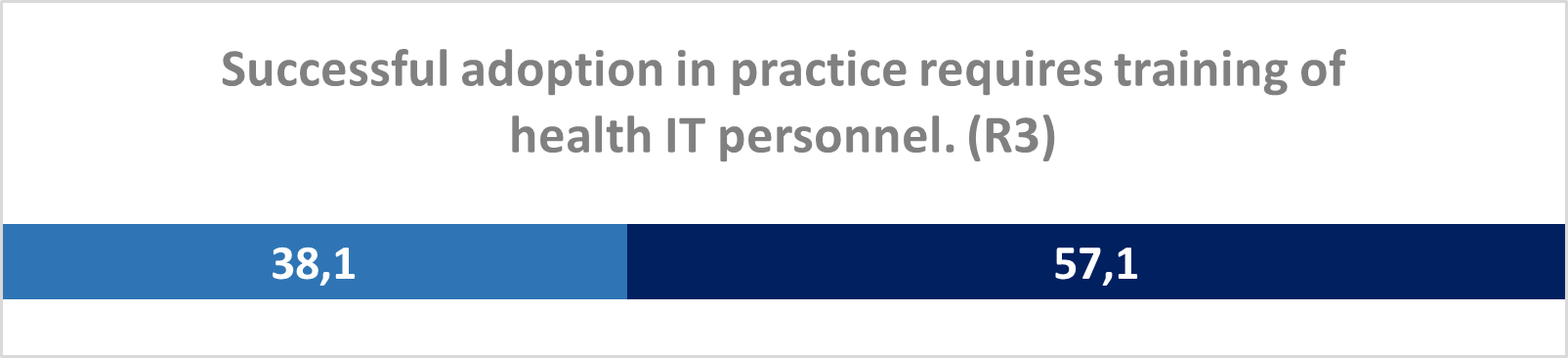 |
| 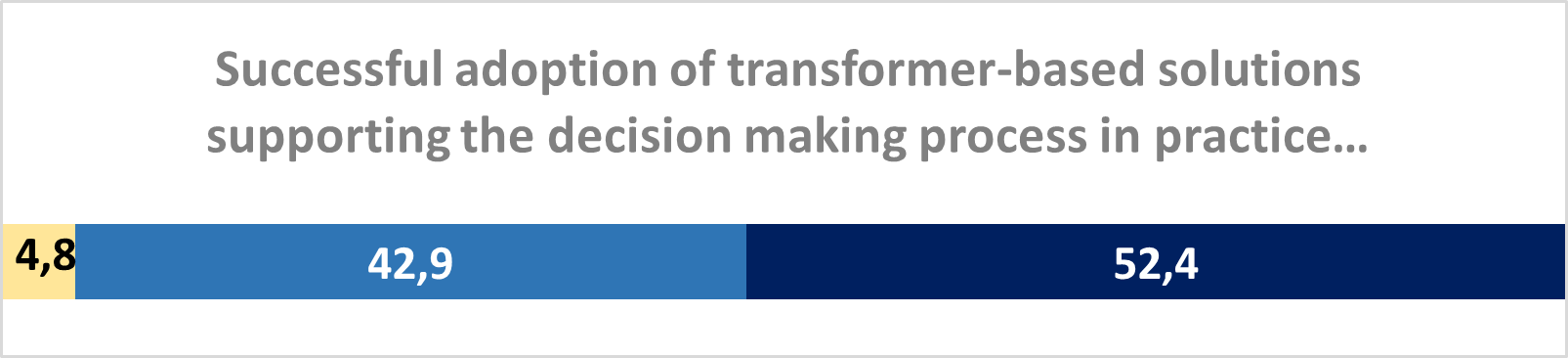 |  |
|  |  |
|  |  |
|  |  |

## V.5 Reliability of systems based on LLMs

|  |  |
| --- | --- |
|  |  |
|  |  |
|  |  |
|  |  |
|  |  |
|  |  |

## V.6 Future of LLMs

|  |  |
| --- | --- |
|  |  |
|  |  |
|  |  |
|  |  |
|  |  |
|  |  |
|  |  |
|  |  |
